# Stimulating Efficiency for Proton Exchange Membrane Water Splitting Electrolyzers: From Material Design to Electrode Engineering

**DOI:** 10.1007/s41918-025-00252-1

**Published:** 2025-09-05

**Authors:** Yu Zhu, Fei Guo, ShunQiang Zhang, Zichen Wang, Runzhe Chen, Guanjie He, Xueliang Sun, Niancai Cheng

**Affiliations:** 1 Institute of New Energy Materials and Engineering, College of Materials Science and Engineering, Fuzhou University, Fuzhou, 350108 Fujian China; 2 Department of Chemistry, University College London, London, WC1H 0AJ UK; 3 College of Materials and Chemical Engineering, Minjiang University, Fuzhou, 350108 Fujian China; 4 Ningbo Key Laboratory of All-Solid-State Battery, Eastern Institute for Advanced Study, Eastern Institute of Technology, Ningbo, 315200 Zhejiang China

**Keywords:** Iridium-based electrocatalysts, Oxygen evolution reaction, Activity and stability, Water electrolysis, Hydrogen energy

## Abstract

Proton exchange membrane water electrolyzers (PEMWEs) are a promising technology for large-scale hydrogen production, yet their industrial deployment is hindered by the harsh acidic conditions and sluggish oxygen evolution reaction (OER) kinetics. This review provides a comprehensive analysis of recent advances in iridium-based electrocatalysts (IBEs), emphasizing novel optimization strategies to enhance both catalytic activity and durability. Specifically, we critically examine the mechanistic insights into OER under acidic conditions, revealing key degradation pathways of Ir species. We further highlight innovative approaches for IBE design, including (i) morphology and support engineering to improve stability, (ii) structure and phase modulation to enhance catalytic efficiency, and (iii) electronic structure tuning for optimizing interactions with reaction intermediates. Additionally, we assess emerging electrode engineering strategies and explore the potential of non-precious metal-based alternatives. Finally, we propose future research directions, focusing on rational catalyst design, mechanistic clarity, and scalable fabrication for industrial applications. By integrating these insights, this review provides a strategic framework for advancing PEMWE technology through highly efficient and durable OER catalysts.

**Graphical Abstract:**

In order to realize the efficient application of the industrial PEMWEs, material design strategies for stimulating the activity and stability capability of OER electrocatalysts are summarized, including (i) morphology/support effects, (ii) structure/phase engineering, (iii) electronic configuration/interaction. Furthermore, the reaction mechanism is deeply clarified, and electrode engineering and challenges of IBEs in practical PEMWE application are focused.

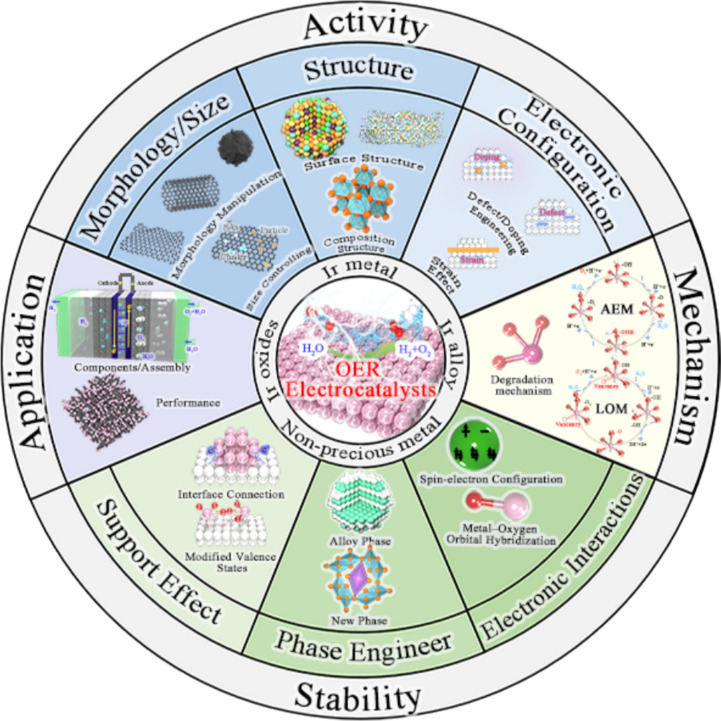

## Introduction

The excessive fossil fuel consumption has led to a variety of environmental problems, including air and water pollution, the greenhouse effect, and disruption of the global carbon balance [1, 2]. Therefore, there is an urgent need to develop efficient and clean energy sources and corresponding energy conversion devices to alleviate these issues [2–5]. Hydrogen (H_2_), as a promising alternative to fossil fuels, possesses high energy density and inherent cleanliness and can be produced from various sources to tackle critical energy challenges [6–9]. For example, more than 30 economies have published hydrogen strategic roadmaps, legislation, and vision documents to promote the development of a hydrogen economy. In China’s 14th Five-Year Plan, the comprehensive development of the entire hydrogen industry is emphasized, with a target to increase green hydrogen production to 10% of total hydrogen output [10]; the US Department of Energy (DOE) plans to produce 50 million tons of clean hydrogen annually by 2050, projected to reduce US greenhouse gas emissions by approximately 10% [11]; the European Union estimates that hydrogen could meet about 25% of its total energy demand (2 250 TWh) by 2050 [12]. To achieve these goals, hydrogen production via water-splitting electrolyzers is essential.

**Fig. 1:**
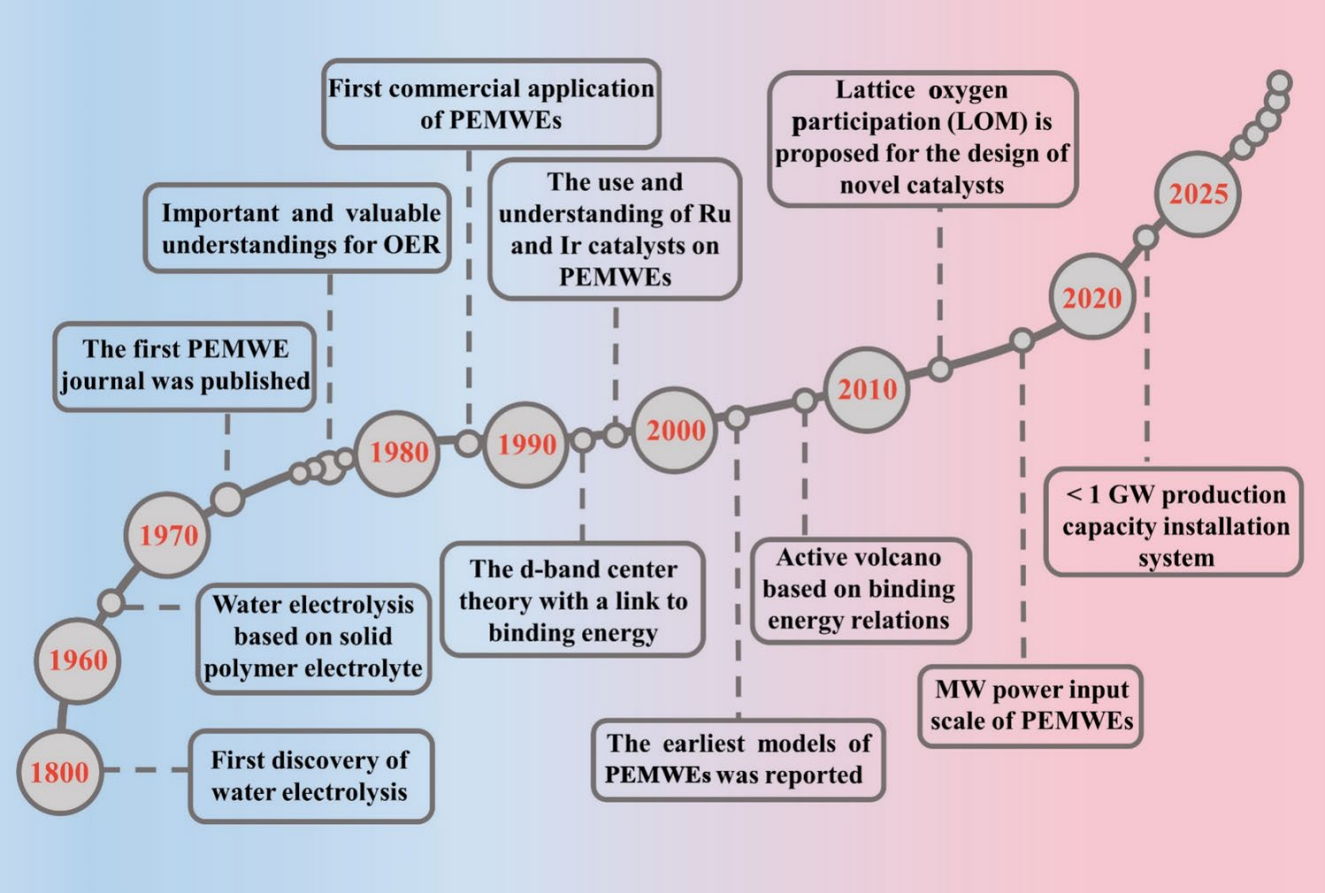
Timeline of the development and theory of PEMWEs

Among the different types of water-splitting electrolyzers [13–15], proton exchange membrane water electrolyzers (PEMWEs) represent a pivotal technology for scaling green hydrogen production to meet carbon neutrality targets by 2050 [16]. Significant progress has been made in advancing PEMWEs for practical applications and deepening theoretical understanding (Fig. 1). The PEMWEs feature compact system designs, operate at high current densities with low ohmic losses, and produce high-purity hydrogen [17, 18], making them ideal for continuous energy input. Especially, the characteristics of fast electrical response enable large-scale compatible integration with intermittent renewable energy sources (e.g., wind and solar power) [19, 20]. However, the complex multi-step reactions, high electrode overpotentials, and sluggish reaction kinetics in acidic media reduce energy efficiency [21, 22]. Therefore, electrocatalysts with high activity and stability are essential for improving oxygen evolution reaction (OER) kinetics and overall PEMWE efficiency.

To date, Ru- and Ir-based electrocatalysts (IBEs) remain the most advanced OER catalysts in acid media [23, 24]. Ru-based catalysts exhibit excellent OER activity, but their stability is compromised by the formation of volatile RuO_4_ during operation [25, 26]. In contrast, benchmark IBEs (e.g., commercial IrO_2_, Ir/C) offer superior stability and are currently employed in PEMWEs due to their balanced activity and durability under harsh conditions. However, their activity and stability remain suboptimal due to inadequate stabilization of reactive intermediates (e.g., *O, *OH) and irreversible dissolution under industrial operating conditions [27, 28]. In addition, the scarcity and high cost of Ir (∼US$60 670 kg^−1^) hinder its large-scale application in PEMWEs [29, 30]. Additionally, discontinuities and complex multi-mass transfer within the catalyst layer, involving catalyst particles, perfluorosulfonic acid (PFSA) ionomer electrolytes, and their interfaces, are bottlenecks limiting the performance of PEMWEs [31]. Therefore, the optimization of the catalyst and catalyst layer design to form an effective electrochemically active interface remains a long-term focus in the large deployment of PEMWEs [32, 33].

Within this framework, the industrial application of PEMWEs pivots on the development of catalyst-coated membranes (CCMs) and must address four critical aspects. (i) Boosting activities—minimizing the use of Ir in IBEs while still obtaining comparable or even better catalytic activity; (ii) enhancing stabilities—the long-term stability performance in acid and high-potential regions should be given sufficient attention to meet industrial needs [34–36]; (iii) clarifying the mechanism—understanding the catalytic mechanism and the operation of the active sites to facilitate the rational design of high-performance IBEs; and (iv) optimizing core components of PEMWEs—the structure of IBEs in membrane electrode assemblies (MEA) is rationally designed to obtain an ideal trade-off between interfacial reactions and transport of various reactants.

Overall, the structure of the catalytic layer in PEMWEs is closely related to the intrinsic properties of the catalytic materials and interactions. Previous reviews have predominantly focused on isolated aspects of catalyst activity or stability [37–39]. In contrast, recent studies advance integrated strategies that concurrently enhance both properties. This review uniquely synthesizes these developments, bridging material design with catalytic layer optimization. By consolidating advances and proposing innovative perspectives, it aims to inspire next-generation OER electrocatalysts for PEMWEs.

**Scheme 1:**
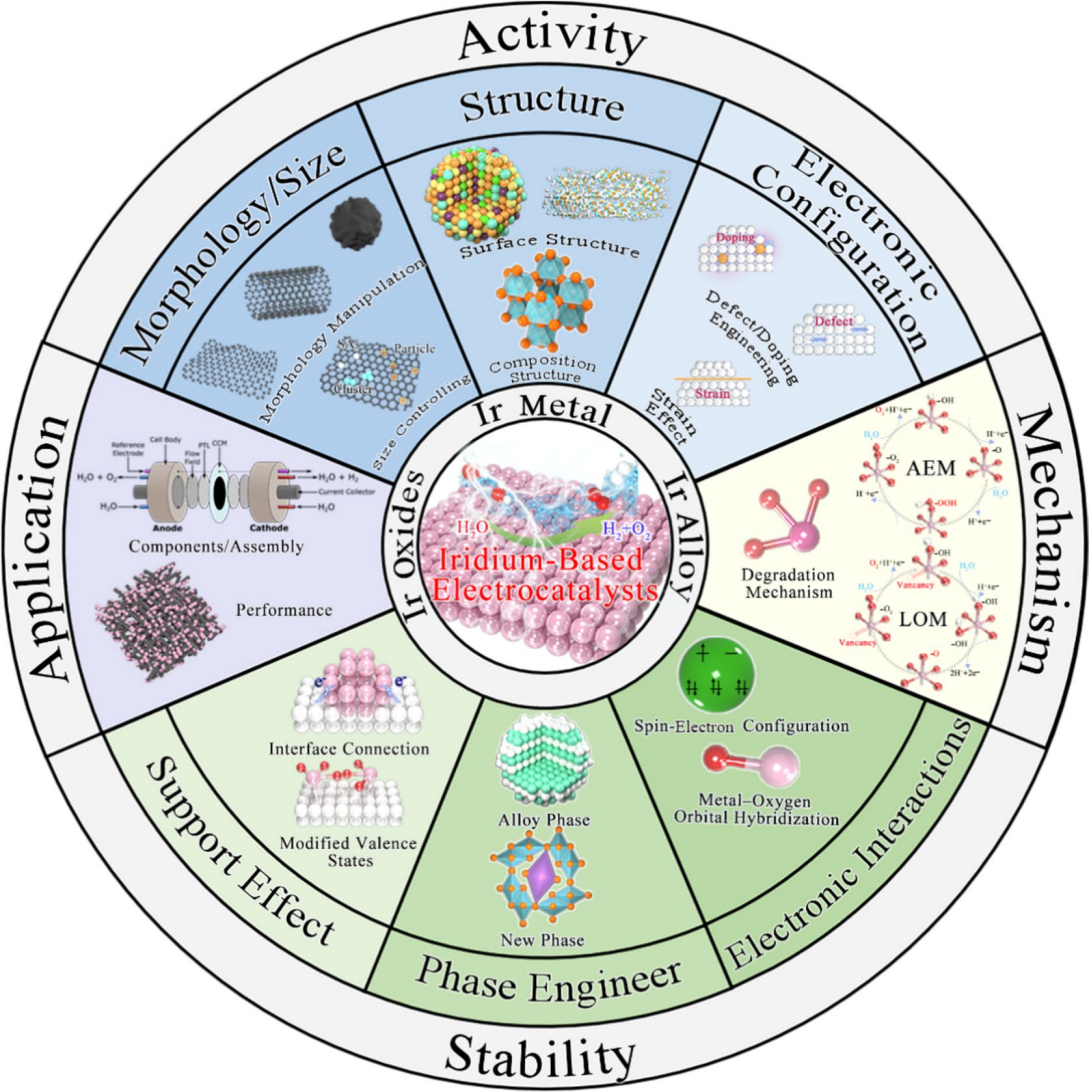
Material strategies of activity and stability for IBEs and PEMWEs application in this review

In this paper, the recent research progress of IBEs in acidic OER is comprehensively reviewed, and the innovative material strategies of stimulating the activity and stability capability of IBEs for meeting the industrial application of PEMWEs are summarized (Scheme 1). Various recent OER mechanisms proposed in acid to clarify the activity and degradation mechanisms of Ir species are firstly focused on, providing foundational principles for catalyst design. The state-of-the-art IBEs in acidic media are discussed from two aspects to highlight material strategies for improving activity (e.g., morphology/size design, structure optimization, and electron regulation) and stability (e.g., support effects, phase engineering, and electron interaction). The development status and the industrial application of IBEs in PEMWEs are also deeply discussed, along with advances in non-precious metal electrocatalysts. Furthermore, key technical challenges in the development of OER electrocatalysts are analyzed, and several future research directions are proposed to address these challenges, aiming to achieve highly active and stable OER catalysts for PEMWEs.

## Mechanistic Understanding of OER

### Adsorbate Evolution Mechanism (AEM)

**Fig. 2:**
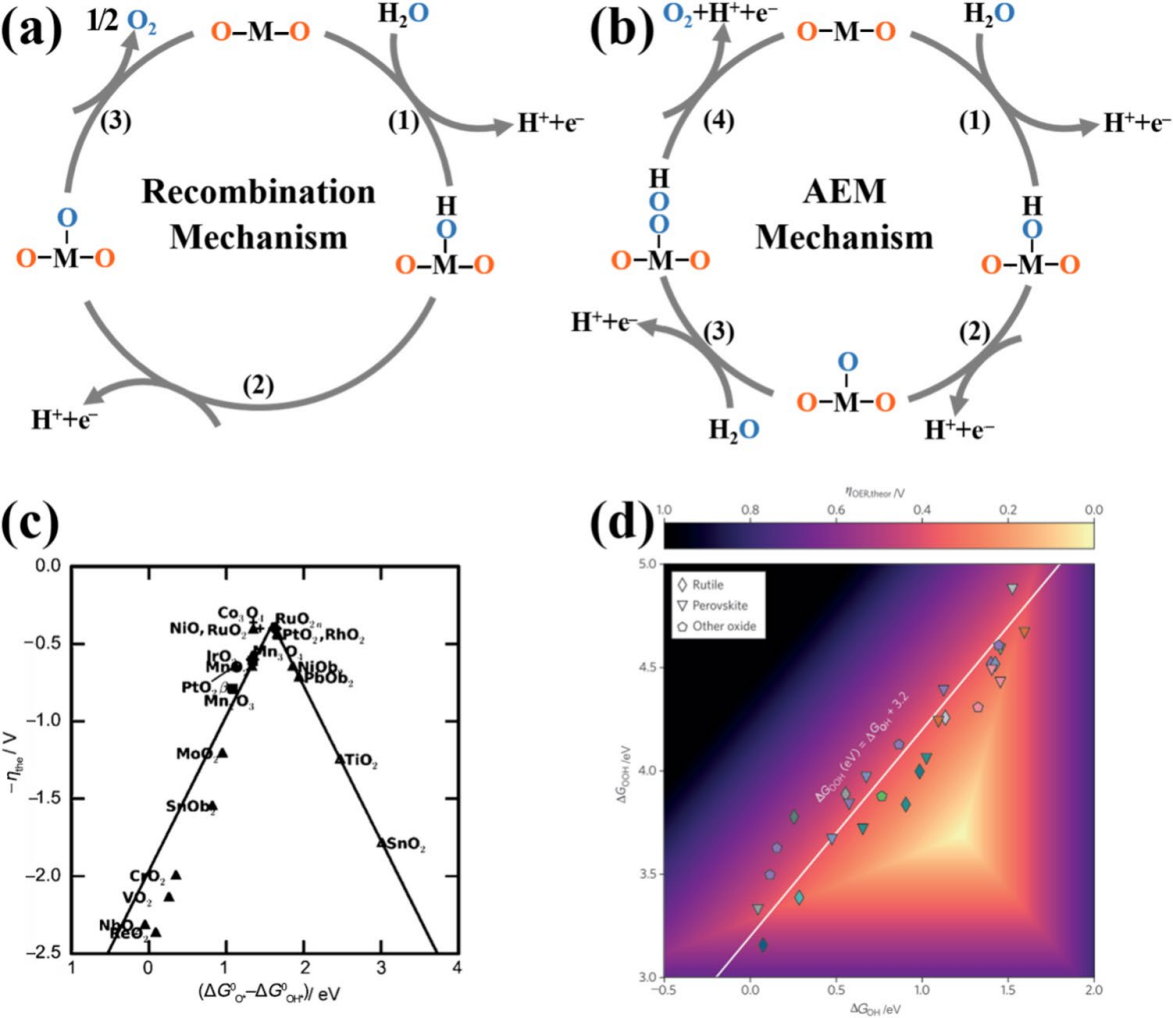
Schematic illustration of OER in acid. **a** Recombination mechanism based on kinetic studies. **b** AEM based on thermodynamic studies. Reprinted with permission from Ref. [43]. Copyright © 2015, Elsevier. **c** Scaling relationship between the binding energies of *OH and *OOH. Reprinted with permission from Ref. [47]. Copyright © 2011, Wiley-VCH. **d** Scaling relation between the binding energies of *OOH and *OH on various TMOs. Reprinted with permission from Ref. [48]. Copyright © 2016, Springer Nature

A variety of OER reaction mechanisms have been proposed in acidic media, and these have been consolidated in several reviews [40, 41]. Table 1 summarizes representative reaction mechanisms. Among these, the oxide pathway and the electrochemical oxide pathway are the most widely accepted mechanisms based on kinetic analyses (Fig. 2a) [40–43].

**Table 1:** Mechanistic models of OER from kinetic analysis [44]

Mechanism	Reaction pathways	References
Electrochemical oxide path	1) M + H_2_O → M–OH + H^+^ + e^−^ 2) M–OH → M–O + H^+^ + e^−^ 3) 2 M–O → 2 M + O_2_	[45]
Oxide path	1) M + H_2_O → M–OH + H^+^ + e^−^ 2) 2 M–OH → M–O + M + H_2_O 3) 2 M–O → 2 M + O_2_	[45]
DFT-predicted peroxide path	1) M + H_2_O → M–OH + H^+^ + e^−^ 2) 2 M–OH → M–O + H^+^ + e^−^ 3) M–O + H_2_O → M–OOH + H^+^ + e^−^ 4) M–OOH → M + O_2_ + H^+^ + e^−^	[46]
Electrochemical metal peroxide Path	1) M + H_2_O → M–OH + H^+^ + e^−^ 2) 2 M–OH → M–O + M + H_2_O 3) M–O + H_2_O → M–OOH + H^+^ + e^−^ 4) 2 M–OOH → M–O + H_2_O + M + O_2_	[40]

In both the oxide and electrochemical oxide pathways, the reaction mechanisms entail the formation of intermediate species (*OH and *O). In acidic electrolytes, the absence of free hydroxyl groups (HO^–^) precludes their adsorption onto active sites (M), necessitating an alternative mechanism for hydroxyl intermediate (*OH) formation. This process involves breaking strong covalent O–H bonds, which facilitates the subsequent water adsorption at the active site. The high energy barrier is also a reason for the slow reaction kinetics in acidic environments compared to basic environments [49]. The adsorption of OH^–^ at the active site of *OH formation results in the formation of intermediates involving the different energy states of M–OH_ADS_ and M–*OH_ADS_, which constitute different reaction mechanisms in the OER process [50, 51]. The electrochemical oxide pathway proceeds via electron transfer, while the oxide pathway involves recombination steps. The adsorption strength of intermediates determines the dominant pathway, with oxygen ultimately released through combination of intermediates. Regarding kinetics, the Tafel slope is a frequently employed parameter with the purpose of determining the rate of multi-electron transfer reactions and gaining insight into the underlying reaction mechanism [52]. However, the complex OER mechanism cannot be fully resolved through kinetic parameters alone, due to uncertainties in rate-determining steps, competing pathways, and experimental variables [40, 42]. Thus, complementary thermodynamic investigations are essential.

Thermodynamic analysis based on density functional theory (DFT) has been demonstrated to be an effective approach for elucidating the underlying mechanism of the OER. The conventional adsorbate evolution mechanism (AEM) has been widely acknowledged, which consists of four-electron transfer steps and three intermediates (*OH, *O, and *OOH) [46, 47, 53]. The acidic OER reaction process is depicted in Fig. 2b. First, the H_2_O molecule attacks the unsaturated metal catalytic sites, and H^+^ is removed to form the *OH species (Step 1). In a further deprotonation process, *O is formed from *OH (Step 2), and the O–O coupling is initiated by a nucleophilic attack of another H_2_O molecule, resulting in the formation of the *OOH intermediate (Step 3). At this final stage, the dissociation of the last proton results in the desorption of oxygen from the catalyst surface (Step 4). This process releases the active metal catalytic sites and allows for the completion of the catalytic reaction.

The AEM enables evaluation of intermediate binding energies through sequential steps, providing insights into reaction origins and thermodynamic pathways. Under equilibrium conditions, the overall reaction Gibbs free energy (Δ*G*) is calculated as 4.92 eV, with each elementary step exhibiting a distinct Δ*G*. The step with the highest Δ*G* is identified as the rate-limiting step (RLS), governing the overall OER kinetics. Δ*G*_2_ (*OH deprotonation) or Δ*G*_3_ (*OOH formation) is the predominant RLS, and therefore the catalyst activity can be predicted by numerical visualization of the differences in binding energy between the O and OH intermediates (Δ*G*_O_−Δ*G*_OH_) [47]. Following Sabatier's principle, electrocatalysts with high OER activity must exhibit an optimal oxygen binding energy [54]. As shown in the volcano plot (Fig. 2c) [47], IrO_2_ exhibits moderate adsorption strength, positioning it near the volcano apex. Consequently, extensive efforts focus on designing volcano-apex IBEs through thermodynamic optimization.

Catalyst activity can be enhanced by modulating oxygen binding strength via strategies such as adjusting the d-band center [55, 56], tuning e_g_ occupancy [57], and optimizing metal–oxygen hybridization [58]. For example, by adjusting the chemical environment of IrO_6_ through binding Ir single atoms to the oxide, the OER electrocatalytic activity can be improved due to optimized d/p-band centers, lengthened Ir–O bonds, and reduced energy barriers for absorption/desorption [59]. However, dynamic reconfiguration of IBEs under harsh acidic OER conditions has been rarely considered in current studies, and more investigation into the source of the activity mechanism is needed. In addition, AEM-guided electrocatalysts have difficulty further improving the activity to go beyond the theoretical overpotential of 370 mV due to the defined ratio between ΔG_*OOH_ and ΔG_*OH_ (Fig. 2d) [48].

Recently, numerous excellent electrocatalysts have been identified that exhibit an overpotential for the OER below 370 mV, such as orthorhombic perovskite SrIr_0.8_Zn_0.2_O_3_ [60]. Furthermore, dynamic surface restructuring under high potentials has been shown to correlate with enhanced activity [61, 62], leading to the proposal of the lattice oxygen participation mechanism (LOM). The LOM provides a theoretical framework for understanding kinetic surface evolution and guiding the design of high-activity IBEs.

### Lattice Oxygen Mechanism (LOM)

**Fig. 3:**
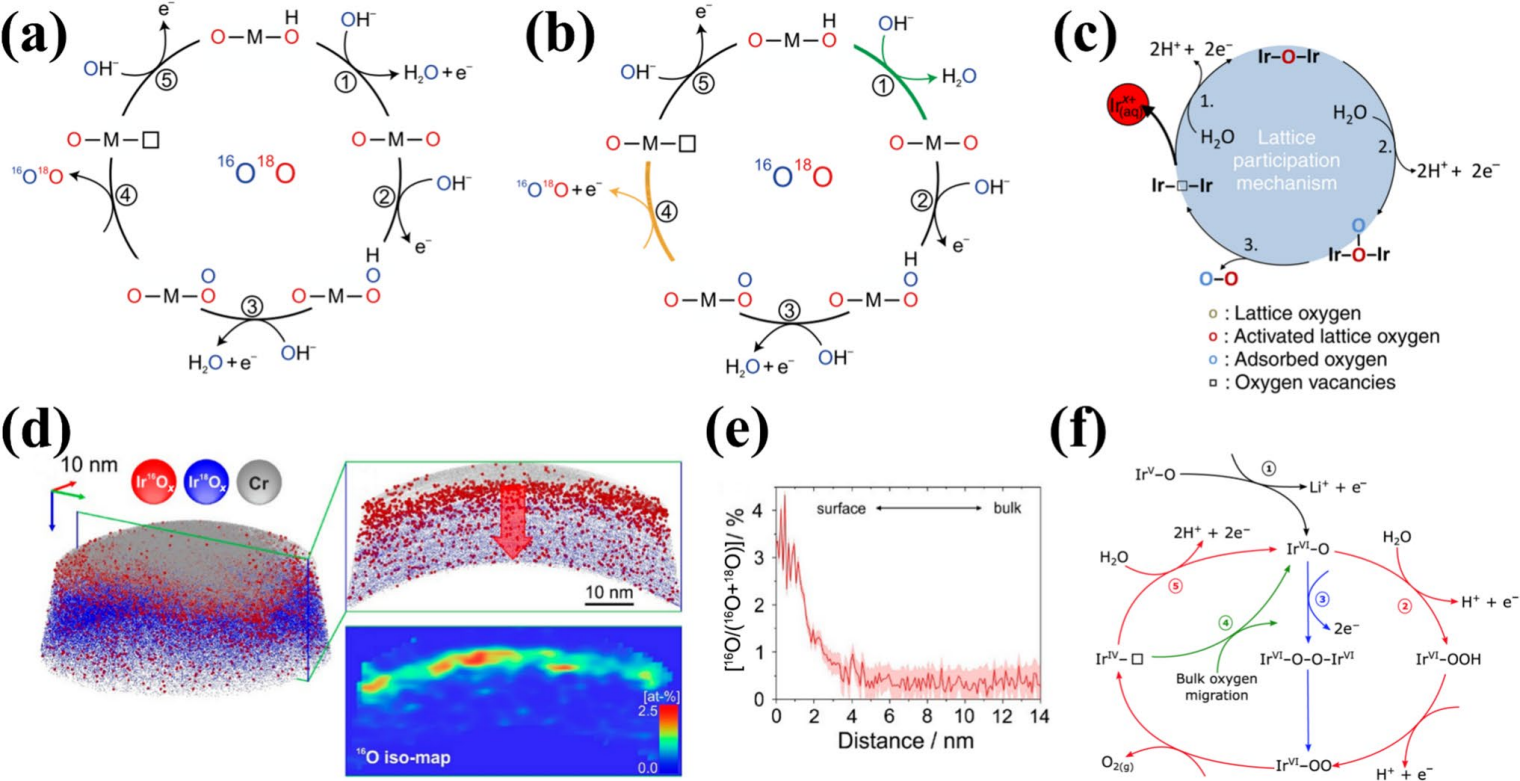
**a** Possible OER mechanisms involving concerted proton-electron transfer, and **b** uncoordinated proton-electron transfer on surface oxygen sites. Yellow is for the electron transfer step, green is for the proton transfer step, and charged intermediates are regulated by metal ion valence changes. Reprinted with permission from Ref. [71]. Copyright © 2017, Springer Nature. **c** Schematic diagram of a simplified OER mechanism. Reprinted with permission from Ref. [73]. Copyright © 2018, Springer Nature. **d** 3D APT reconstruction of a post-OER sample and the cross-sectional view of a 5-nm thick slice. **e** Depth profile showing ^16^O as a percentage of total oxygen. Reprinted with permission from Ref. [74]. Copyright © 2020, American Chemical Society. **f** Scheme representing the proposed OER mechanism on the surface of La_2_LiIrO_6_ in acid. Reprinted with permission from Ref. [75]. Copyright © 2016, Springer Nature

In contrast to the conventional AEM, the LOM circumvents scaling relationship limitations by directly involving lattice oxygen (O_L_) in the OER [63–66]. The catalytic surface in the LOM pathway is thermodynamically unstable, undergoing dynamic structural evolution during OER, including oxidation, exchange and release of lattice oxygen ligands, as well as the opening of the next cycle at the oxygen vacancies [67]. LOM activation requires specific electronic configurations of lattice oxygen and distinct redox mechanisms (e.g., single/double oxygen vacancy pathways), which depend on the number of metal active sites [68]. However, acidic LOM studies remain scarce due to poor acid stability observed in early LOM research [69, 70]. Thus, elucidating acidic LOM mechanisms often relies on extrapolating alkaline system insights. For example, Shao-Horn and colleagues employed in situ ^18^O isotopic labeling to investigate the lattice oxygen oxidation in different chalcogenides during OER [71]. In this process, lattice oxygen reacts with adsorbed oxygen species, forming an O–O bond that releases O_2_ while creating an oxygen vacancy. Concurrently, a potential OER mechanism for the proton–electron cooperative and non-cooperative transfer step at the surface oxygen site has been proposed (Fig. 3 a–b). Notably, LOM-capable oxides exhibit pH-dependent OER activity. Although the fundamental LOM pathway remains debated, Rong et al. [63] combined DFT calculations with experimental validation to demonstrate LOM in highly active catalysts, revealing reversible oxygen vacancy (O_V_) generation as a key feature.

While a unified mechanistic understanding of the LOM in acidic media remains elusive, it is plausible that LOM proceeds via analogous pathways in acid, based on experimental observations in alkaline systems [72]:

1 $$ {\mathrm{H}}_{{2}} {\mathrm{O}} + * \to *{\mathrm{OH}} + {\mathrm{H}}^{ + } + {\mathrm{e}}^{ - } $$

2 $$ *{\mathrm{OH}} \to *{\mathrm{O}} + {\mathrm{H}}^{ + } + {\mathrm{e}}^{ - } $$

3 $$ *{\mathrm{O}} + {\mathrm{O}}_{{\mathrm{L}}} \to {\mathrm{O}}_{{2}} + {\mathrm{O}}_{{\mathrm{V}}} $$

4 $$ {\mathrm{O}}_{{\mathrm{V}}} + {\text{ H}}_{{2}} {\mathrm{O}} \to *{\mathrm{OH}} + {\mathrm{H}}^{ + } + {\mathrm{e}}^{ - } $$

5 $$ *{\mathrm{H}} \to * + {\mathrm{H}}^{ + } + {\mathrm{e}}^{ - } $$

where, *, O_L_ and O_V_ represent the active site, lattice oxygen and surface-involved oxygen vacancies, respectively. The five fundamental stages of LOM encompass the attachment of the generated adsorbed *O species to the O_L_, the release of oxygen and O_V_ formation, the refilling of the O_V_ site, and the activation of catalytic cycling at the recleaned active site [64, 65, 72].

Concurrently, numerous researchers have expended considerable effort to enhance the comprehension of the LOM mechanism in acid. Cherevko et al. [73] combined nucleophilic attack and deprotonation into a single step, simplifying the reaction pathway (Fig. 3c). The LOM-dominated amorphous IrO_*x*_ is observed to display enhanced activities relative to other materials, attributed to the participation of activated O_L_ atoms. The absence of Ir–OOH formation enables the bypassing of scaling limitations. To date, the feasibility of the LOM pathway has been clearly validated with the development and combination with in situ technologies. For example, isotope labelling combined with atom probe tomography (APT), online electrochemical mass spectrometry (OLEMS) with ICP–MS to find that hydrous Ir_18_O_*x*_ lattice oxygen may be more likely to proceed LOM and accompany with Ir dissolution compared to rutile Ir_18_O_2_ [74]. Significantly, the O_L_ exchange of rutile IrO_2_ is mainly triggered in the initial 2.5 nm of the lattice (Fig. 3d–e), suggesting that the surface reorganization plays a pivotal role in influencing the intermediate stability and the degradation reaction pathway. A detailed understanding of surface oxygen exchange mechanisms is essential for optimizing catalyst stability. Tarascon et al. [75] proposed a modified OER mechanism in acid by studying of La_2_LiIrO_6_. Following lithium ion removal and Ir oxidation, surface O–O formation can be achieved by direct coupling of Ir^VI^–O species or water attack reactions, which finally leave O_V_ on the surface (Fig. 3f). Additional processes can also be employed, such as water dissociation or bulk O_L_ migration. The exceptional activity originates from electrophilic oxygen atoms acting as active sites at high potentials, but excessive cation/anion migration causes surface instability.

Compared to AEM, the LOM mechanism under acidic conditions can be summarized as follows: (i) LOM surpasses AEM in OER activity by bypassing scaling relationships; (ii) the presence of LOM is typically accompanied by severe metal dissolution and the formation of O_V_, which results in a reduction in the structure and OER stability; (iii) pH-dependent activity trends and isotopic labeling differentiate LOM from AEM; (iv) the AEM and LOM mechanisms involve a nucleophilic attack on the oxygen species, indicating a correlation between the electrophilic character of the oxygen and the observed activity. The difficulty of cations and anions redox reactions also determines the catalytic mechanism. Furthermore, the AEM and LOM differentially govern the activity and stability of Ir- and Ru-based catalysts. In AEM, OER proceeds via sequential proton-coupled electron transfer (PCET) steps involving surface-adsorbed intermediates (e.g., IrO_2_). Ru-based catalysts inherently favor LOM due to their ability to activate lattice oxygen, whereas Ir-based materials can be engineered to partially utilize LOM through defect engineering and doping strategies, balancing activity gains with dissolution resistance. Thus, the investigation of AEM and LOM with the objective of enhancing activity and stability represents a pivotal foundation for the exploration of efficient and stable catalysts.

### Mechanism of Different Ir Species and OER Degradation

**Fig. 4:**
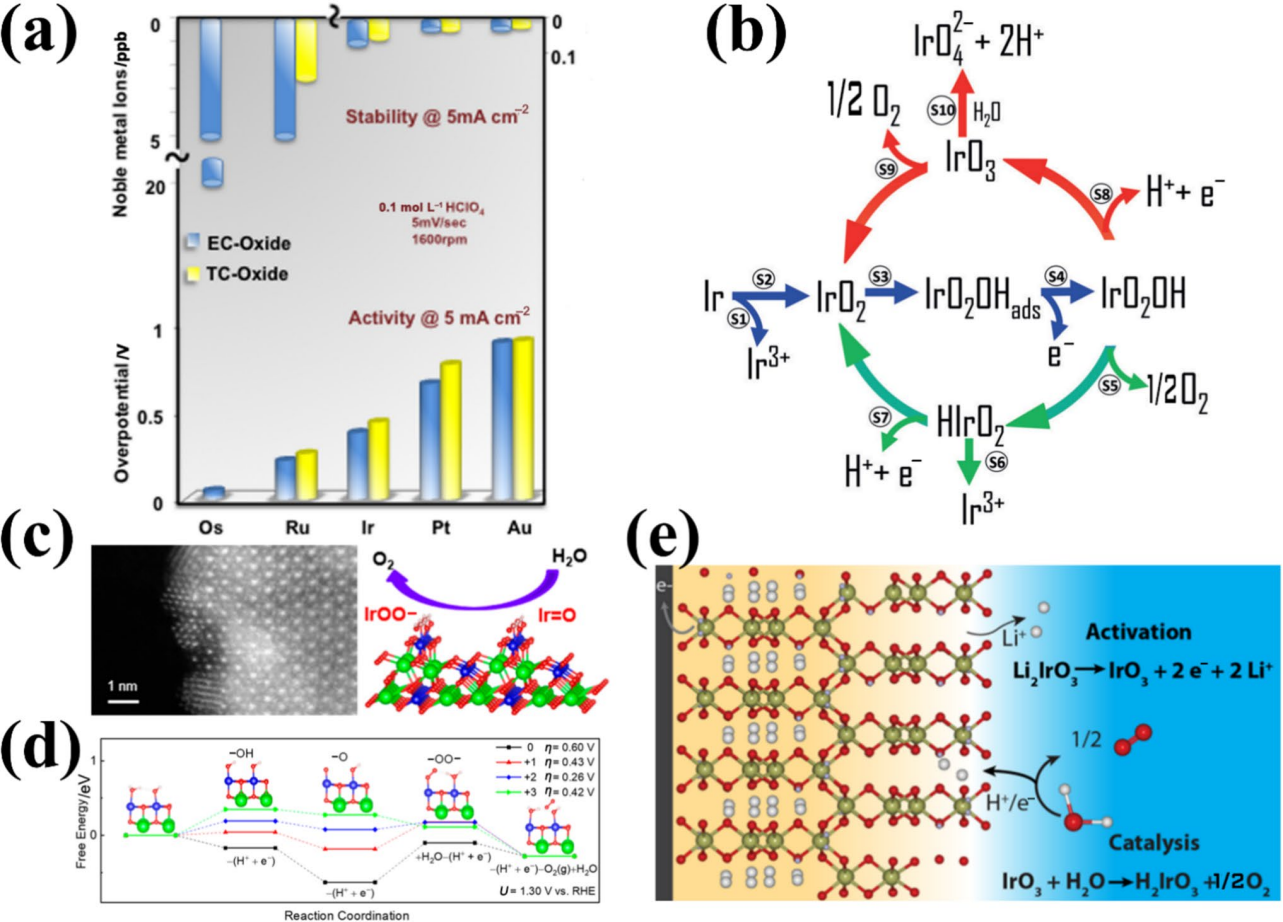
**a** Activity-stability trend for different metal oxide catalysts during the OER in acid. Reprinted with permission from Ref. [76]. Copyright © 2014, American Chemical Society. **b** Possible pathways for Ir dissolution during OER. Reprinted with permission from Ref. [78]. Copyright © 2018, Wiley-VCH. **c** Schematic representation of IrOO– and Ir = O intermediates stabilized on the Ir-rich surface layer. **d** Free energy diagrams of acidic OER with different net charge states on the surface of Ir-rich Ca_2_IrO_4_(110) at *U* = 1.30 V vs. RHE. Reprinted with permission from Ref. [85]. Copyright © 2022, American Chemical Society. **e** Schematic representation of the proton insertion process in Li_2_IrO_3_ and H_2_IrO_3_. Reprinted with permission from Ref. [86]. Copyright © 2019, American Chemical Society

The influence of metal dissolution on OER rates in acidic media reveals a direct relationship between the activity and stability of OER catalysts [36]. Studies on five distinct metal oxides have revealed that iridium oxides exhibit balanced activity and stability, making them optimal OER catalysts (Fig. 4a) [76]. However, rutile-IrO_2_ and its amorphous analogues show opposing activity-stability trends, necessitating mechanistic insights into OER intermediates and dissolution pathways to optimize IBEs design [77, 78]. Dissolution experiments on metal Ir and electrochemically grown hydrous Ir oxide suggest that Ir dissolution proceeds via Ir^III^ or Ir^VI^ intermediates [77]. The Ir dissolution pathway is summarized by monitoring Ir dissolution products from metallic Ir, reactively sputtered oxides and thermal oxides (Fig. 4b). In acidic media, the OER cycle commences with the discharge of water and the adsorption of *OH radicals on the catalyst surface, resulting in the formation of Ir^V^O_2_(OH) intermediates, regardless of the material. Subsequently, the value of the electrode potential determines the further path. At lower potentials, the Ir^V^O_2_(OH) intermediate decomposes to form HIr^III^O_2_ species, which can be further oxidized to IrO_2_ or dissolved to Ir^3+^. For the more active Ir catalysts (electrochemically formed Ir oxides, reactive sputtered IrO_2_) in this pathway, the intermediate dissolution kinetics is relatively fast, thus leading to a lower catalyst stability. At higher anodic potentials, the Ir^V^O_2_(OH) intermediate is further oxidized to the highly reactive IrO_3_, which can be transformed into IrO_2_ or reacted with water to form soluble IrO_4_^2−^. For the thermal formation of IrO_2_ under this pathway, there is a high amount of IrO_3_ and its low dissolution rate, so the decomposition kinetics is greater than the hydrolysis kinetics, which explains crystalline iridium oxide exhibiting excellent stability. Notably, metallic Ir undergoes unavoidable dissolution at high potentials even without OER, with dissolution extent dependent on its intrinsic properties [79–81]. Alexandrov et al. [82] further confirmed surface-bound iridium intermediates upon dissolution are more active based on solvent ab initio computational molecular dynamics (AIMD) simulations.

In addition to the above pathways, the dissolution mechanism is also associated with anionic redox processes. Savinova et al. [83] demonstrated the formation of electrophilic oxygen species (O^I−^) under reaction conditions using DFT, O K-edge spectra, and Ir 4f spectra. Compared to thermally formed Ir oxides, O^I−^ species are more active in electrochemical Ir oxides because of the interaction of two O^I−^ intermediates or O^I−^ and adsorbed water molecules at adjacent surface positions. Moreover, O^(II−*δ*)−^ species present on the surface of the catalysis can effectively balance with the adsorption free energy of oxygen intermediate (*OH, *OOH) and promote rapid O–O bond formation [84]. Thus, combined cationic/anionic mechanistic studies are critical for understanding degradation. To enhance stability, strategies to stabilize intermediates and suppress dissolution are essential. Yan et al. [85] found that two key intermediates (Ir^6+^ = O and Ir^6+^OO–) could be stabilized on the positively charged active sites of the Ir-rich surface layer by constructing Ca_2−*x*_IrO_4_ nanocrystals (Fig. 4c). The DFT calculations revealed that positive charges weaken electronegative group adsorption (Fig. 4d). When there are + 2 positive charges, –OO– becomes the dominant –OOH configuration under realistic OER conditions. Grimaud et al. [86] reported a strategy to stabilize IrO_3_ intermediates; H_2_IrO_3_ with three-dimensional proton intercalation/deintercalation channels was obtained by the isolation of β-IrO_3_ intermediates (Fig. 4e). H_2_IrO_3_ exhibits remarkable performances and stability due to its unique proton insertion capability. Therefore, the degradation of IBEs in OER conditions is primarily driven by oxidative dissolution, structural reconstruction, and particle agglomeration. Under high anodic potentials, Ir can undergo oxidation beyond its stable Ir^4+^ state, forming highly soluble Ir^3+^ and Ir^6+^ species. The dissolution process typically follows a dynamic equilibrium, where Ir^4+^ oxidizes to Ir^6+^ (IrO_3_ or IrO_4_-like species), which are highly soluble and prone to leaching into the electrolyte. Alternatively, under reducing conditions, Ir^6+^ can be reduced to Ir^3+^, which also exhibits significant solubility, further accelerating catalyst loss. This dissolution is strongly influenced by the reaction pathway (AEM vs. LOM), with LOM-involved catalysts showing increased lattice oxygen participation, which can destabilize the Ir–O framework and promote Ir dissolution. Additionally, surface reconstruction plays a key role, where amorphization and formation of highly disordered IrO_*x*_ species can either enhance durability by forming a passivating layer or facilitate dissolution by exposing undercoordinated sites. To mitigate these degradation pathways, the effect of intermediates on dissolution should be investigated in greater depth in future research into new OER materials to obtain high-activity and stability catalysts.

### Evaluation Metrics of Activity and Stability

**Fig. 5:**
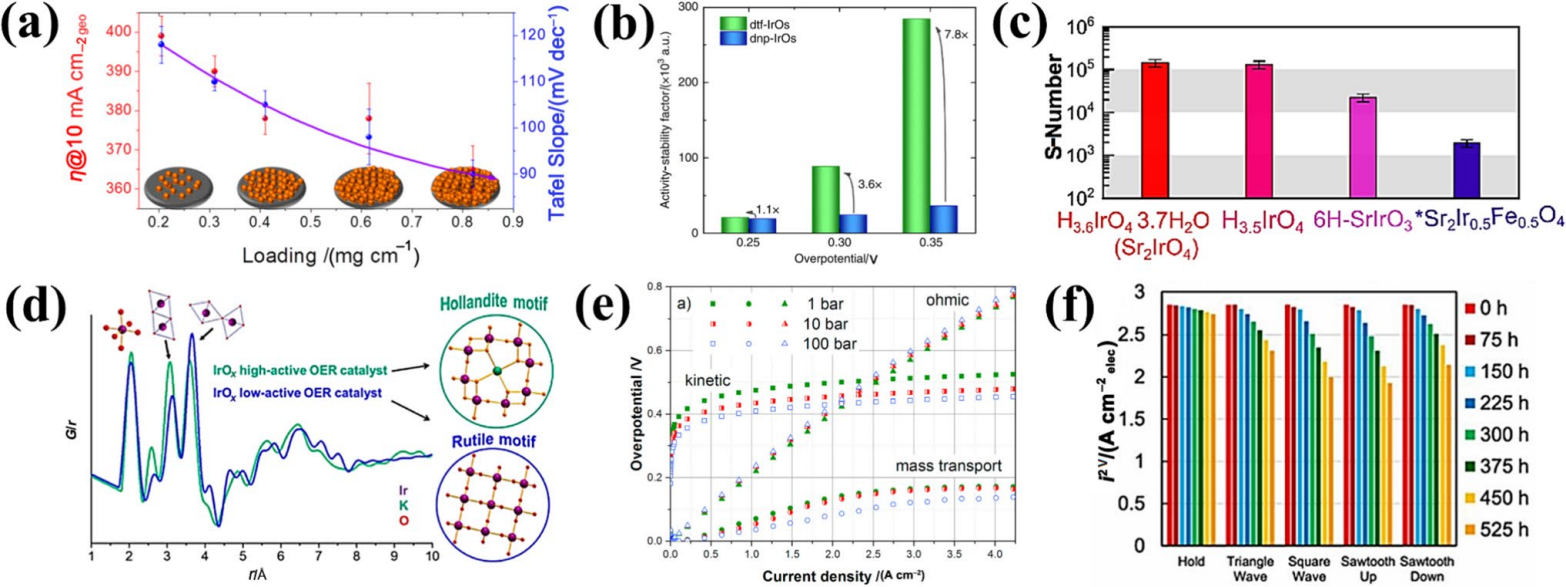
**a** Plot of *η*@10 mA cm^−2^ and the Tafel slope dependence on the catalyst’s loading mass. Reprinted with permission from Ref. [89]. Copyright © 2019, American Chemical Society. **b** Change in ASF values with the overpotential highlighting the importance of balancing activity-stability-conductivity properties of oxide materials for the OER. Reprinted with permission from Ref. [93]. Copyright © 2017, Springer Nature*.* **c** Calculated S-number for H_3.6_IrO_4_·3.7H_2_O compared to selected IBEs. Reprinted with permission from Ref. [94]. Copyright © 2020, American Chemical Society. **d** Schematic diagram of the structure-activity relationship based on IrO_*x*_ hydroxides; 1 Å = 1 × 10^−10^ m. Reprinted with permission from Ref. [95]. Copyright © 2017, American Chemical Society. **e** Overpotentials of different voltage losses in the PEMWE under different pressures; 1 bar = 100 kPa. Reprinted with permission from Ref. [96]. Copyright © 2016, Elsevier. **f** Different ASTs employed for evaluating durability tests of PEMWEs. Reprinted with permission from Ref. [100]. Copyright © 2019, The Authors

The establishment of a reliable evaluation metric that can accurately assess catalyst activity and stability is essential for assessing the OER activity of IBEs, particularly in complex acidic conditions [87, 88]. The evaluation of OER activity is commonly based on key performance metrics, including the overpotential (*η*@10 mA cm^−2^), Tafel slope, mass activity, and turnover frequency (TOF). The overpotential, defined as the extra potential required beyond the thermodynamic OER potential (1.23 V vs. RHE), is frequently measured at a current density of 10 mA cm^−2^, which approximates the operational conditions of solar-driven water splitting. The Tafel slope, which quantifies the rate of potential increase per logarithmic increase in current, provides insight into reaction kinetics, with lower values indicating more efficient charge transfer. However, there is an issue to be addressed that the *η*@10 mA cm^−2^ and Tafel slope can fluctuate in response to variations in the catalyst loading (Fig. 5a) [89], thereby rendering the assessment of the intrinsic activity of the catalyst using these two metrics less precise. Additionally, mass activity (A mg^−1^), defined as the current per unit mass of catalyst, is crucial for evaluating the utilization efficiency of Ir, particularly given the cost constraints of noble metal catalysts. Electrochemical active surface area (ECSA)-normalized ratio activity and TOF can be used as relatively more reliable activity evaluation metrics and do not vary with catalyst loading. The intrinsic activity parameter TOF can be derived from the current density at a fixed potential and the metal sites that are actually involved, which can be described as follows [90]:

6 $${\mathrm{TOF}} = j \cdot N_{A} /n \cdot F \cdot \varGamma $$

where, *j*, *N*_A_, and *F* respectively represent the current density, Avogadro number, and Faraday constant; the symbols *n* and *Γ* denote the number of electrons transferred during the evolution of a single O_2_ molecule and the surface concentration or number of active sites, respectively.

Notably, the TOF can more accurately reflect the intrinsic catalytic activity of an electrocatalyst only when active sites are fully exposed without coverage. While electrocatalytic current density is a key performance metric, its accuracy is compromised by non-Faradaic side reactions. Thus, adopting rational and standardized evaluation criteria is critical. To quantify OER-specific current contributions and precisely measure onset potentials and Faradaic efficiency (FE), Wen et al. [91] used online chip-based electrochemical mass spectrometry (chip EC-MS) to investigate the OER process.

For durability evaluation metrics, judging the value of the overpotential increment by the chronopotentiometry method (CP) is widely used, but differences in working potential will reduce the correlation between CP results and catalyst durability. The chronoamperometric (CA) method is more reasonable for assessing current density retention at the end by applying a constant potential, but *iR*-compensation should be used to correct the applied potential [92]. To overcome the limitations of the dissolution of studied materials and prevent complicating the stability assessment, S-number and the activity-stability factors (ASFs) can be used as reasonable indicators of durability due to their independence from catalyst loading and ECSA [73, 93]. The S-number is a ratio that represents the relationship between the number of oxygen molecules produced and the number of dissolved Ir atoms. This ratio remains constant regardless of the active site involved. The ASF is defined as the ratio between the rate of oxygen production (*j*) and the rate of metal dissolution (*s*). The ratio is only comparable when the potential is constant (*η*), and can be expressed as the following equations [73, 93]:

7 $$ {\text{S - number }} = \frac{{{\mathrm{n}}_{{{\mathrm{O2(OER)}}}} }}{{{\mathrm{n}}_{{{\mathrm{Ir(dissolved)}}}} }} $$

8 $$ {\text{ASF }}\left( \eta \right) \, = \frac{{j - {{s}}}}{{{s}}} $$

The S-number is in principle the same as the ASF; the higher the ASF and S values, the superior the stability of the electrocatalyst. It is crucial to acknowledge that in ASF and S numbers, the dissolution of Ir is regarded as the sole factor in the activity degradation. However, other factors can also contribute to the decline of the electrocatalyst, such as agglomeration and structural changes during OER. The inherent conductivity of the oxide material also affects the ASF value (Fig. 5b). Grimaud et al. [94] reported that the protonated phase of the layered junction H_3.6_IrO_4_·3.7H_2_O of the S-number was ∼10^5^, lower than that of rutile-IrO_2_ at ∼10^6^, which was more active although less stable than rutile-IrO_2_ (Fig. 5c). Furthermore, Willinger et al. [95] have demonstrated that the loss of efficacy associated with rutile-IrO_2_ can be attributed to a rapid structural collapse resulting from the ratio between corner- and edge-sharing IrO_6_ octahedra (Fig. 5d). Thus, integrating advanced characterization techniques with electrochemical analysis is critical for accurately assessing catalyst activity and stability, elucidating structure-function relationships, and unraveling degradation mechanisms.

Comparative performance assessments between three-electrode systems and PEMWE cell tests under industrial operating conditions reveal that PEMWE tests offer more realistic insights into electrocatalyst activity and stability. For activity evaluation, the voltage-current density relationship serves as a fundamental metric to assess PEMWE conversion efficiency. The voltage of PEMWE cells is determined by thermoneutral cell voltage *U*_tn_ in combination with three primary overpotentials (kinetic *η*_kinetic_, ohmic *η*_Ω_, and mass transport *η*_mt_). These overpotentials are dependent on current density and pressure (Fig. 5e) [96, 97], as described by Eqs. (9, 10):

9 $$ U_{{{\mathrm{cell}}}} = U_{{{\mathrm{tn}}}} + \eta_{{{\mathrm{kinetic}}}} + \eta_{\Omega } + \eta_{{{\mathrm{mt}}}} $$

10 $$ U_{{{\mathrm{tn}}}} = { 1}.{48}0\;{ 8} - 0.000\;{ 845\; 6 }\left( {T - {298}.{15}} \right) $$

where *T* is cell operating temperature (K). The details of the calculations are referred to M. Suermann's article [96]. The *η*_kinetic_ of the cell is dominated by the anodic kinetic overpotential due to the fast kinetics of the cathodic HER. The *η*_Ω_ encompasses several different resistances, including those associated with the ionic conductivity of the membrane, catalyst layers (CLs) resistance, and interfacial resistance between PEMWE components. Notably, the latter is influenced by the passivation of the porous diffusion layer during prolonged exposure to high voltage conditions. The *η*_mt_ is caused by the liquid/ion transport resistance between the catalyst layer and the gas diffusion layer (GDL), which is mainly hindered by the accumulation of gas bubbles at the CLs and GDLs. Electrochemical impedance spectroscopy (EIS) measurements at different current densities can be used to resolve changes in activities caused by kinetic, ohmic and mass transport effects [98]. In addition, the energy efficiency (*ε*) can be used to assess how efficient the PEMWE is in the energy conversion and utilization process by Eq. (11) [97]:

11 $$ \varepsilon = \, \left( {U_{{{\mathrm{tn}}}} /U_{{{\mathrm{cell}}}} } \right) \times {1}00\% $$

The lowest energy consumption required to produce hydrogen is 39.4 kWh kg^−1^. This is when the PEMWE cell is operated at *U*_tn_ of 1.48 V at 25 °C, and represents a lower bound of the projected cost of commercial renewable energy, which is US$0.026 kWh^−1^. To minimize energy consumption, the *ε* needs to be as high as possible for a given current density. To illustrate, the performance of one of the most efficient PEMWEs currently reported has a cell voltage of 1.6 V (*ε* = 89.6%) at 1.5 A cm^−2^ with an energy consumption of approximately 44.0 kWh per kg of H_2_, which corresponds to an energy cost of US$1.14 per kg of H_2_ [99]. However, it is still far from the ideal PEMWE operation targets adopted by the DOE of 3.0 A cm^−2^@1.6 V, US$1 per kg of H_2_, and 46.0 kWh kg^−1^ of H_2_.

In assessing durability, a constant-current hold was applied, with the aim of evaluating both the short- and long-term effects of degradation under constant operation at a current density greater than 1 A cm^−2^. The voltage losses were expressed in µV h^−1^ or mV h^−1^. However, the current catalyst lifetimes are considerably shorter than those of commercial PEMWEs (> 50 000 h), and there is no consensus on standard test protocols for durability metrics, especially under dynamic operating conditions (Fig. 5f) [100]. Therefore, a fine-tuned accelerated stress test (AST) protocol was used to evaluate the catalyst layer life to obtain reliable degradation metrics on shorter time scales [101]. Incorporating a comprehensive discussion on mass transport effects, ohmic losses, and interfacial resistance within a complete PEMWE system would significantly enhance the industrial relevance. Mass transport limitations, particularly those associated with reactant diffusion and the removal of gaseous products, can substantially affect the overall efficiency and long-term stability of the electrolyzer. Addressing strategies to mitigate these challenges would provide valuable insights into improving system performance, including optimizing electrode porosity, implementing advanced flow-field designs, and enhancing GDLs. Similarly, ohmic losses, arising from membrane resistance, contact resistance, and ionic conductivity, directly influence cell voltage and energy efficiency. A quantitative analysis of these losses, supported by EIS or in situ resistance measurements, would offer a more comprehensive evaluation of catalyst performance under realistic operational conditions. Furthermore, interfacial resistance at the catalyst-electrode and electrode-membrane interfaces plays a crucial role in determining charge transfer kinetics and catalyst utilization. Investigating surface modifications, catalyst-support interactions, and the distribution of ionomers could reveal key strategies for minimizing interfacial losses and improving overall efficiency. A systematic evaluation of these factors within a full-cell configuration would enhance the industrial applicability, offering critical insights for the development of more efficient, stable, and commercially viable OER materials.

## Strategies to Boost Activity of IBEs

Despite profound understanding of the reaction mechanism for OER, multiple reaction pathways may occur simultaneously during OER. This complexity poses a significant challenge in categorizing IBEs by their specific reaction mechanisms [102, 103]. We first review the performance of current advanced IBEs in Table 2, and summarize the impact of variations in key parameters on OER activity in Table 3. The activity is one of the most important considerations in the design of IBEs; the number and activity of active sites will directly determine the level of catalyst activities [104]. We summarize the activity strategies used in state-of-the-art IBEs to provide a reference for the rational design. Among the strategies to boost the activity are the design of morphology/size, optimization of structure, and modulation of the electronic configuration. It is worth noting that these strategies are not limited to influencing activity alone but can also be a combination of strategies acting together to improve activities.

**Table 2:** Activities of the current advanced IBEs for the discussed electrocatalysts in acidic media

Catalyst	Electrolyte	Overpotential/ (mV @ 10 mA cm^−2^)	TOF/s^−1^	Tafel slope/ (mV dec^−1^)	References
Ir-Te nanowires/C	0.5 mol L^−1^ H_2_SO_4_	284	–	66.3	[105]
IrO_*x*_ nanorods/Sb-SnO_2_	0.1 mol L^−1^ HClO_4_	240	–	42	[106]
Ir-IrO_*x*_/C-20	0.5 mol L^−1^ H_2_SO_4_	198	0.177	106.3	[84]
RuIr nanosized-coral	0.5 mol L^−1^ H_2_SO_4_	165	–	–	[107]
Dealloyed nanoporous IrNi	0.5 mol L^−1^ H_2_SO_4_	248	–	38	[108]
RuIrO_*x*_	0.5 mol L^−1^ H_2_SO_4_	233	–	42	[109]
IrO_2_-400	0.1 mol L^−1^ HClO_4_	~ 300	–	50	[110]
h-HL-Ir SACs	0.1 mol L^−1^ HClO_4_	216	4.17	43	[111]
Ir-COP	0.5 mol L^−1^ H_2_SO_4_	242	1.43	41	[112]
IrO_*x*_	0.5 mol L^−1^ H_2_SO_4_	231	–	48	[113]
amorphous Ir NSs	0.1 mol L^−1^ HClO_4_	255	0.16	40	[114]
Li-IrO_*x*_	0.5 mol L^−1^ H_2_SO_4_	290	0.3	39	[115]
IrCuNi deeply concave nanocubes	0.1 mol L^−1^ HClO_4_	273	–	41	[116]
ZnNiCoIrMn	0.1 mol L^−1^ HClO_4_	237	7.53	46	[117]
IrO_*x*_/9R-BaIrO_3_	0.5 mol L^−1^ H_2_SO_4_	230	–	80	[118]
Sr_2_CaIrO_6_	0.1 mol L^−1^ HClO_4_	250	0.71	33	[119]
Pr_2_Ir_2_O_7_	0.1 mol L^−1^ HClO_4_	290	–	–	[120]
Y_2_Ru_1.2_Ir_0.8_O_7_	0.5 mol L^−1^ H_2_SO_4_	220	–	47.56	[121]
Ir-NiCo_2_O_4_ nanosheets	0.5 mol L^−1^ H_2_SO_4_	240	1.13	60	[122]
Ir@Sr-p-TiO_2_ nanowires	0.5 mol L^−1^ H_2_SO_4_	250	0.68	51.6	[123]
3R-IrO_2_	0.1 mol L^−1^ HClO_4_	188	5.7	52	[124]
e–H-Na-213	0.1 mol L^−1^ HClO_4_	270	–	46.3	[125]
IrHf_*x*_O_*y*_	0.1 mol L^−1^ HClO_4_	300	4.04	50	[126]
Ti-IrO_*x*_/Ir	0.5 mol L^−1^ H_2_SO_4_	254	–	48	[127]
Sr-IrMnO_2_/CNTs	0.5 mol L^−1^ H_2_SO_4_	236	2.1	55.6	[128]
PdCu/Ir/C	0.1 mol L^−1^ HClO_4_	283	–	59.6	[129]
IrCo@IrO_*x*_-3L	0.05 mol L^−1^ H_2_SO_4_	247	–	49	[130]
Tensile-strained Ir/MnO_2_	0.1 mol L^−1^ HClO_4_	198	0.53	56.6	[131]
Ta_0.1_Tm_0.1_Ir_0.8_O_2−*δ*_ with grain boundaries	0.5 mol L^−1^ H_2_SO_4_	198	2.54	64	[132]

Data are extracted from different resources, thus with different significant digits

**Table 3:** The impact of changes in the Tafel slope, overpotential, and TOF on OER activity

Parameter	Impact on OER performance	Ideal trend for high performance
Overpotential (*η*)	Lower *η* reduces energy consumption and improves efficiency	Lower is better (e.g., *η*@10 mA cm^−2^ < 300 mV)
Tafel slope	Lower slope indicates faster reaction kinetics and efficient charge transfer	Lower is better (e.g., < 40 mV dec^−1^)
Turnover frequency (TOF)	Higher TOF suggests more efficient active site utilization	Higher is better (e.g., > 0.1 s^−1^)
Combined effect	Low *η* + Low Tafel + High TOF → Optimal OER performance	Balanced optimization is key

### Design of Morphology/Size

#### Morphology Manipulation

The structures and morphologies of the material are highly dependent on the atomic arrangements and electronic structures at the surface [22, 133]. Strategically tailoring the morphology and size of the material can enhance the exposure of active sites and minimize the utilization of Ir, effectively regulate the adsorption/desorption kinetics of the reaction intermediates and reactants, thereby enhancing the activity of the OER.

**Fig. 6:**
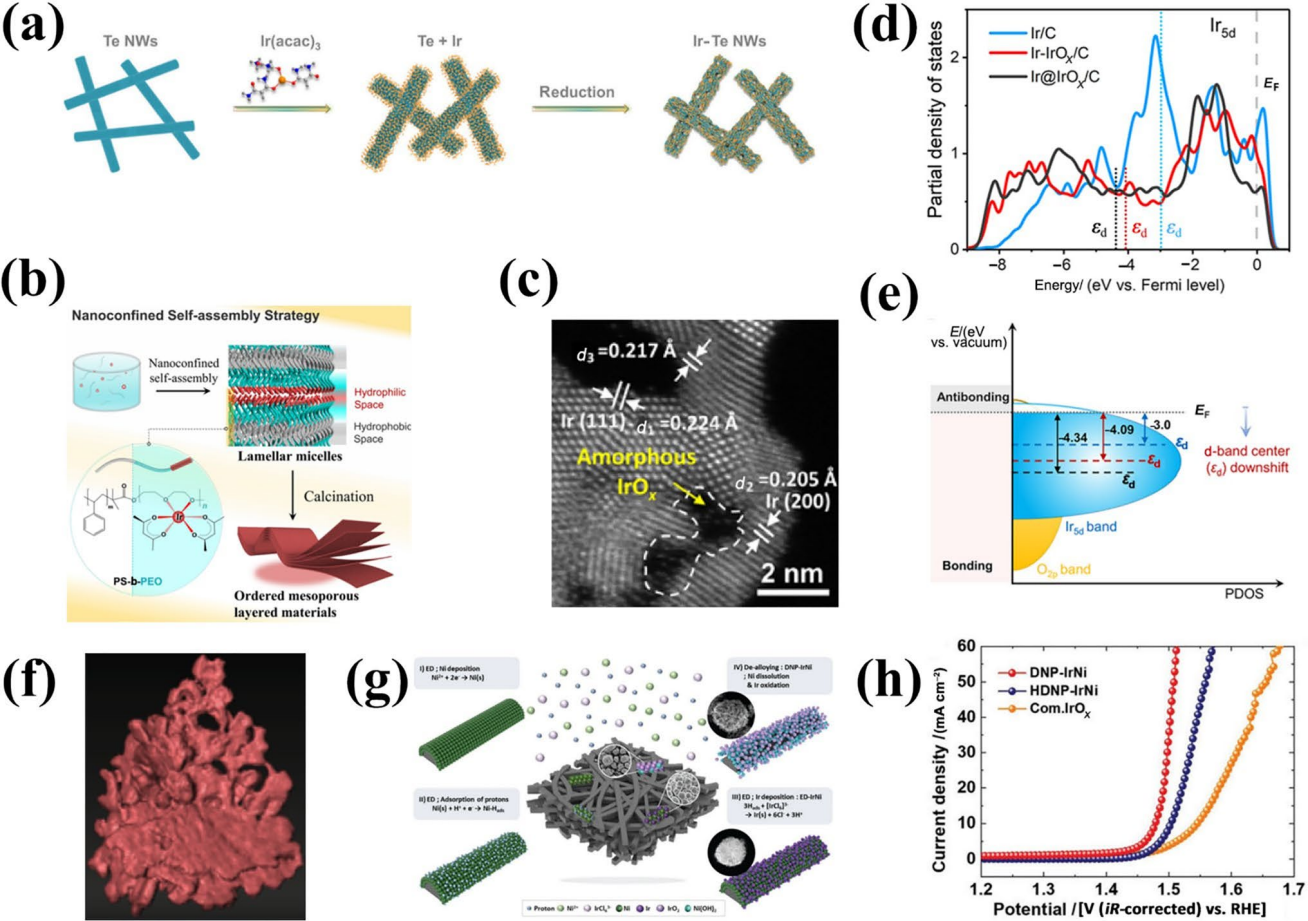
**a** Schematic illustration of the synthesis of 1D porous Ir-Te NWs. Reprinted with permission from Ref. [105]. Copyright © 2021, Springer Nature. **b** Illustration of the formation of ordered mesoporous lamellar Ir-IrO_*x*_/C via the nano-confined self-assembly approach. **c** High-resolution AC-HAADF-STEM image of the Ir-IrO_*x*_/C-20. **d** Partial density of state (PDOS) calculations, and **e** d-band center (*ε*_d_) of Ir-IrO_*x*_/C. Reprinted with permission from Ref. [84]. Copyright © 2022, American Chemical Society. **f** 3D tomographic reconstruction of RuIr-NC. Reprinted with permission from Ref. [107]. Copyright © 2021, Springer Nature. **g** Schematic illustration of 3D dealloyed nanoporous IrNi (DNP-IrNi). **h** OER polarization curves of DNP-IrNi. Reprinted with permission from Ref. [108]. Copyright © 2022, The Royal Society of Chemistry

One-dimensional nanowires (1D-NWs) are frequently observed to possess distinctive structural and physicochemical properties [134, 135]. For instance, metal 1D-NWs have been demonstrated to exhibit exceptional electrical conductivity, a degree of surface coordination that is not yet fully occupied, and an elevated specific surface area, all of which render them as potential contenders for high-performance electrocatalysts [136, 137]. Nevertheless, it is crucial to regulate the growth of 1D IBEs, as they exhibit a homogeneous nucleation of Ir accompanied by a high reduction potential and a low energy barrier. Huang et al. [105] used Te nanowires as a template to assist the synthesis of Ir-Te 1D-NWs (Fig. 6a), and they exhibit highly enhanced OER performance with *η*@10 mA cm^−2^ of 284 mV and a smaller Tafel slope implying faster reaction kinetics under acidic conditions, which is attributed to the larger electrochemical surface area and a lower resistance. In addition, self-supporting 1D nanostructures consisting of ultra-small nanoparticles have anisotropic properties that facilitate electron transfer, which can effectively reduce dissolution, aggregation and Ostwald ripening during OER [138]. The construction of 1D structures via particle aggregation of Ir can reduce the surface energy of specific low-index crystalline surfaces to preferentially expose them and enhance the strong electronic effects among nanoparticles, thus optimizing the adsorption energy of intermediates on the catalyst [139, 140]. Kakinuma et al. [106] loaded 1D IrO_*x*_ nanorods (IrO_*x*_ NR) on Sb-doped SnO_2_, which showed 10 times more mass activities than commercial IrO_*x*_. Benefiting from the geometry of IrO_*x*_ NR, increasing the number of active sites and the terminal oxygen (O_t_) neighboring on the surface drives the adsorbed O_t_ closer to the Ir sites. Notably, in the process of simulation of real activity-morphology trends, the effects of 1D nanostructure features on properties such as reaction product removal and the controllability and reproducibility of the synthesis process still need to be understood in depth.

In comparison to 1D nanostructures, two-dimensional (2D) layered catalysts exhibit a distinctive electronic structure and a tunable interlayer space, which facilitate the generation of more active species and enhance the accessibility of active sites [141–143]. Specifically, such ultrathin 2D layered structures with numerous unsaturated coordination centers are highly useful in stabilizing key intermediates and promoting OER [144]. Zhao et al. [84] employed a nano-constrained self-assembly strategy to synthesize Ir-IrO_*x*_/C nanosheets with ordered interlayer spaces, involving stable layered micelles (Fig. 6b). A highly ordered interlayer of mesoporous nanochannels is observed within the nanosheets, which are also found to contain Ir-IrO_*x*_ nanoparticles distributed uniformly (Fig. 6c). The unique structural arrangement observed in this material has been shown to enhance both the number of active sites and the efficiency of mass transfer for water oxidation. In addition, the distinctive oxygen coordination environment (O^(II–*δ*)–^) on the catalyst's surface stimulates the nucleophilic attack of water molecules and expedites the formation of O–O bonds. The downshifted d-band center (*ε*_d_) derived from the partial density of states (PDOS) indicates that the adsorption of oxygen species has been weakened, resulting in a shorter Ir–O^(II–*δ*)–^ bond (Fig. 6d–e). This phenomenon allows a valid modulation of the interaction with oxygen intermediates and enhances OER activity. Synthesizing 2D materials with high controllability and desired structural features remains one of the greatest challenges. Kitagawa et al. [107] constructed a Ru-Ir catalyst with a unique lamellar coral-like structure (RuIr-NC) and 3 nm-thick extended (0001) facets by fine-tuning of the morphology (Fig. 6f). Compared to the spherical RuIr catalyst, RuIr-NC exhibits an excellent *η*@10 mA cm^−2^ of 165 mV. Although these catalysts have low *η*@10 mA cm^−2^ values, the degradation of activity due to stacking, aggregation and exfoliation of 2D materials in high current density applications needs to be addressed.

Most of the reported catalysts use powder-based catalysts to fabricate electrodes. However, the incorporation of polymer binders inevitably reduces conductivity, blocking active sites, and diminishes electrocatalytic efficiency [145, 146]. Designing three-dimensional (3D) structures within the catalyst layer represents a pivotal strategy to enhance overall performance. The integration of additional accessible electrochemical active sites and enhanced electron/mass transport capabilities optimizes the catalyst layer's functionality. Soo-Kil Kim et al. [108] prepared a self-supported structure featuring 3D spore-like IrNi electrocatalysts (DNP-IrNi) via co-electrodeposition and H-induced adsorption (Fig. 6g). The catalyst surface was reconstructed through continuous dealloying of nanostructures, enabling DNP-IrNi to achieve an impressively low *η*@10 mA cm^−2^ of 248 mV in acid (Fig. 6h), accompanied by a Tafel slope of 38 mV dec^−1^. The high activity and structural relationship of the 3D DNP-IrNi are attributed to enhanced binding affinity for oxygen-containing intermediates and resistance to coarsening. Open 3D nanostructures facilitate higher atomic utilization, active site exposure and substrate molecular diffusion, offering significant advantages. Li et al. [109] synthesized open nanocage structures (RuIrO_*x*_) with surface active sites accessible in three dimensions, significantly improving Ir atomic utilization. Moreover, this uniquely structured catalyst exhibits excellent performance. Zhu et al. [147] fabricated Ir-based cubic nanocages (≈1.1 nm walls) via Ir deposition on Pd nanocubes followed by partial core etching. The hollow structure and Ir-to-IrO_*x*_ active site conversion enhanced catalytic activity, achieving an overpotential of 226 mV at 10 mA cm^−2^(geo) in 0.1 mol L^−1^ HClO_4_, outperforming commercial Ir/C (300 mV).

Hence, strategically engineering specific morphological characteristics of IBEs (e.g., size, facets, porosity) is crucial to improving catalytic performance. This strategy should encompass the following objectives: (i) promoting efficient transport of reactants and products across the surface of the electrocatalyst, which limits the concentration of degradation; (ii) maximizing the accessibility of the exposed active sites to further enhance the catalytic activity; and (iii) ensuring active site integrity to resist corrosion under high currents, thereby extending operational lifespan.

#### Size Controlling

**Fig. 7:**
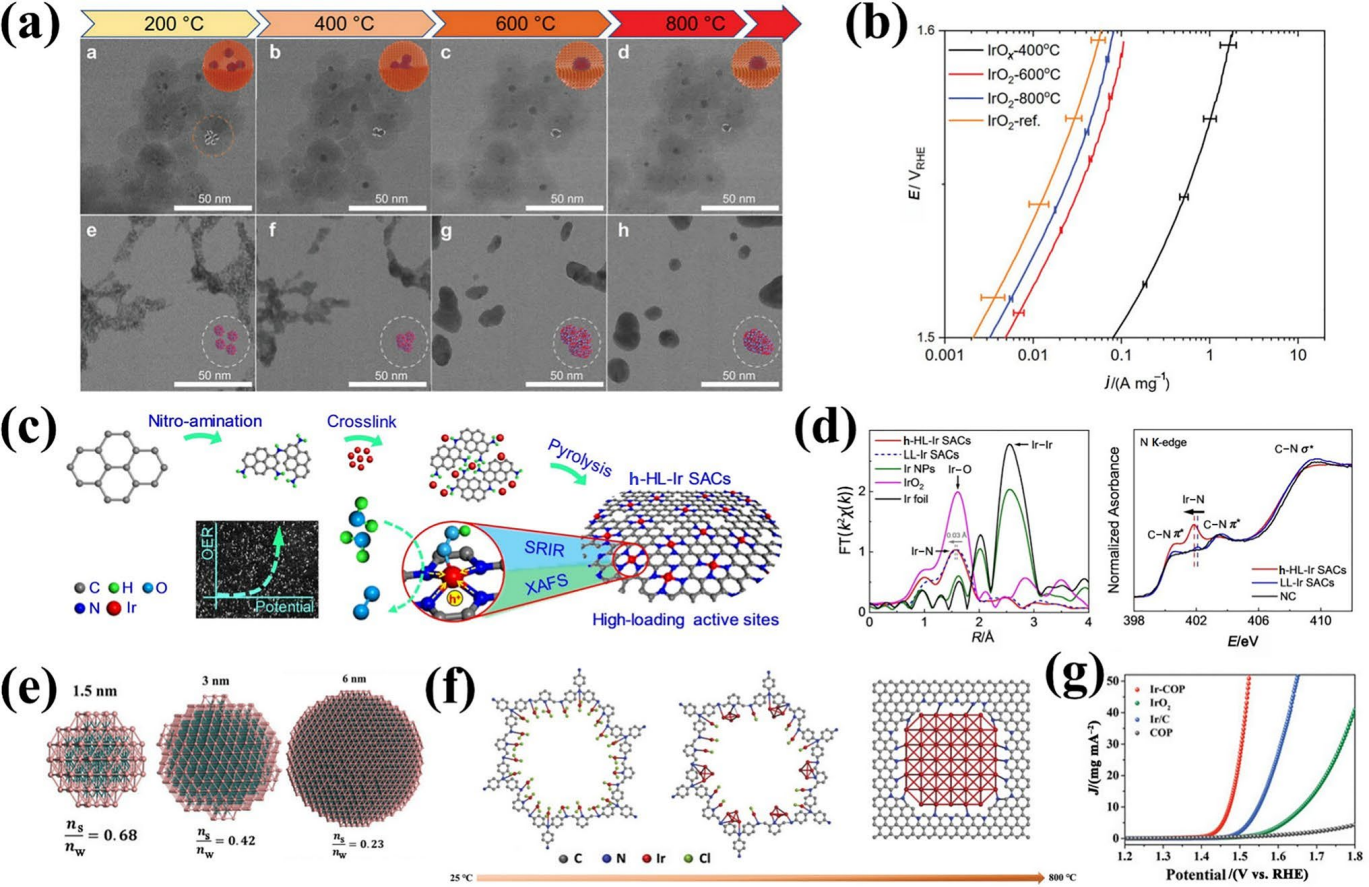
**a** Structural evolution comparison between IrO_*x*_@SiO_2_ and bare IrO_2_ nanoparticles during the in situ heating STEM experiment, and **b** mass normalized activities obtained from LSV. Reprinted with permission from Ref. [110]. Copyright © 2023, Wiley-VCH. **c** Scheme of the synthetic process of h-HL-Ir SACs. **d** The Ir L_3_-edge EXAFS and N K-edge XANES spectra of h-HL-Ir SACs. Reprinted with permission from Ref. [111]. Copyright © 2023, Wiley‐VCH. **e** Atom utilization study of Ir NPs with different particle sizes (*n*_s_—the number of Ir surface atoms, *n*_w_—the number of whole Ir atoms). **f** Proposed mechanism study of synthesizing Ir-COP catalysts. **g** LSV curves of Ir-COP in 0.5 mol L^−1^ H_2_SO_4_. Reprinted with permission from Ref. [112]. Copyright © 2023, Wiley-VCH

Variations in the sizes of metals and oxides (e.g., nanoparticles, nanoclusters, single atoms) can significantly modulate the electronic structure of the catalyst, influencing the binding strength of adsorbed intermediates [148–150]. Controlling IBEs sizes can increase the surface area, providing more active sites for OER. However, uncontrolled particle growth and morphological changes often occur during high-temperature calcination, leading to a decrease in surface area. Hence, investigating the relationship between particle size and catalytic activity of IBEs is critical for optimizing particle size in catalytic processes. Ledendecker et al. [110] used silica as a hard template to achieve precise control of Ir oxide particle sizes during high-temperature treatment, effectively avoiding surface area reduction and particle morphology changes (Fig. 7a). Moreover, the OER mass activity of IrO_2_ treated at 800 °C was 1.65 times that of commercial IrO_2_ at 1.5 V (Fig. 7b), further demonstrating the potential of size-controlled IBE nanoparticles in heterogeneous catalysis. Single-atom catalysts (SACs) with adjustable coordination environments, site positions, and electronic structures are considered efficient and cost-effective acid catalysts [151–153]. Enhancing the density of metal active sites in SACs and modulating the electronic structure of the Ir active center are effective strategies to improve catalytic reaction kinetics. For example, Wei et al. [111] reported a high loading of Ir active atoms (17.2% by weight) confined in an amino-functionalized carbon matrix (h-HL-Ir SACs) (Fig. 7c). Ir L_3_-edge X-ray absorption fine structure spectroscopy (XAFS) and N K-edge X-ray absorption near-edge spectroscopy (XANES) revealed Ir atomic dispersion, with an Ir–N coordination scattering peak observed at ~ 1.6 Å (Fig. 7d). The highly loaded Ir site significantly modulates the number of d-band holes and rapidly accumulates key oxygenated intermediates on the Ir sites, reducing *η*@10 mA cm^−2^ to 216 mV in acid. It is worth noting that single-atom Ir catalysts still require further optimization. The SACs are unable to contact intermediates at multiple sites simultaneously to release oxygen, which can lead to reductions in catalyst active sites or changes in the electronic structure, thus accelerating catalyst activity decay [154]. Furthermore, the dissolution or aggregation of limited catalyst active sites in SACs at high potentials remains a major challenge in PEMWEs.

Sub-nanocluster catalysts (SNCCs) (< 2 nm) have exhibited enhanced catalytic activity due to three distinct factors: unique geometrical and electronic structures, as well as synergistic interactions between atoms within the clusters [155, 156]. In comparison to nanoparticles and single-atom catalysts, loaded SNCCs exhibit higher atomic utilization efficiency, exposing a greater number of active atoms during catalytic reactions and providing numerous active sites for activating different intermediates [157]. The SNCCs with abundant interfacial metallic species are crucial for optimizing complex reaction steps through adsorption behavior. Modification is achievable through interactions of charge transfer and chemical bonding in interfacial areas, which can reduce the energy barriers associated with the reaction [158, 159]. However, synthesizing ultra-homogeneous and density-controlled nanoclusters remains challenging due to strong interatomic metallic bonds and high surface energy at high temperatures. Lee et al. [112] explored the relationship between particle size and atomic utilization, using size-limited COP as a carrier to flexibly modulate Ir loading and the surface density of Ir SNCCs (Fig. 7e–f). The prepared Ir SNCCs showed outstanding OER properties with *η*@10 mA cm^−2^ of 242 mV in acid (Fig. 7g). This is attributed to high surface atom utilization combined with modulation of surface Ir electronic states. Nevertheless, further research is needed to better understand the morphology-activity relationship of Ir-based SNCCs for regulating properties (such as the coordination number, bond length, electronic properties), cluster local environmental effects, and larger-scale SNCCs synthesis.

### Optimization of Structures

#### Surface Structures

During catalytic reactions, the surface structure of catalysts at gas/solid or liquid/solid interfaces has been extensively studied to enhance catalytic activity [160, 161]. The surface structure consists of several atomic layers where geometric configurations and elemental compositions vary significantly, influenced by reaction conditions and coordination environments [162]. Thus, catalytic activity can be enhanced through targeted modifications to the surface structure, exposing more active sites. More importantly, the complexity of the catalyst surface structure involves a wide range of possibilities for alloy or nonmetallic element composition and crystal states [163, 164]. Therefore, investigating the adsorption behavior within the local micro-environment at the atomic scale is critical for enhancing OER activity and developing more effective catalytic systems.

**Fig. 8:**
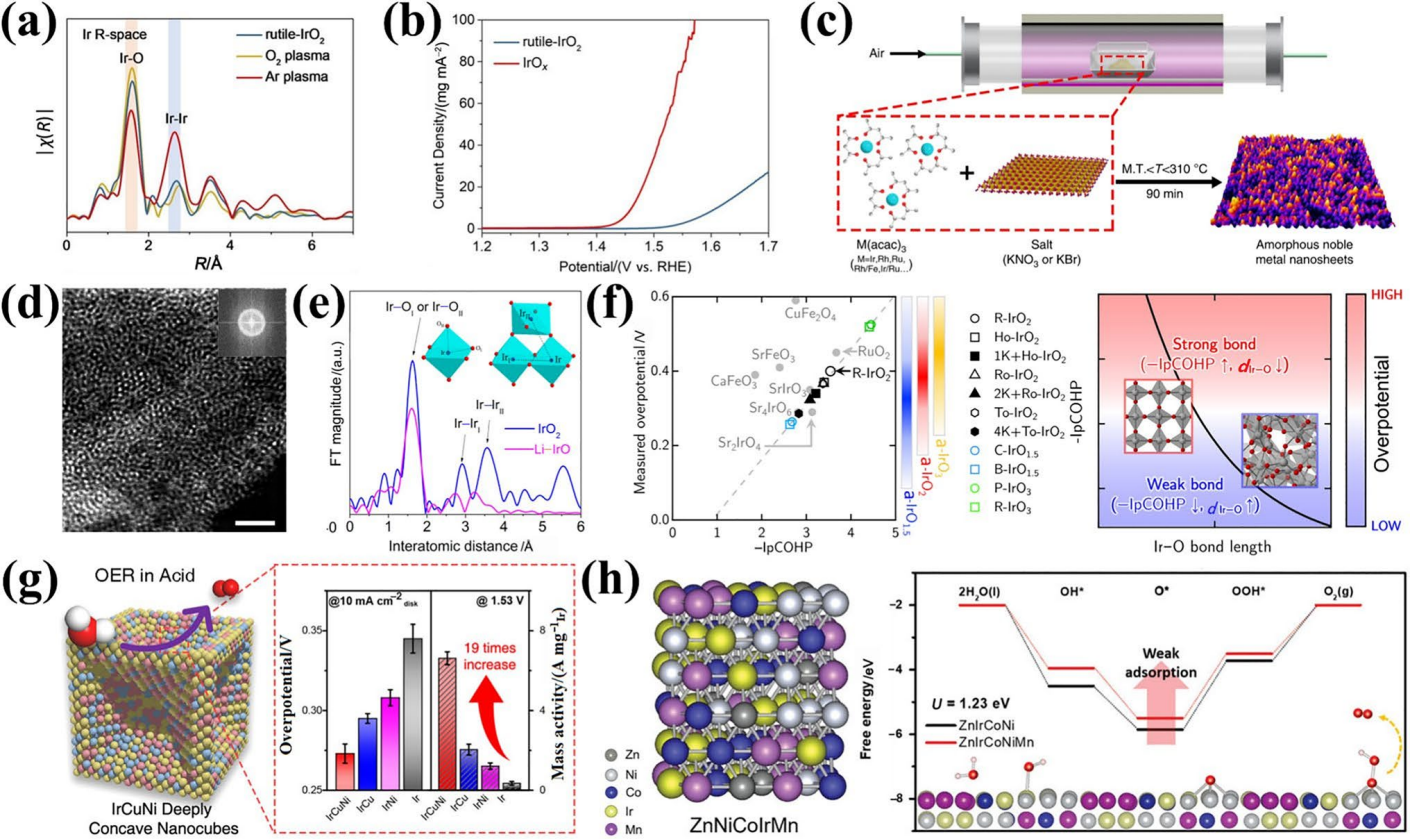
**a** FT-EXAFS spectra of Ir R-space for rutile-IrO_2_ and ones treated by O_2_ and Ar plasma. **b** LSV of targeted IrO_*x*_ and rutile-IrO_2_ samples. Reprinted with permission from Ref. [113]. Copyright © 2023, Wiley-VCH. **c** Schematic illustration of the general synthetic process of amorphous Ir NSs. Reprinted with permission from Ref. [114]. Copyright © 2019, Springer Nature. **d** HAADF-STEM images of Li-IrO_*x*_. **e** Connection modes of IrO_2_ and Li-IrO_*x*_ from EXAFS spectra. Reprinted with permission from Ref. [115]. Copyright © 2019, American Chemical Society. **f** Empirical regression model for structure-property relations in various OER oxide catalysts and schematic view of the empirical relationship. Reprinted with permission from Ref. [173]. Copyright © 2022, Springer Nature. **g** OER scheme of the IrCuNi DCNCs in acid. Reprinted with permission from Ref. [116]. Copyright © 2021, American Chemical Society. **h** Computational modeling of ZnNiCoIrMn and corresponding free energy of OER intermediates. Reprinted with permission from Ref. [117]. Copyright © 2023, Wiley-VCH

The crystal structures, chemical composition, and nanostructures of Ir oxide exhibit remarkable flexibility and complexity [115, 165, 166]. Rutile IrO_2_ exhibits poor intrinsic activity due to its high crystallinity and strong Ir–O bonds [102, 167]. The electronic structure of the Ir active site can be adjusted by altering the Ir–O coordination environment on the surface of rutile IrO_2_, thus optimizing the OER activity of IBEs. Wang et al. [113] combined plasma defect engineering techniques to systematically study the dependence between coordination numbers and activity of rutile IrO_2_ in acidic OER, then synthesized low-coordinated IrO_*x*_ nanoparticles (Fig. 8a). The low Ir–O coordination number IrO_*x*_ significantly alters the adsorption energy of oxygen intermediates, reducing the energy barrier of the rate-determining step. This results in enhanced activity, as evidenced by an *η*@10 mA cm^−2^ of 231 mV (Fig. 8b). Compared with high crystallinity IrO_2_, the amorphous IrO_2_ surface structure contains more active sites, providing numerous irregularly oriented bonds and defects due to disordered atomic arrangement in the amorphous structure [168–171]. In general, Ir-based nanomaterials are synthesized as crystalline structures, whereas preparing Ir in amorphous states requires simpler and more effective strategies [172]. High-yielding amorphous Ir nanosheets (Ir-NSs) with abundant active sites and unique atomic structures can be prepared via direct annealing of metal salts with alkali salts (Fig. 8c), exhibiting excellent properties [114]. To better design IBE surface structures in acidic media, further systematic studies are needed to clarify the potential relationship between amorphous iridium oxide activity and surface structure. Liu et al. [115] efficiently prepared amorphous Li-IrO_*x*_ via Li doping to explore its active structure (Fig. 8d). Amorphous Li-IrO_*x*_, composed of randomly connected [IrO_6_] octahedra, and rutile-IrO_2_, with ordered [IrO_6_] octahedra, share similar units but differ in arrangement (Fig. 8e). The disordered [IrO_6_] octahedra in Li-IrO_*x*_ exhibit greater flexibility, acting as more electrophilic centers. This results in a greater propensity for hydroxyl oxidation kinetics and promotes rapid water oxidation conversion. First-principles electronic structure calculations further reveal that non-equivalent connectivity in amorphous IrO_2_ enhances Ir charge state flexibility, enabling the formation of more electrophilic oxygen species critical for OER [173]. Combined with empirical regression models (Fig. 8f), these insights provide atomic-level explanations for the exceptional OER activity of amorphous Ir oxides.

Ir-based binary alloys have been extensively studied due to Ir alloying not only reducing the amount of precious metal Ir, but also adjusting the surface electronic structure of the catalyst and improving its intrinsic catalytic activity [147, 174–176]. More importantly, the atomic arrangement and composition of alloy surfaces dynamically evolve during electrocatalysis [177, 178]. Consequently, designing alloy surface structures is critical for enhancing catalytic performance, especially for complex multi-component alloy systems. Zhuang et al. [116] reported IrCuNi deep concave nanocubes (IrCuNi DCNCs) with an Ir-rich surface structure (Fig. 8g). Thanks to their open stepped surfaces with high surface area, low-coordinated atoms, and the alloying effect, these nanocubes exhibited excellent catalytic activity, achieving *η*@10 mA cm^−2^ of 273 mV at low Ir loading. High entropy alloy (HEA), defined as complex solid solutions comprising at least five principal elements, represents a unique class of materials with high chemical complexity and configurational entropy [179]. The interaction of multiple components on the surface of HEA can provide a synergistic effect, which can effectively improve the intrinsic performance of the electrocatalyst and reduce the amount of Ir [180, 181]. For example, Song et al. [117] prepared an Ir-based high entropy alloy (ZnNiCoIrMn) that showed an excellent catalytic performance with *η*@10 mA cm^−2^ of 237 mV. Incorporating Zn into the nanoporous structure provided structural benefits, while Mn addition induced a tuning effect on the electronic state of Ir sites, weakening the binding energy of *OH and *O intermediates (Fig. 8h). These findings highlight surface structure design as a viable strategy for developing cost-effective and efficient electrocatalysts for PEMWEs.

#### Compositional Structures

**Fig. 9:**
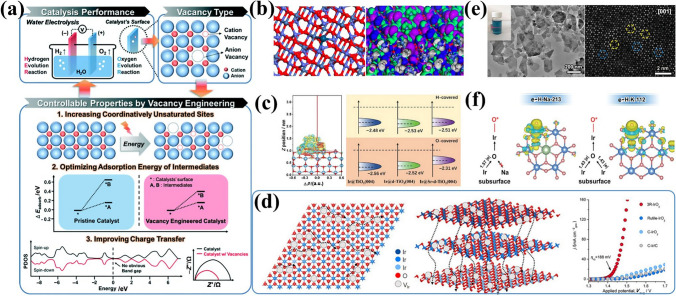
**a** Schematic illustrating the changes in the 9R-BaIrO_3_ crystal structure after HCl treatment, and the evolution of the surface structure during acidic OER. Reprinted with permission from Ref. [118]. Copyright © 2021, American Chemical Society. **b** Evolution of Sr_2_CaIrO_6_ in the electrolyte and during the OER. **c** LSV and Ir mass-specific OER activity for Sr_2_MIrO_6_ (M = Ca, Mg and Zn). **d** Evolution of the oxidation state (black) and the intensity (red) of the XANES signal during cycling. Reprinted with permission from Ref. [119]. Copyright © 2022, Springer Nature. **e** Electronic phase diagram and the corresponding band structures of Ir 5d orbitals. Reprinted with permission from Ref. [120]. Copyright © 2018, Wiley-VCH. **f** Crystalline structures and WT for Ir L-edge EXAFS for Y_2_Ru_1.2_Ir_0.8_O_7_. Reprinted with permission from Ref. [121]. Copyright © 2022, Wiley-VCH

Composition is also a key factor for catalytic activity, especially as some novel materials exhibit unique electronic structures and highly flexible structures, such as perovskites and pyrochlore IBEs [182]. Therefore, optimizing the composition to further improve activities has great potential in acidic OER. Recent studies have demonstrated that Ir-based perovskite oxides represent an emerging class of promising new electrocatalysts for OER in acid. These materials possess unique features that enable the reduction of the use of noble metals, resulting in enhanced mass activity [60, 183, 184]. The perovskite structure is a 3D framework of corner-sharing octahedra, classified into three main categories based on oxygen atoms per unit cell: single perovskite (ABO_3_), double perovskite (A_2_BB′O_6_), and layered perovskite (A_*n*+1_B_*n*_X_3*n*+1_) [185]. The Ir cation at the B-site usually determines the physical and chemical properties of Ir-based perovskite oxides, while the A-site cations influence valence states and oxygen vacancies at the B-site to enhance catalytic activity [186]. Therefore, more studies have been carried out to regulate the electronic structure and activity by controlling the A, B, B'-site cation composition [187, 188]. Simultaneously, understanding structural/compositional changes resulting from cation leaching in acidic solutions is important for the rational design of Ir-based perovskite oxides to enhance activities. Thomas F. Jaramillo et al. [165] first reported SrIrO_3_ perovskite oxides and found that the Sr leaching from the SrIrO_3_ surface leads to significant OER activity enhancement at Ir sites. Although the precise surface structure formed during the OER remains unclear, DFT calculations have revealed potential structures with high stability and activity. Yan et al. [118] employed acid treatment to leach Ba atoms and subsequently loaded highly active 1nm IrO_*x*_ particles onto the 9R-BaIrO_3_ surface (IrO_*x*_/9R-BaIrO_3_) (Fig. 9a). 9R-BaIrO_3_ has a high Ir mass activity (168 A g^−1^ (Ir)) and a low *η*@10 mA cm^−2^ of 230 mV, and its excellent activity arises from surface reconstruction into an amorphous Ir^5+^O_*x*_ active layer and enhanced conductivity during OER cycles. This deepens understanding of active layer structural evolution on the Ir-based perovskite surfaces. However, solubility differences of non-precious metal elements in Ir-based perovskite oxides lead to varied reconstructions. Elucidating the phase structural characteristics of reconstructed Ir-based perovskites is pivotal for optimizing their performance. Sergio Rojas et al. [119] synthesized the Ir-based double perovskites Sr_2_MIrO_6_ (M = Ca, Mg, Zn) and monitored surface reconstruction to ~ 2–3 nm using in situ/ex situ methods (Fig. 9b). Alkaline ion dissolution in Sr_2_MIrO_6_ allowed formation of a surface reconstructed with short-order of corner/edge-sharing IrO_6_ octahedra. The resulting amorphous Ir–O_*x*_-H_*y*_ surface layer is responsible for the observed high OER mass activity and low overpotential (Fig. 9c–d). Further optimizing Ir-based perovskite surface structures could unlock their full application potential [189].

In addition, pyrochlore oxides exhibit excellent activity potentials as OER catalysts in acidic solutions [190–192]. Pyrochlore oxides follow the formula A_2_B_2_O_7−*δ*_, where the A-site contains alkaline earth/rare earth metals, and B-sites contain Ir atoms as active sites [191, 193, 194]. A-site components modify B-site electronic structures, enabling OER activity regulation via cationic combinations at A/B sites [120, 195]. Notably, the interaction between electron correlation and spin-orbit coupling in the pyrochlore Ir oxide R_2_Ir_2_O_7_ (R = rare earth ion) can be tuned by changing R ions [196, 197]. Zeng et al. [120] prepared a series of R_2_Ir_2_O_7_ (R = Ho, Tb, Gd, Nd, or Pr) pyrochlore oxides to investigate activity-electronic correlation relationships. Results showed that the R ion radius significantly influences pyrochlore electronic properties and catalytic activity. Transitions from insulating to metallic states, coupled with enhanced hybridization between Ir 5d and O 2p orbitals (Fig. 9e), correlate with improved conductivity and Ir–O bond covalency, boosting catalytic activity. Moreover, developing highly active Ir pyrochlore complexes requires revealing crystal structure-OER activity relationships from a crystal chemistry perspective [198]. Optimizing structural design and bridge bonds between [IrO_6_] frameworks and A-site cations can effectively enhance potential activity. For example, Ir integration into A_2_Ru_2_O_7_ further enhanced the interaction between RuO_6_ and IrO_6_ by sharing oxygen and O–A–O bonds; the elongated Ir–O keeps Ir in a low oxidation state, facilitating *OOH intermediate formation (Fig. 9f) [121]. To date, Ir-based perovskite and pyrochlore oxides have activity capability as acidic OER electrocatalysts. However, their structural/compositional instability under acidic conditions leads to ambiguous activity metrics. Compared to IrO_*x*_ active layer formation, the contribution of intrinsic perovskite/pyrochlore structures to activity remains poorly understood.

### Modulation of Electronic Configurations

#### Defect Engineering

**Fig. 10:**
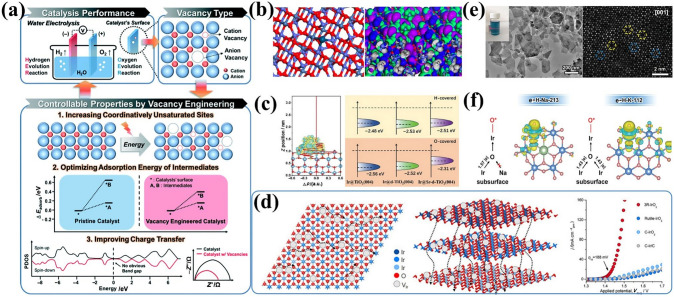
**a** Strategies of vacancy engineering for improving OER performance. Reprinted with permission from Ref. [208]. Copyright © 2020, The Royal Society of Chemistry. **b** Atomic Ir anchored near the O_V_ site on the NiCo_2_O_4_ surface and the modified orbital contour plot of the Ir-NiCo_2_O_4_-O_V_. Reprinted with permission from Ref. [122]. Copyright © 2022, American Chemical Society. **c** Charge distribution and *ε*_d_ of the O-covered Ir clusters in Ir@Sr-d-TiO_2_. Reprinted with permission from Ref. [123]. Copyright © 2024, Wiley-VCH. **d** Interlayers/intralayers proton transport pathway in 3R-IrO_2_ and OER performances. Reprinted with permission from Ref. [124]. Copyright © 2021, Elsevier. **e** TEM image and the HAADF-STEM image of e–H-Na-213 with intrinsic Ir vacancies. **f** Comparison of the charge density difference between e–H-Na-213 and e–H-K-112 in the oxygen adsorption configurations. Reprinted with permission from Ref. [125]. Copyright © 2024, Wiley-VCH

The high activity of IBEs is limited by the strong binding to O-related species, so optimizing the adsorption energy by modulating the electronic structure plays an important role in achieving excellent OER activities [21]. Defect engineering (e.g., anion and cation vacancies) is recognized as an effective strategy for regulating local electronic configurations and optimizing surface energies (Fig. 10a) [199–201]. OVs as typical anionic vacancies have been extensively studied due to their low formation energy and ease of construction [202, 203]. Moreover, OVs have the ability of accepting and giving electrons to promote H_2_O adsorption on catalyst surfaces, and facilitating intermediate adsorption/conversion [204]. Thus, rationally engineering oxygen vacancies is a viable strategy to optimize OER performance. Yan et al. [122] developed Ir single-atom on NiCo_2_O_4_ porous ultrathin nanosheets decorated with OVs (Ir-NiCo_2_O_4_ NSs) to effectively enhance OER activity in acid. DFT calculations showed that the presence of OVs makes the Ir site alter electron exchange and transfer in the low-coordination Co site (Fig. 10b), which is conducive to facilitating the initial H_2_O adsorption and further splitting activation. Additionally, oxygen vacancies boost catalyst intrinsic activity by inducing strong interactions near vacancies and adjusting band structures to optimize reaction intermediate adsorption [205]. Jiang et al. [123] prepared plasma-generated OVs on Sr-TiO_2_ nanowires with Ir nanoparticles (Ir@Sr-p-TiO_2_ NWs). Electron paramagnetic resonance (EPR) confirmed the presence of oxygen vacancies, which promoted Ir NP nucleation and growth within Sr-TiO_2_ nanowires. Results showed that OV concentration could modulate interactions between Ir NPs and Sr-p-TiO_2_ NWs, achieving an excellent *η*@10 mA cm^−2^ of 250 mV. The DFT calculation indicated that the OVs upshift the *ε*_d_ of Ir clusters and increase the energy cost of Ir demetallization (*E*_Ir_) (Fig. 10c), facilitating *OH adsorption and enhancing Ir cluster resistance to chemical corrosion for sustained activity.

The role of introducing cationic vacancy strategies in modulating electronic structures has also been widely explored [206, 207]. Shao et al. [124] synthesized a metastable 3R-phase IrO_2_ (3R-IrO_2_) with abundant Ir vacancies. In contrast to rutile-phase IrO_2_, Ir vacancies in 3R-IrO_2_ enable fast proton transport along interlayers and vertical directions, exhibiting an ultra-low *η*@10 mA cm^−2^ of 188 mV in acid (Fig. 10d). More importantly, intrinsic Ir vacancies can act as active sites to directly participate in reactions and regulate intermediate adsorption energy to enhance catalysis. Zou et al. [125] reported layered iridate Na_2_IrO_3_ nanosheets consisting of the edge Ir site adjacent to one intrinsic Ir vacancy (Fig. 10e). The DFT further proved that the carefully constructed Ir vacancy creates a unique local environment where individual Ir atoms donate electrons to surface edge Ir sites, weakening oxygen adsorption and enhancing OER activity (Fig. 10f). Vacancies effectively modulate catalyst electronic structures by accelerating electron interactions/migration, providing abundant active centers, and optimizing adsorption/surface energies. However, precise atomic-level control of specific defect sites and concentrations requires advanced synthetic and in situ characterization techniques to clarify the relationship between catalytic activity and specific defects, thereby optimizing reaction mechanisms.

#### Heteroatom Doping

**Fig. 11:**
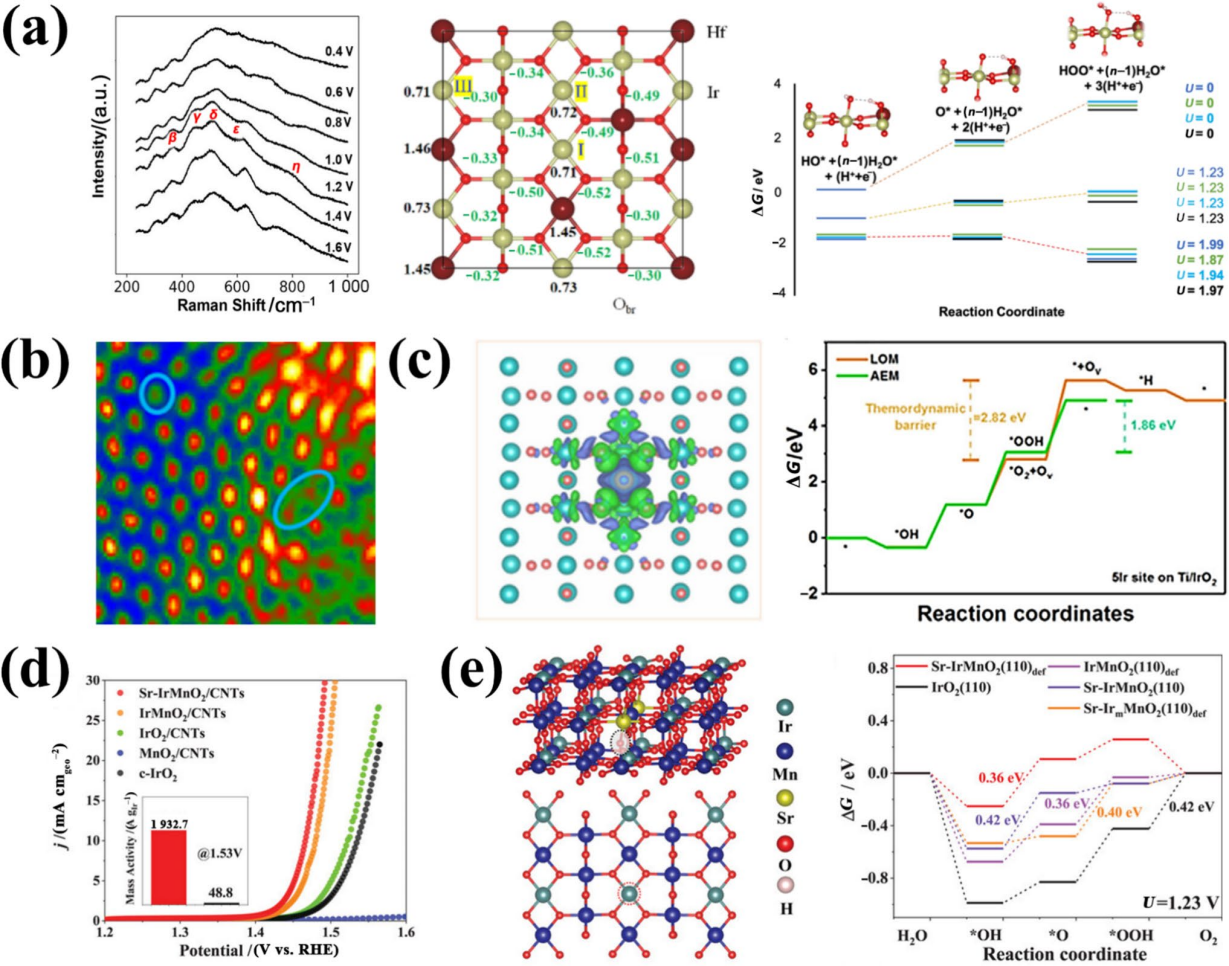
**a** Operando Raman, the model of Mulliken charges and free energy of Hf_0.25_Ir_0.75_O_2_. Reprinted with permission from Ref. [126]. Copyright © 2021, American Chemical Society. **b** Colored ATEM images with a temperature scale of Ti-IrO_*x*_/Ir. **c** Differential charge density distribution map and the PDOS of 5Ti-IrO_2_. Reprinted with permission from Ref. [127]. Copyright © 2023, Elsevier. **d** LSVs and mass activity of Sr-IrMnO_2_/CNTs. **e** Model and the free energy diagram of Sr-IrMnO_2_(110). Reprinted with permission from Ref. [128]. Copyright © 2023, Wiley-VCH

Doping heteroatoms into Ir or IrO_2_ frameworks represents a compelling approach to reduce Ir content and tailor electronic structures, particularly using cost-effective elements that exhibit robust activity and stability in strong acidic environments [26, 209–212]. Given that the number of d electrons in transition metal ions is closely related to the OER activity [213–215], electrocatalytic performance could be further optimized by introducing heteroatoms with specific valence electrons [216–218]. This strategic modification effectively improves adsorption characteristics of reaction intermediates, thereby optimizing the overall catalytic process [219–221]. Bruce E. Koel et al. [126] prepared early transition metal Hf-doped iridium oxide catalysts (IrHf_*x*_O_*y*_). The unique composite surface produced under acidic conditions exhibited an excellent mass activity of 6 950 A g^−1^ (IrO_*x*_) and *η*@10 mA cm^−2^ of 300 mV. Operando Raman measurements and DFT calculations indicate that Hf doping contributes to the formation of more negative charge states at adjacent O sites, shortening the Ir–O bond length and lowering the free energy of OER intermediates (Fig. 11a). This acceleration of the OER process is attributed to the altered electronic structure. While doping non-precious metals (e.g., Co, Fe, Ni, and Cu) has been shown to effectively increase OER activities by regulating Ir–O bond binding energy and the *ε*d location, doped metal leaching in acidic OER is inevitable [222–225]. Therefore, atomic-level catalyst understanding requires using corrosion-resistant metal doping techniques with fine-tuning. Xing et al. [127] doped Ti into the IrO_*x*_/Ir surface for creating rich Ir–O–Ti motifs (Fig. 11b), achieving an excellent OER activity with the *η*@10 mA cm^−2^ as low as 254 mV. The DFT reveals that OER promotion is a consequence of the electron-donating effect exerted by Ti upon neighboring Ir sites via the bridging oxygen in Ir–O–Ti. This phenomenon has the dual effect of weakening Ir-O interactions and increasing IrO_*x*_/Ir activities through AEM (Fig. 11c). Furthermore, Ti inhibits both over-oxidation of Ir and the occurrence of LOM, thereby reducing the dissolution of Ir. Dopant atoms must be well-dispersed in the matrix to optimize active sites. Due to differing ionic radii and electronic configurations, electron redistribution typically occurs locally, forming active sites with neighboring atoms [226]. Jiang et al. [128] constructed Sr-doped IrMnO_2_ solid solution fine nanoparticles on CNTs (Sr-IrMnO_2_/CNTs), where Ir atoms served as active sites with an Ir mass activity 39.6 times higher than IrO_2_ at 1.53 V (Fig. 11d). The solid solution structure homogeneously separates Mn and Ir in IrMnO_2_, thereby optimizing the electronic structure of each Ir atom through strong Ir–O–Mn bond coupling and lowering OER energy barriers (Fig. 11e). Sr addition enhances corrosion and demetallization resistance. In summary, dopant introduction enhances electrocatalytic performance through multiple mechanisms: adjusting electronic band structures, optimizing reactant/intermediate adsorption, increasing surface active sites, and minimizing catalytic site deactivation during reactions.

#### Strain Effect

**Fig. 12:**
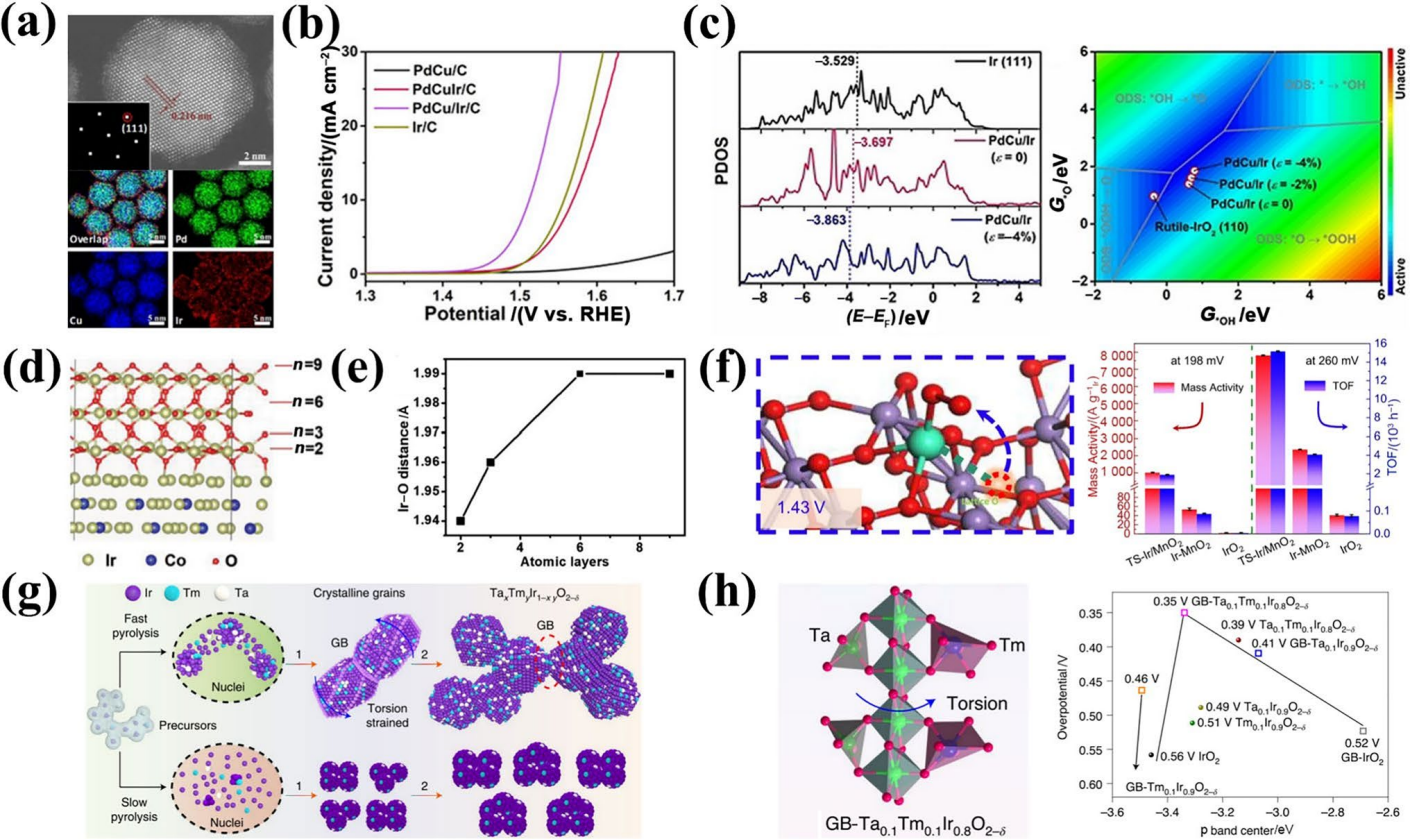
**a** Atomic-resolution HAADF-STEM and EDS mapping images of PdCu/Ir, and **b** OER performance. **c** Calculated d-band density of state (DOS) of PdCu/Ir and PdCu/Ir under 4% compression and the OER overpotential contour map in terms of the free energy of *OH and *O intermediates. Reprinted with permission from Ref. [129]. Copyright © 2021, Elsevier. **d** Schematic surface structure of IrCo@IrO_*x*_-nL NDs, and **e** band distance of Ir–O varying along the lattice compression. Reprinted with permission from Ref. [130]. Copyright © 2019, Wiley-VCH. **f** OER mechanism diagrams and mass activity of TS-Ir/MnO_2_. Reprinted with permission from Ref. [131]. Copyright © 2024, Springer Nature. **g** Schematic routes for synthesizing GB-Ta_0.1_Tm_0.1_Ir_0.8_O_2−*δ*_. **h** Illustration of strain effects and electron structure of GB-Ta_0.1_Tm_0.1_Ir_0.8_O_2−*δ*_. Reprinted with permission from Ref. [132]. Copyright © 2021, Springer Nature

Strain is typically attributed to lattice vacancies, lattice distortion, or lattice mismatch. The strategic exploitation of strain effects to alter the original electronic structure of materials to enhance catalytic activity has garnered considerable interest [227–229]. Lattice strain includes tensile and compressive strains, in which strain-induced changes in adjacent metal atom numbers cause shifts in the *ε*d. Tensile strain reduces coordination numbers, decreasing bandwidth while increasing the *ε*d value [227]. Conversely, compressive lattice strain enhances metal d-orbital overlap, strengthening adsorbent-metal interactions and reducing adsorbate binding energy [230]. Strain effects are particularly significant in core-shell nanocatalysts due to lattice parameter variations at core-shell interfaces [166, 231–233], including the core-shell lattice mismatch, shell thickness/growth mode, and core morphology [234]. Controlling shell epitaxial growth is key to regulating strain states. Guo et al. [129] employed precise modulation technique to create heterogenous epitaxial Ir shells on PdCu nanocrystals (Fig. 12a), yielding highly strained PdCu/Ir core/shell nanocrystals with significant research and technological potential. The 3.60% compressive strain induced by Ir shell lattice mismatch shows that PdCu/Ir core/shell nanocrystals exhibit an excellent *η*@10 mA cm^−2^ of 283 mV in acid (Fig. 12b). DFT indicates that Ir shell compressive strain reduces oxygen-containing intermediate adsorption strength, causing d-band center downshifting to promote oxygen molecule production (Fig. 12c). Adjusting shell layer numbers to control strain is an attractive strategy to enhance OER activity. For instance, Li et al. [130] demonstrated electrochemical control over IrO_*x*_ atomic layer growth on IrCo substrates (Fig. 12d), enabling IrCo gradient strain to optimize Ir–O bond lengths in IrO_*x*_. The ≈3-atom layer IrO_*x*_ on IrCo nanodendrites (IrCo@IrO_*x*_-3L NDs) achieved *η*@10 mA cm^−2^ of only 247 mV with long-term stability due to the optimal compressive strain and Ir–O bond length (Fig. 12e). The DFT shows compressive strain modulates *OOH binding strength on IrO_*x*_ surfaces, facilitating the *O to *OOH formation and enhancing OER activity. Optimizing Ir–O bonds under lattice strain is critical, as it disrupts linear relationships among multiple intermediates, while minimizing Ir active site dissolution is pivotal for maintaining high activity. Liu et al. [131] confined Ir atoms to tensile-strained manganese oxide (TS-Ir/MnO_2_), which enhanced the covalency of Ir–O_4_ and improved the rapid conversion of *OH at the surface OV. TS-Ir/MnO_2_ follows a continuous localized LOM mechanism to effectively stabilize surface Ir active sites, exhibiting excellent mass activity of 1 025 A g^−1^ (Ir) and *η*@10 mA cm^−2^ of 198 mV (Fig. 12f). Beyond traditional compressive/tensile strains, introducing flexible/controllable strains into crystals can optimize catalysis. Jin et al. [132] constructed torsion-strained GB-Ta_0.1_Tm_0.1_Ir_0.8_O_2−*δ*_ twinned crystal with abundant grain boundaries (GBs), stacking faults (SFs) and dislocations by incorporating Ta/Tm into IrO_2_ using a fast pyrolysis strategy (Fig. 12g). Torsional strain arises from neighboring crystal grain collisions/mergers on short timescales, modifying Ir–O bond lengths and electronic structures through principal GB-based torsional strain (Fig. 12h).

In summary, electronic structure modulation is pivotal for optimizing IBEs activity and stability. Defect engineering, heteroatom doping, and strain effects collectively fine-tune Ir electronic states to enhance performance. Introducing oxygen/Ir cationic vacancies improves conductivity and active site density via local charge modulation, though excessive defects risk structural degradation and Ir dissolution. Heteroatom doping with transition metals (e.g., Ru, Sn, Mo, and W) optimizes OER intermediate (*OH, *O, and *OOH) adsorption energetics by shifting the d-band center, tailoring Ir–O bond strength to balance kinetics and stability. Strain modulation via lattice parameter adjustments, oxide supports, or core-shell architectures influences orbital hybridization: compressive strain strengthens Ir–O bonds for durability, while tensile strain weakens them to boost activity. These synergistic approaches modulate electronic states, charge transfer, and reaction energetics to enhance OER kinetics with minimal degradation. Strategically integrating these strategies advances IBE deployment in PEMWEs by balancing high activity and long-term stability.

## Approaches to Enhance Stability of IBEs

While numerous studies have concentrated on strategies to improve the activity of IBEs, it is equally important to emphasize the improvement of catalyst stability under harsh acidic conditions. In some instances, a negative correlation between stability and activity has been observed, which can be attributed to the density of surface defects and atomic coordination [76, 235]. Nevertheless, a substantial body of research indicates that activity and stability can also be independently modulated [236–238]. With the development of various operando techniques and theoretical calculations [82, 239–242], the dynamic degradation behavior of IBEs in acid has been more comprehensively studied and theoretically supported, and new insights have been gained in terms of stability. Therefore, we first summarize the stability of current advanced IBEs in Table 4 and focus on the recent research advances regarding effective strategies to stimulate the OER stability capability. These include phase engineering, support effects and electronic interactions.

**Table 4:** Stability of current advanced IBEs for electrocatalysts in acidic media

Catalyst	Electrolyte	loading/ (mg cm^−2^)	Stability performance	Test conditions	References
6H-phase SrIrO_3_	0.5 mol L^−1^ H_2_SO_4_	0.9	degradation of ~ 20 mV after 30 h	CP@10 mA cm^−2^	[243]
1 T(trigonal)IrO_2_	0.1 mol L^−1^ HClO_4_	0.2	98% current retention after 45 h	CP@10 mA cm^−2^	[244]
3R (rhombohedral)-IrO_2_	0.1 mol L^−1^ HClO_4_	0.5	degradation of 30 mV after 511 h	CP@10 mA cm^−2^	[124]
IrO_2_ nanoribbons	0.5 mol L^−1^ H_2_SO_4_	0.2	98.4% current retention after 139 h	CP@10 mA cm^−2^	[245]
IrO_*x*_·*n*H_2_O	0.1 mol L^−1^ HClO_4_	1.5	no apparent degradation after 5 700 h; S-number 1.9 × 10^7^	CP@10 mA cm^−2^	[246]
W-Ir-B alloy	0.5 mol L^−1^ H_2_SO_4_	0.25	degradation of 3.2 mV after 800 h	CP@100 mA cm^−2^	[247]
IrIn_2_/C	0.5 mol L^−1^ H_2_SO_4_	~ 0.1	no apparent degradation after 55 h	CP@10 mA cm^−2^	[248]
2D fcc-Ru_3_Ir	0.5 mol L^−1^ H_2_SO_4_	0.2	degradation of 14 mV after 10 000 cycles and 400 h	10 000 cycles and CP@10 mA cm^−2^	[249]
IrO_2_@TaB_2_	0.1 mol L^−1^ HClO_4_	0.3	no apparent degradation after 120 h; S-number 5.2 × 10^4^	CP@10 mA cm^−2^	[250]
Ir/Nb_2_O_5−*x*_	0.5 mol L^−1^ H_2_SO_4_	0.45	87.5% current retention@1.7 V; S-number 1.6 × 10^5^	CP@10 mA cm^−2^ and CA@1.7 V for 5 h	[251]
Ir:WO_3_/Ir	0.5 mol L^−1^ H_2_SO_4_	0.000 72	99.9% current retention after 780 h	CP@10 mA cm^−2^	[252]
Ir-MoO_3_	0.5 mol L^−1^ H_2_SO_4_	~ 0.1	degradation of ~ 50 mV after 48 h	CP@10 mA cm^−2^	[253]
Ir-MnO_2_	0.5 mol L^−1^ H_2_SO_4_	1	degradation of 15 mV after 650 h	CP@10 mA cm^−2^	[254]
IrMnOF@Ir	0.1 mol L^−1^ HClO_4_	~ 1	degradation of ~ 50 mV after 200 h	CP@10 mA cm^−2^	[255]
Gd-IrO_2–*δ*_	0.5 mol L^−1^ H_2_SO_4_	1	degradation of 56 mV after 200 h	CP@10 mA cm^−2^	[256]
sl-Mn_0.98_Ir_0.02_O_2_	0.5 mol L^−1^ H_2_SO_4_	0.5	86% current retention after 168 h	CP@10 mA cm^−2^	[257]

Data are extracted from different resources, thus with different significant digits

### Phase Engineering

#### New Phases

**Fig. 13:**
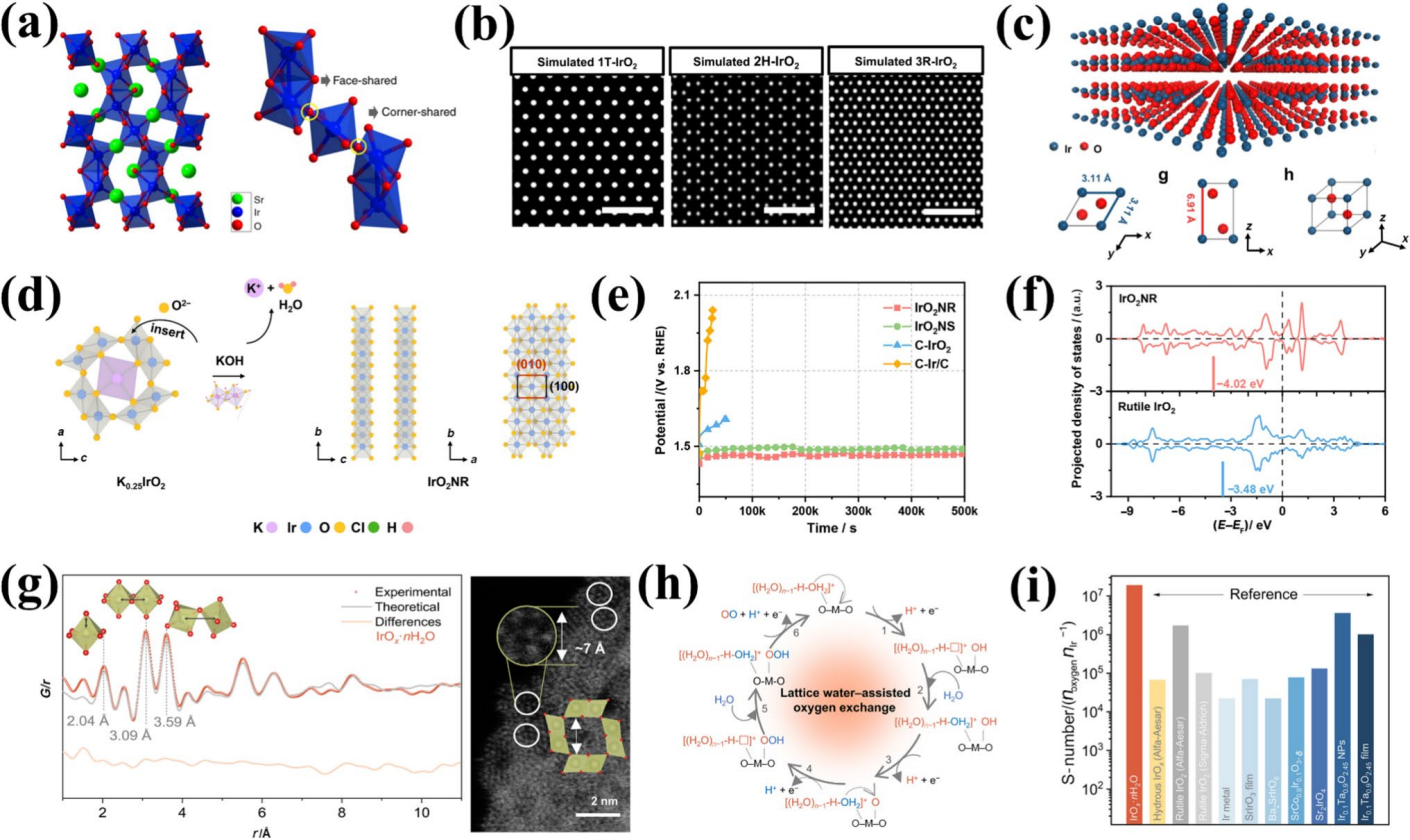
**a** Crystal structure and IrO_6_ octahedral local connection pattern of 6H-SrIrO_3_. Reprinted with permission from Ref. [243]. Copyright © 2018, Springer Nature. **b** Simulated TEM images of IrO_2_ with 1T, 2H and 3R phases. **c** Atomic structure of layered 1T-IrO_2_. Reprinted with permission from Ref. [244]. Copyright © 2021, Springer Nature. **d** Illustration of the crystal structure for the IrO_2_NR. **e** CP curves of the IrO_2_NR at a constant current density of 10 mA cm^–2^. **f** Comparison of the d-orbital distribution of the Ir atoms in the rutile IrO_2_ and IrO_2_NRs. Reprinted with permission from Ref. [245]. Copyright © 2023, Springer Nature. **g** Crystal structure schematic and HAADF-STEM images of IrO_*x*_·*n*H_2_O. **h** Schematic for lattice water-assisted oxygen evolution. **i** S-number of IrO_*x*_·*n*H_2_O and it is compared with commercial IrO_*x*_. Reprinted with permission from Ref. [246]. Copyright © 2023, the American Association for the Advancement of Science

Crystal phase engineering has emerged as a pivotal strategy for enhancing the stability of electrocatalysts, as it enables the manipulation of coordination numbers, morphology, and the distribution of surface atoms through electronic modifications and geometric configuration of catalysts [258]. Rutile-phase IrO_2_, characterized by its natural thermodynamic stability because its primitive regularly arranged [IrO_6_] units connected by shared edge and corner are inherently stable [198, 243]. However, its practical applicability is limited by slow kinetic rates [259, 260]. Notably, the stability of certain phases has been found to increase as the thickness of the crystalline layers decreases. This observation suggests that engineering novel, thin-layered phases of IrO_2_ constitutes an effective strategy for achieving enhanced phase stability. For instance, Zou et al. [243] identified a 6H-phase SrIrO_3_ perovskite (6H-SrIrO_3_) with excellent catalytic stability in acid (Fig. 13a). Later, Shao et al. [124, 244] synthesized a series of novel IrO_2_ phases with 2D nanosheet morphologies, namely 1T, 2H, and 3R (Fig. 13b). Different from rutile-phase IrO_2_, layered 1T-IrO_2_ with AA stacked phase structure and 3R-IrO_2_ with the trigonal ABC ordering have excellent OER stability derived from the novel Ir site of the edge-shared [IrO_6_] octahedra (Fig. 13c). In addition, metastable nanostructures have the potential for superior catalytic performances because they have different unit linkages that can provide completely distinct stability for electrocatalysis [261–263]. Recently, a metastable monoclinic *C*2/*m* (12) phase layered IrO_2_ nanoribbon (IrO_2_NR) has been prepared by the O^2−^ from KOH attacking the corner-connected octahedron of K_0.25_IrO_2_ at high temperature (Fig. 13d) [245]. Due to the specific phase structure and nanoribbon morphology, IrO_2_NRs exhibit excellent OER stability in 0.5 mol L^−1^ H_2_SO_4_ and the overpotential increases only ~ 1.6% after 138 h (Fig. 13e). In addition, the higher number of edge-exposed Ir active sites in monoclinic IrO_2_NRs has lower d-band energy levels than rutile-phase IrO_2_ (Fig. 13f), resulting in weaker adsorption of OER intermediates (e.g., *O) and a more favorable free energy distribution, enabling low-overpotential catalysis. Based on the stability advantages demonstrated by edge-shared [IrO_6_] octahedra, it is of paramount importance to further optimize the oxygen exchange behavior to facilitate the participation of a greater number of active O_L_ in the reaction, while maintaining the crystal structure stable. Qiao et al. [246] constructed lattice water-assisted Ir oxides (IrO_*x*_·*n*H_2_O) by placing lattice water into the crystalline phase with an edge-shared [IrO_6_] octahedron frame (Fig. 13g). This structural design effectively stabilizes the charge distribution and enhances durability (Fig. 13h), achieving an S-number two orders of magnitude higher than commercial IrO_*x*_ and demonstrating ultra-long-term operational stability exceeding 5 700 h (~ 8 months) (Fig. 13i), and providing a cell voltage of 1.77 V at 1 A cm^−2^ for 600 h (60 °C) without significant structural degradation. Therefore, the study of IrO_2_ new phase materials is significant in the enhancement of IBEs stability in acid.

#### Alloy Phase

**Fig. 14:**
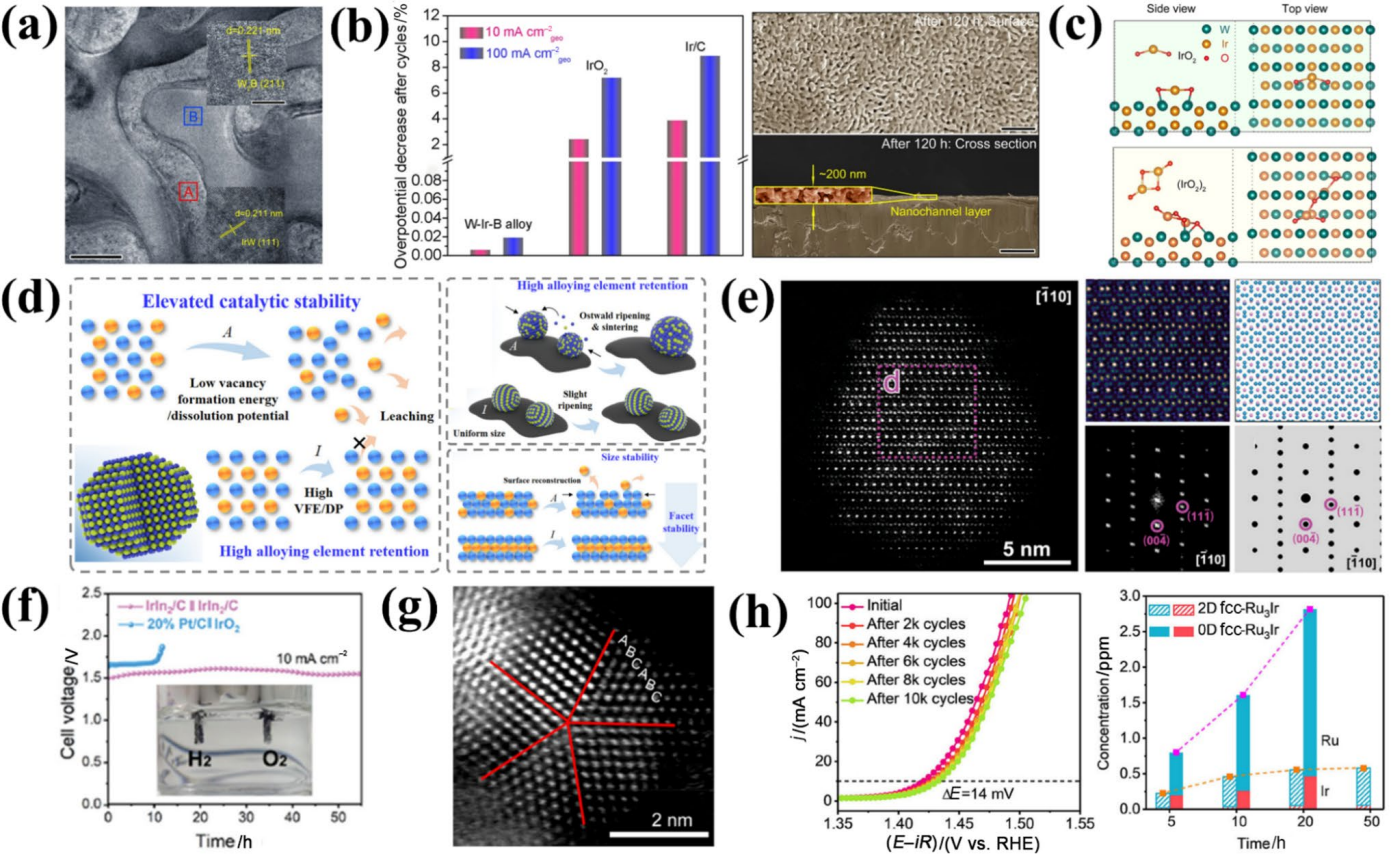
**a** TEM image and the HRTEM image of the phase-separated W-Ir-B alloy. **b** Activity degradations and cross-sectional SEM images of W-Ir-B alloy after the long time OER test. **c** Optimized structures of the (IrO_2_)_*n*_ clusters adsorbed to the IrW (002) surface. Reprinted with permission from Ref. [247]. Copyright © 2021, Springer Nature. **d** Strategies for improving catalytic stability derived from intermetallic compounds. Reprinted with permission from Ref. [271]. Copyright © 2023, American Chemical Society. **e** AC-HAADF-STEM image and the corresponding simulation structure of IrIn_2_ nanoparticle. **f** CP tests of IrIn_2_/C during water electrolysis. Reprinted with permission from Ref. [248]. Copyright © 2023, Wiley-VCH. **g** Structure diagram and AC-HAADF-STEM images of 2D fcc-Ru_3_Ir. **h** OER polarization curves and leached Ir ions from 2D fcc-Ru_3_Ir after long time cycles; 1 ppm = 1 × 10^−6^. Reprinted with permission from Ref. [249]. Copyright © 2023, American Chemical Society

The degradation behavior of Ir is intricately related to its coordination environment, which determines the interaction of Ir with water [92]. By engineering the phase structure of Ir alloys, it is possible to significantly alter the number of exposed active sites and modify the surface conformation of Ir, thereby affecting both the structural integrity and long-term durability of the catalyst [117, 237, 264, 265]. Xiong et al. [247] developed a biphasic IrW-W_2_B alloy (W-Ir-B) by continuous selective etching of W_2_B, where an electrocatalytically active IrW nanochannel layer with a depth of ~ 200 nm was formed as an efficient and ultra-stable IBE in acid (Fig. 14a). Furthermore, W-Ir-B was demonstrated to be capable of maintaining a current density of 100 mA cm^−2^ for 800 h with an extremely low degradation rate (~ 4 μV h^−1^) (Fig. 14b). Based on surface active structure analysis and DFT calculations, the high stability originates from the IrW nanochannel changing the charge distribution of surface Ir and O atoms and limiting the size of IrO_2_ clusters (Fig. 14c). Thus, it can be observed that the Ir atoms are unable to form soluble high-valence counterparts when they accumulate more O atoms, and stable IrO_2_ clusters are retained on the substrate. In contrast to random alloys, intermetallic compounds (IMCs) have significant structural and property stability in harsh acidic electrochemical environments (Fig. 14d), which is attributed to the heteroatom bonding in intermetallic compounds having a more negative enthalpy of formation and periodic arrangement of ordered atoms inhibiting atomic migration in the alloy phase [266, 267]. Lu et al. [248] prepared a low-iridium-content IrIn_2_ IMC anchored on carbon with a face-centered orthogonal (fco) structure (IrIn_2_/C) (Fig. 14e), which exhibited long-term stability for 55 h in 0.5 mol L^−1^ H_2_SO_4_ without significant performance or structural degradation (Fig. 14f). The highly ordered arrangement of the two atoms has a stronger Ir–In bond, which helps stabilize the local coordinating environment of the active site and the interface structure during catalysis. In general, bulk materials exhibit good stability due to their low surface energy, whereas conventional alloy nanomaterials tend to favor thermodynamically stable phases, such as face-centered cubic (fcc) structures [268]. Importantly, the surface energy begins to dominate the system energy at the nanometer scale, which allows the stability of alloy nanomaterials to be achieved by regulating the surface energy [269, 270]. Cui et al. [249] synthesized quasi-two-dimensional Ru_3_Ir alloys (2D fcc-Ru_3_Ir) with unconventional fcc by a phase-modulation strategy (Fig. 14g). The unconventional fcc structure of 2D fcc-Ru_3_Ir with low surface energy suppresses the over-solution during OER, thereby ensuring excellent stability with negligible degradation of the catalyst's activity over 400 h or 10 000 potential cycles (Fig. 14h).

### Supports Effect

#### Interface Connection

**Fig. 15:**
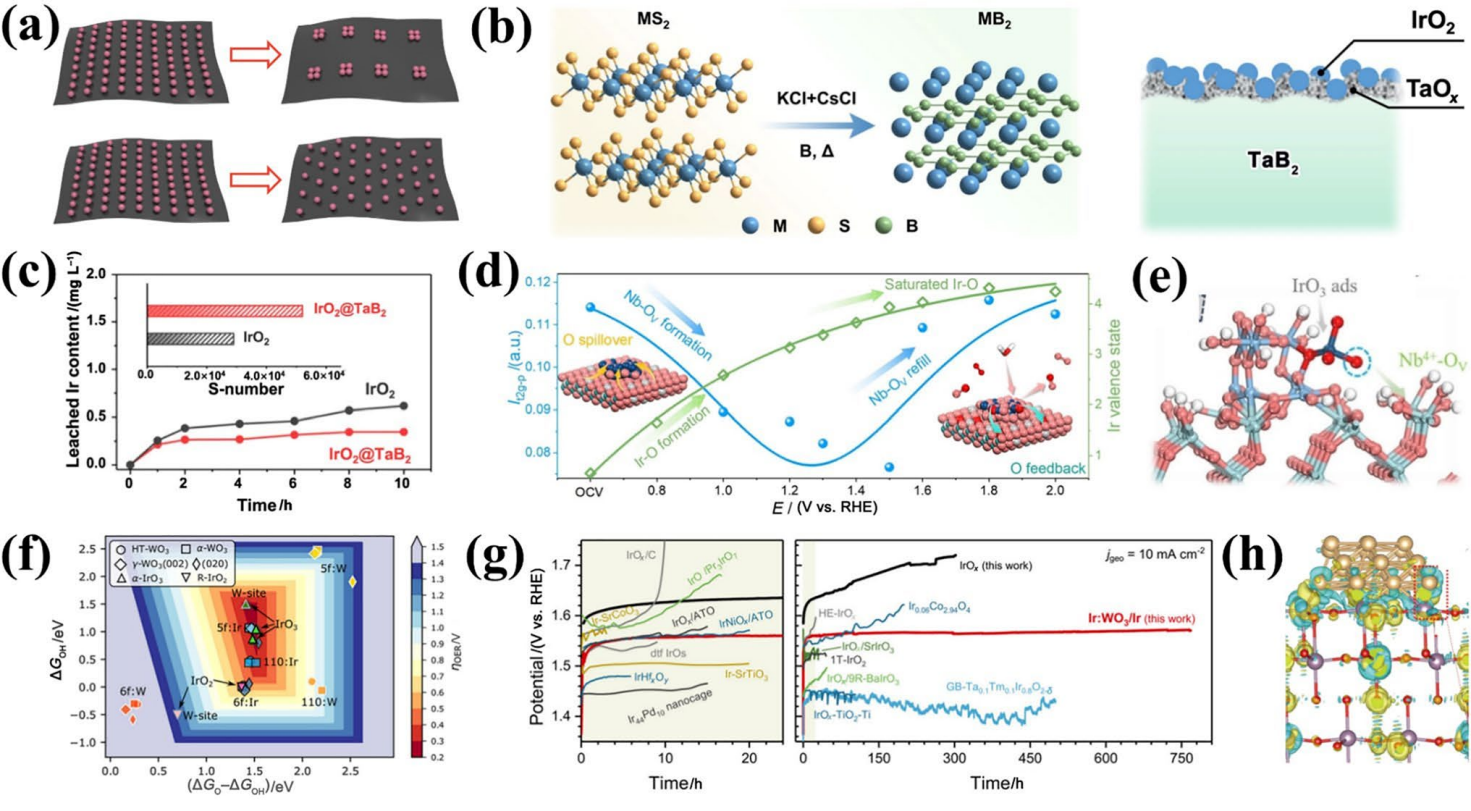
**a** Illustration of the aggregation and dissolution of nanostructured active species in supports. **b** Schematic illustration of the synthesis and crystal structures of IrO_2_@TAB_2_. **c** Contents of leached iridium in the electrolyte of IrO_2_@ TAB_2_ during long time tests. Reprinted with permission from Ref. [250]. Copyright © 2023, Springer Nature. **d** Illustration of the overall dynamic interface effect. **e** Ir on Ir/Nb_2_O_5−*x*_ considering the dynamic migration of oxygen species. Reprinted with permission from Ref. [251]. Copyright © 2022, Wiley-VCH. **f** Summary of the theoretical OER activity of various doping models with IrO_3_. **g** Durability test for Ir:WO_3_/Ir in acid OER. Reprinted with permission from Ref. [252]. Copyright © 2022, American Chemical Society. **h** Charge density difference of IMO with an electron-deficient surface. Reprinted with permission from Ref. [253]. Copyright © 2021, Springer Nature

The incorporation of support materials represents an effective strategy for enhancing both the utilization and stability of precious metal catalysts under harsh acidic conditions. The performance of catalyst/support systems is highly dependent on the intrinsic properties of the support material, including chemical stability, electrical conductivity, and the nature of catalyst-support interfacial interactions [272–274]. In general, the reduced stability of nanostructured active materials during the OER is often attributed to the aggregation and dissolution of active sites (Fig. 15a) [92]. Moreover, anchoring nanostructured active sites on the support surface to achieve homogeneous dispersion can effectively enhance the stability [275, 276]. The use of high electrical conductivity, large specific surface area, and excellent thermal stability of support materials has become the key to the preparation of ultra-uniform and density-controlled IBEs, in which carbon as the most typical electrocatalyst carrier is widely used in various reactions [277–279]. However, carbon support is prone to low stability problems at high operating potentials of OER and in strongly acidic media [280, 281]. Consequently, the development of alternative non-carbon support materials is a prerequisite for achieving stable catalyst-support interfaces and enhancing long-term performance in PEMWEs. For example, Zou et al. [250] prepared a high-surface-area and high-conductivity nano-metal diborides (TaB_2_) as the carrier of IrO_2_ nanocatalysts to achieve enhanced corrosion resistance of IrO_2_ (Fig. 15b). The interfacial electron interaction between IrO_2_ and TaB_2_ leads to charge redistribution of IrO_2_ and the reduction of Ir valence states. Therefore, the TaB_2_ carrier greatly improves the catalytic stability of IrO_2_ in the acidic OER process, where the S-number is 5.2 × 10^4^ higher than that of IrO_2_ (2.9 × 10^4^) (Fig. 15c). In addition, metal oxide supports with stronger corrosion resistance have been widely investigated, such as SiO_2_ [282], TiO_2_ [283], antimony or fluorine-doped tin oxide (ATO [284] or FTO [273]). These metal oxides can also modulate the electronic structure of the active sites through interfacial electronic interactions, thereby promoting electrochemical stability and accelerating charge transfer kinetics [274]. Generally, the electrochemically generated Ir^*n+*^O_*x*_ in Ir-based catalysts/supports is used as the actual active site [284, 285]. Establishing both physical and electronic coupling between IrO_*x*_ and support materials is a promising approach for enhancing catalyst stability, particularly through supports capable of self-redox or surface redox cycling (e.g., Ti^3+^/Ti^4+^, Nb^4+^/Nb^5+^, Mo^5+^/Mo^6+^) [286–289]. Xing et al. [251] used Ir-loaded Nb_2_O_5−*x*_ as a model catalyst to construct a catalyst/support interface connection, i.e., a dynamic interfacial oxygen migration mechanism. Oxygen can migrate from Nb_2_O_5−*x*_ nanoparticles to Ir for forming Ir–O coordination structures, and the excess O on the Ir surface can feed back to Nb^4+^ (Fig. 15d). Theoretical simulations further demonstrate that the dynamic migration of interfacial oxygen is energetically favorable under working conditions. Moreover, it is effective in keeping the Ir sites in a low redox state at challenging high potentials through the feedback mechanism of oxygen species (Fig. 15e), thus ensuring the stability of IrO_*x*_. The dynamic interactions at the catalyst/support interface could provide valuable insights for the design and enhancement of catalytic performance of OER.

#### Modified Valence States

In general, changes in the valence state of the active site influence the electronic configuration, thereby impacting the structural stability of the active site [290]. The judicious selection of support materials can effectively modulate the oxidation state of iridium through electronic interactions at the metal-support interface, enhancing its resistance to acidic environments [284]. Numerous experimental studies have shown that highly oxidized Ir sites exhibit superior OER properties. Additionally, some polycrystalline states of IrO_3_ have demonstrated acid stability at the required oxidation potentials for OER [86, 291, 292]. Michal Bajdich et al. [252] created and stabilized high-valence Ir sites by tuning the interaction between Ir species and WO_3_ substrates (Ir:WO_3_/Ir), resulting in an efficient and stable acidic OER. Among others, DFT calculations have revealed that the high-valence Ir sites can optimize the adsorption energy of the key intermediates involved in OER and facilitate the formation of these sites on the WO_3_ surface (Fig. 15f). Ir:WO_3_/Ir has very high mass activity (13.8 A mg^−1^(Ir)) and stability for 32 days in 0.5 mol L^−1^ H_2_SO_4_ (Fig. 15g). Furthermore, in-depth studies also revealed that the high oxidation state of Ir (> 4) is accompanied by a greater number of electron holes in the Ir 5d orbital than Ir ($\leqslant$ 3) [293]. As such, the formation of an electron-deficient Ir surface via support-induced electronic modulation represents a viable strategy for improving the oxidative durability of IBEs. For instance, Lee et al. [253] reported catalysts composed of highly electron-deficient metal Ir on the electron-withdrawing material of MoO_3_ (IMO). The IMO with an electron-deficient Ir^>4+^ surface was formed due to the oxygen groups on the IMO surface inducing electron transfer from Ir to O and the electron withdrawal from Ir NPs by MoO_3_ (Fig. 15h). The IMO has the *η*@10 mA cm^−2^ of only ~ 156 mV and excellent stability in acid due to the synergistic effect of the high oxidation state of Ir and MoO_3_, which can tolerate the resistance of the oxidation state.

### Electronic Interactions

#### Metal–Oxygen Orbital Hybridization

**Fig. 16:**
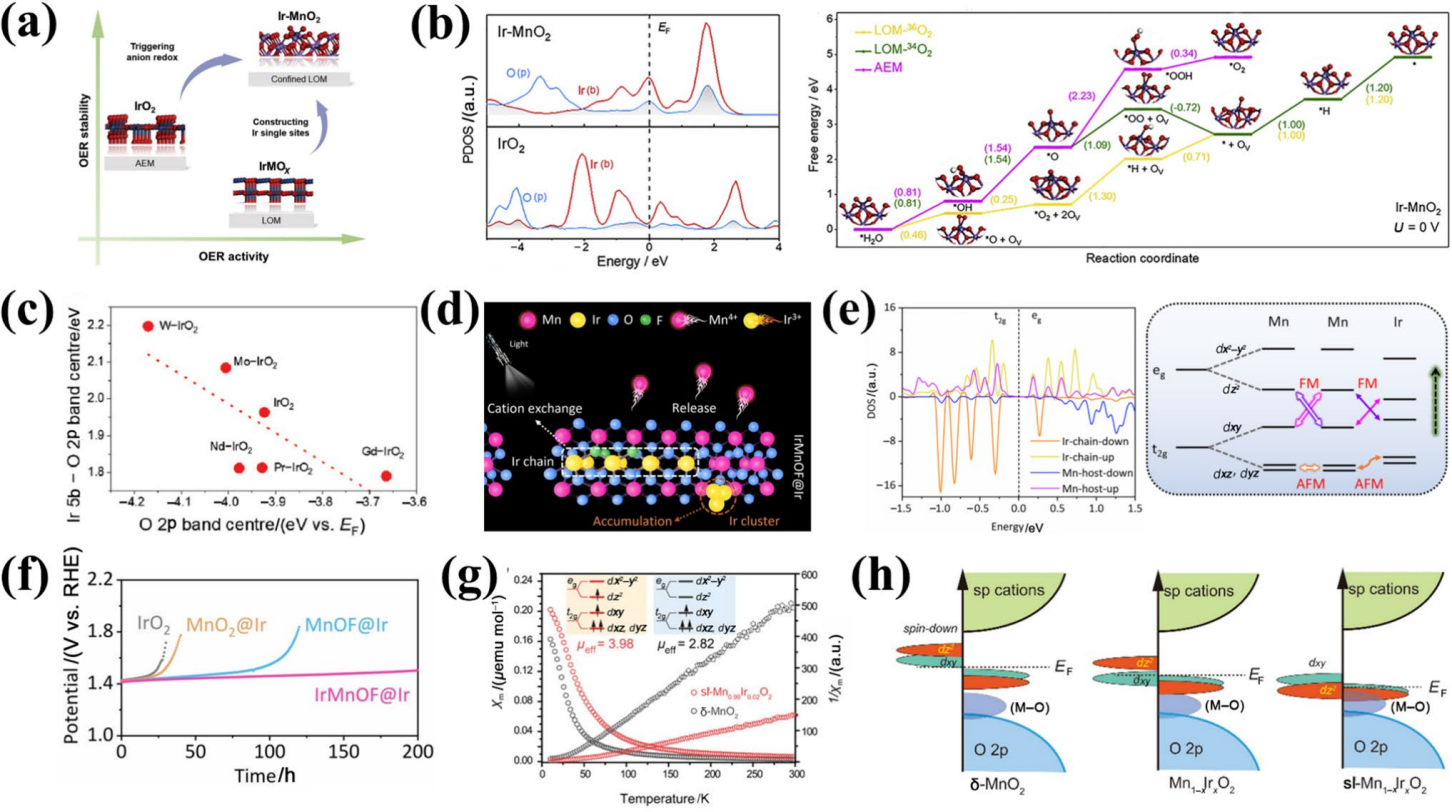
**a** Design schematic of Ir-MnO_2_. **b** Projected DOS plots and the Gibbs free energy diagram for OER on Ir-MnO_2_ based on AEM and LOM. Reprinted with permission from Ref. [254]. Copyright © 2021, Elsevier. **c** Summary of Ir–O covalency of difference metals doped IrO_2_. Reprinted with permission from Ref. [256]. Copyright © 2023, Wiley-VCH. **d** Schematic of light-driven IrMnOF@Ir synthesis. **e** Spin-resolved PDOS and the schematic diagram of d orbital splitting and the magnetic exchange sketch of IrMnOF@Ir. **f** Stability test of IrMnOF@Ir. Reprinted with permission from Ref. [255]. Copyright © 2023, Wiley-VCH. **g** Temperature dependence of molar magnetic susceptibility for sl-Mn_0.98_Ir_0.02_O_2_. **h** Electronic structure derived from PDOS of sl-Mn_0.98_Ir_0.02_O_2_. Reprinted with permission from Ref. [257]. Copyright © 2023, Wiley-VCH

Numerous research strategies have leveraged the LOM to enhance the OER kinetics of IBEs. One effective strategy involves strengthening the metal–oxygen (M–O) covalent bond, which significantly improves OER activity [294, 295]. This improvement arises from the upward shift of the O 2p band toward the Fermi level (FL), which enhances the orbital overlap between the metal d-band and the oxygen 2p orbitals, thereby facilitating charge transfer during the reaction [296–298]. This enhancement in M–O bond covalency makes the lattice oxygen redox energetically more favorable [299–301]. However, the enhanced OER kinetics usually comes at the cost of structural instability [63]. Excessive covalency can lead to surface amorphization or adverse structural transformations. Furthermore, the dynamic formation of numerous O_V_, which stimulates the migration of bulk O_L_ to the surface to replenish surface O_V_, results in cationic leaching. This in turn leads to surface alterations or even structural collapse, which can affect durability [301, 302]. Therefore, rational optimization of metal–oxygen orbitals is beneficial to solving the stability problem of IBEs. Ge et al. [254] prepared atomically isolated Ir sites dispersed in acid-stabilized MnO_2_ (Ir-MnO_2_) and precisely tuned the covalency of Ir–O bond (Fig. 16a). The Ir–O covalent bond was observed to increase with a clear overlap and local O_L_ activation (Fig. 16b), resulting in the Ir-MnO_2_ catalyst remaining stable for a duration of 650 h in a 0.5 mol L^−1^ H_2_SO_4_ durability testing environment, with no significant cation or anion migration and any notable structural reconstruction occurring during OER. In addition, the regulation of Ir–O covalency by foreign cations with different valence states is different. For example, DFT predictions show that doping IrO_2_ with low-cost metals (Gd, Nd, Pr, etc.) can enhance Ir–O covalency by narrowing the band gap between the band centers of Ir 5d and O 2p, while high-cost (Mo, W) metal substitution can reduce Ir–O covalency (Fig. 16c) [256]. This approach offers a promising avenue for enhancing the stability and efficacy of actual IBEs. By fine-tuning the Ir–O covalency, one can ensure that the dynamic participation of O_L_ occurs exclusively on the catalyst surface, while simultaneously maintaining the overall integrity and structural configuration of the catalyst.

#### Spin-Electron Configuration

While numerous studies on Ir-based catalysts have primarily emphasized the thermodynamic aspects of catalytic processes, spin-dependent reaction kinetics also warrants significant attention [244]. From the thermodynamics perspective, typical OER processes mainly include a variety of intermediates (*OH, *O, *OOH, *OO, etc.). These intermediates are influenced by the spin-dependent electronic configuration of Ir sites and the orbital interactions between the reaction sites [303–306]. Consequently, reconfiguring the spin electron occupation of Ir reaction sites to optimize their bond interactions with intermediates represents an attractive strategy to be used in boosting the stability in acid of IBEs. Liu et al. [255] addressed this challenge by implanting F element in the MnO_2_ matrix as a photocorrosion center, and then performed a cation exchange strategy being light-driven to prepare ordered Ir atomic chains and randomly distributed Ir clusters (IrMnOF@Ir) (Fig. 16d). The entropy difference related to spin-motion, as influenced by atomic disorder, modulates the dynamic reorganization of orbitals between the atomic chains and clusters. The random distribution of Ir clusters results in a breakdown of structural symmetry and a reconfiguration of spin and electron properties. In contrast, ordered Ir atomic chains give rise to spin-dependent electronic reconfiguration and a stronger exchange interaction, due to the transfer of electrons between the e_g_ and t_2g_ states (Fig. 16e). Thus, the bonding between the Ir-atom chains and the MnO_2_ surface is more pronounced, rendering it more resistant to corrosion in acid. Following a long-time test period of 200 h, the material IrMnOF@Ir demonstrated no discernible signs of degradation in its structural morphology or relative chemical composition (Fig. 16f). In general, high spin configurations of oxides with good OER activity are widely used because the d orbitals are partially occupied [305, 307]. However, high spin electron structure is thermodynamically unstable and cannot be ignored [308]. Since the spin occupation is related to the bond order value [57], adjusting the spin orbit to the Fermi level can achieve stronger orbital interaction, so that a high-spin configuration with better OER stability can be constructed. For example, Weng et al. [257] introduced Ir into acid-resistant support MnO_2_ to prepare a layered nanosheet electrocatalyst with a high spin configuration of Mn^3+^ (sl-Mn_1−*x*_Ir_*x*_O_2_). The temperature-dependent susceptibility measurement reveals a high-spin transition based on the excitation of an electron to the d_*z*_^2^ orbital in sl-Mn_1−*x*_Ir_*x*_O_2_ and the orbital position being close to the Fermi level (Fig. 16 g–h). This configuration was found to suppress over-oxidation of the active Ir site and facilitate charge transfer during the "accept-donate" catalytic process. The catalyst retained over 86% of its initial potential after 168 h of durability testing at high current density, demonstrating excellent long-term stability.

Notably, the trade-off between catalyst activity and stability is a critical consideration in electrocatalysis, as optimizing one often compromises the other. High catalytic activity is frequently associated with increased susceptibility to degradation due to structural instability, accelerated dissolution, or unfavorable intermediate adsorption. Conversely, enhancing stability through structural reinforcement or surface modifications may reduce the number of available active sites or alter electronic properties, thereby diminishing catalytic performance. To address this challenge, recent studies have focused on strategies such as defect engineering, heteroatom doping, and electronic structure modulation to achieve an optimal balance. Similarly, incorporating non-precious metal dopants can improve stability while maintaining competitive activity by modifying the d-band center and optimizing adsorption energies. A deeper mechanistic understanding, supported by in situ characterization and theoretical modelling, is essential to designing catalysts that achieve both high activity and long-term stability. Future research should prioritize rational material design strategies that mitigate this trade-off while ensuring industrial applicability.

## Recent Developments of PEMWEs

### Structural Components and Assembly Design

**Fig. 17:**
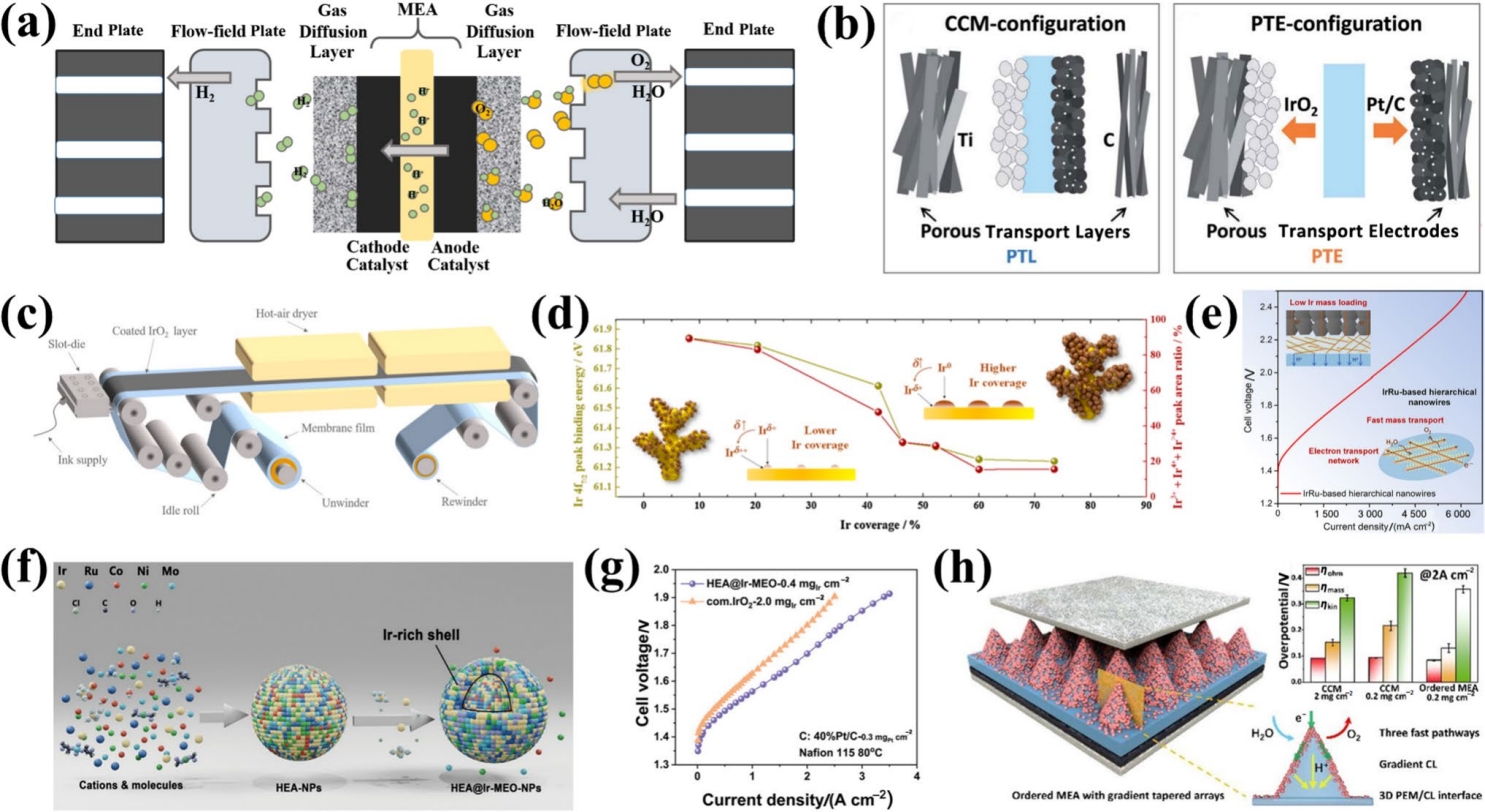
**a** Core component and operating principles of PEMWEs. **b** Schematic of the two configurations of the MEA. Reprinted with permission from Ref. [311]. Copyright © 2017, The Royal Society of Chemistry. **c** Schematic of the process for R2R. Reprinted with permission from Ref. [314]. Copyright © 2020, Elsevier. **d** Ir 4f peak binding energy and the area ratio with the Ir coverage of Ir/Au/CP. Reprinted with permission from Ref. [318]. Copyright © 2021, Elsevier. **e** Schematic and PEMWE performance of the IrRu-based hierarchical catalyst layer. Reprinted with permission from Ref. [319]. Copyright © 2024, Elsevier. **f** Schematic of the prepared HEA and HEA@Ir-MEO. **g** Polarization curves of HEA@Ir-MEO during the PEM electrolyzer. Reprinted with permission from Ref. [320]. Copyright © 2024, Wiley-VCH. **h** Schematic of the ordered MEA based on the anode with the 3D PEM/CL and GTAs. Reprinted with permission from Ref. [321]. Copyright © 2022, American Chemical Society

The MEA is the core component in PEMWEs, which consists of CLs, GDLs and proton exchange membrane (PEM) (Fig. 17a) [309]. During operation, water is introduced to the anode catalyst layer, where it undergoes the oxygen evolution reaction (OER), producing oxygen gas, protons, and electrons. The generated protons are conducted through the PEM to the cathode, where they combine with electrons—supplied via the cathode current collector—to form high-purity hydrogen. Commonly, metal titanium mesh (or plates) or carbon plates are employed as GDLs to facilitate gas diffusion and current collection. Bipolar plates (BPs), which feature flow field structures, help ensure uniform distribution of water and gas, while also supporting efficient electron and heat transfer. The end plate (EP) provides mechanical support and secures all assembly components [310]. Given that the multiphase mass transport occurring in the electrochemical reaction process is a key factor in MEA, it becomes necessary to optimize the structural components of MEA to enhance the efficiency and cost-effectiveness of PEMWEs, which are the final products of the electrochemical reaction process.

Two main MEA fabrication strategies are commonly employed for the fabrication of MEA (Fig. 17b) [311]. The first of these is the CCM-type MEA, which is produced by spraying a proton-conductive ionomer solution with a catalyst deposited directly on both sides of the membrane. Alternatively, the CCM-type MEA can be manufactured by anodic bonding, a process known as the decal transfer process, which results in a well-connected interface and mechanical stability between the catalyst and the PEM, leading to reduced ohmic losses. Consequently, the preparation of uniform coating films is essential for the efficacious reduction of precious metal loading and the objective is to enhance the availability of CCM-type MEA. Compared to the commonly used decal transfer method with hot pressing, magnetron sputtering and ultrasonic spraying technology [312, 313], the choice of more advanced coating methods (such as roll-to-roll (R2R) (Fig. 17c) [314], atomic layer deposition (ALD) [315], and reactive spray deposition technology (RSDT) [316]) will increase the interfacial contact and reduce the contact resistance between PEM, CLs and catalyst loading, thus effectively improving the performance of PEMWEs. The second electrode configuration is a porous transport electrode (PTE)-type MEA. The catalyst is applied to the PTL of the cathode and anode by either spraying or printing, and subsequently combined with the PEM by hot-pressing, which is simple to manufacture and avoids PEM damage. Nevertheless, the PTE-type MEA exhibits a lack of connectivity between the catalyst and the PEM, which results in elevated proton transfer resistance, diminished catalyst utilization, and compromised mechanical stability. In addition, the fabrication of PTE using powder-based catalysts requires an additional coating process using a polymeric binder, which can reduce the electrical conductivity and block the active sites of IBEs. Consequently, the fabrication of IBEs directly on the substrate through the preparation of a PTE represents a viable approach to addressing this issue [108, 317]. Kim et al. [318] reported a novel Ir-based PTE that precisely controls Ir metal loading deposited on dendritic Au/carbon paper by deposition pulses. The engineered Ir-Au interface modulated the electronic structure of Ir, and the high Ir surface coverage provided abundant OER-active sites and enhanced stability, significantly reducing both ohmic and mass transport losses in the PEMWE system (Fig. 17d). Therefore, reasonable MEA structural components and assembly design should be considered for improving the performance of PEMWEs, but the performance of PEMWEs depends heavily on the OER electrocatalyst.

### Applications and Performance

In the 1970s, PEMWEs could achieve 1.88 V at 1 A cm^−2^ and 2.24 V at 2 A cm^−2^ with no significant degradation over 15 000 h of operation, respectively [20]. With the development of Ir-based materials, PEMWE performance requirements have attracted extensive attention and changes. For example, the DOE has set the following technical goals for PEMWE stacks by 2025 [322]: operating current density greater than 3 A cm^−2^ at 1.9 V with the Ir mass loading less than 0.5 mg cm^−2^, and lifetime greater than 80 000 h with the degradation rate less than 2.25 mV kh^−1^. To achieve the goal of large-scale commercial application, the activity and stability of IBEs in real PEMWEs should be used as evaluation criteria. The performance assessment of PEMWE is contingent upon the evaluation of mass transport and charge transport behaviors at high current densities [323, 324]. To get a direct understanding of the real activity and stability in PEMWEs, we summarize the current PEMWE performance of advanced IBEs as shown in Table 5.

**Table 5:** The current PEMWE performances of advanced IBEs

Anode catalysts	Loading/ (mg cm^−2^)	Activity/(V @ A cm^−2^)	Stability/ (h @ A cm^−2^)	Temperature °C	Active area/cm^2^	References
DNP-IrNi	0.67	2@6.5	100@2	90	1	[108]
30Ir/Au/CP	0.008	1.75@2	20@0.5	90	1	[318]
GB-Ta_0.1_Tm_0.1_Ir_0.8_O_2−*δ*_	0.2	1.766@1	500@1.5	50	4	[132]
IrRu HNWs	1	1.84@3	240@2	80	4	[319]
HEA@Ir-MEO	0.4	1.85@3	500@1	80	4	[320]
Ir/Nb_2_O_5−*x*_	3	1.839@3	2 000@2	80	3.4	[251]
MEA-GTAs-T1	0.2	1.801@2	300@1	65	1	[321]
Sr_2_CaIrO_6_	0.4	1.81@2	450@2	80	4	[119]
IrO_*x*_·*n*H_2_O	2	1.77@1	600@1	60	25	[246]
IrO_2_ nano-textile	0.2	< 2@7	1 400@1	80	~ 0.2	[326]
Ir@WO_*x*_NRs	0.14	2@2.2	1 030@0.5	80	4	[327]

Data are extracted from different resources, thus with different significant digits

Most experimental studies employ an operating temperature of 80 °C. While temperatures above 100 °C facilitate rapid reaction kinetics and enhance the performance of PEMWEs, they are more challenging for device components such as membrane dehydration and necessitate higher pressures for the device [325]. Additionally, the electrolyte flow rate affects proton and charge transfer resistance. Hence, optimizing operating conditions is crucial for achieving practical and reproducible PEMWE performance. On the other hand, the understanding of the activity and stability mechanisms derived from the three-electrode cell test system may also be critical in the PEMWE device and reduction of the loading mass of the IBEs layer (< 2 mg cm^−2^). Therefore, the microstructure regulation of CLs is of great significance for the practical application of PEMWEs. Guo et al. [319] developed a 3D-CL structure comprising layered sub-nanosheets of Ru and Ir (RuIr-HNWs) integrated onto nanowires. These nanowires are connected to a fast electron transport network and mass transfer channel (Fig. 17e), which operate under a relatively low Ir load, thus facilitating electron transfer at the interface. In PEMWE applications, RuIr-HNWs can achieve a 2.44 V battery voltage at 6.0 A cm^−2^ with a low mass loading (Ir) of 0.35 mg cm^−2^ and operate stably for more than 200 h at 2.0 A cm^−2^. Furthermore, the utilization of less costly transition metals not only serves to reduce the expenditure incurred on Ir in CLs, but also produces a variety of effects with unlimited performance adjustability. Cheng et al. [320] successfully prepared sub-2 nm HEA nanoparticles with the Ir-rich medium-entropy shell and high-entropy core (HEA@Ir-MEO) by introducing a variety of transition metals combined with a galvanic replacement strategy (Fig. 17f). The multiple transition metals doped high-entropy core generate a "cocktail effect" that effectively changes the electronic structure of Ir, and the Ir-rich MEO shell inhibits the violent structural evolution of the transition metal on the OER for ensuring structural stability. The small size and the use of transition metals allow HEA@Ir-MEO catalysts to provide excellent PEMWE performances with (1.85 V/3.0 A cm^−2^@80 °C) (Fig. 17g), and cost effectiveness with US$0.88 per kg of H_2_, which is below the target set by DOE of US$2 per kg of H_2_. The optimization of the three-phase boundary and the improvement of the fast mass transfer pathway at the membrane/CLs interface are essential to enhance the performance of PEMWEs and reduce the Ir usage. Yang et al. [321] reported an ordered MEA with 3D PEM/CL interfacial and gradient tapered arrays (GTA) by nanoimprinting (Fig. 17h). The ordered arrays and gradient 3D PEM/CLs interface reduce the mass transfer and contact resistance. In comparison to a conventional MEA, GTA can operate at 1.801 V at 2 A cm^−2^, exhibiting the stability at 1 A cm^−2^ for 300 h with an Ir loading of 0.2 mg cm^−2^.

In practical PEMWE systems, degradation mechanisms extend beyond half-cell observations, driven by complex operational challenges. High current densities (> 1 A cm^−2^) accelerate iridium dissolution via repeated redox cycling (Ir^3+^/Ir^4+^/Ir^6+^), leading to Ir migration and redeposition on the cathode, which reduces catalyst utilization. Simultaneously, Fenton reactions involving metal impurities degrade Nafion membranes and ionomers through peroxide and radical formation, impairing proton conductivity and system longevity. Catalyst-support interactions further complicate stability: carbon supports corrode under acidic OER conditions, causing catalyst detachment, while metal oxide alternatives (e.g., TiO_2_, SnO_2_) introduce interfacial resistance that hinders charge transfer. Mechanical and thermal stresses can induce catalyst layer cracking, particle agglomeration, and interfacial delamination due to differential thermal expansion between components, such as temperature fluctuations during start-stop cycling or intermittent operation. To bridge the gap between laboratory-scale innovations and industrial deployment, future research should emphasize the development of: (i) dissolution-resistant IBEs, (ii) corrosion-resistant, conductive catalyst supports, (iii) robust MEA architectures that minimize interfacial resistance, and (iv) accelerated stress testing under realistic PEMWE conditions. Such strategies are essential to mitigate performance degradation and realize long-term, commercially viable hydrogen production.

## Non-Precious Metal-Based Catalysts

Given the natural scarcity and high cost of precious metals, the development of alternative materials based on abundant and inexpensive non-precious metals, such as cobalt (averaging at US$19 lb^−1^), iron (US$0.26 lb^−1^) and manganese (US$1.5 lb^−1^) [328]. It is also particularly important to use low-cost, non-precious metal-based materials with high earth abundance, activity and stability to accelerate the rate of commercial application of PEMWEs. While non-precious metal catalysts have demonstrated excellent performance in OER under alkaline conditions [329], they tend to suffer from activity and stability problems in acidic environments [330]. Despite advances in materials and strategies to improve the performance of non-precious metal-based catalysts in acidic OERs, there are inherent challenges in acidic media due to the rapid dissolution of the metal species, the oxidative degradation of the carriers, and slow kinetics due to strong adsorbate interactions. Therefore, to explore design guidelines for achieving high stability and excellent activity, we first summarized promising non-precious metal materials (Table 6) and analyzed key design strategies for enhancing the activity and stability of non-precious metal catalysts in acid, which can be used to screen for the development of new non-precious metal catalysts. Furthermore, we compiled representative performance data from PEMWE systems employing non-precious metal-based catalysts to provide an overview of their practical viability and to guide future material development efforts.

**Table 6:** Performances of non-precious electrocatalysts in acidic media

Catalysts	Electrolyte	Overpotential /(mV @ 10 mA cm^−2^)	Stability/(h @ mA cm^−2^)	References
La, Mn-cobalt spinel	0.1 mol L^−1^ HClO_4_	353	360@10	[331]
Co_2_TiO_4_	0.5 mol L^−1^ H_2_SO_4_	513	10@10	[332]
Co_1.8_Ga_1.2_O_4_	0.5 mol L^−1^ H_2_SO_4_	310	200@200	[333]
W-Co_3_O_4_	0.5 mol L^−1^ H_2_SO_4_	251	250@10	[334]
Er-Co_3_O_4_	0.5 mol L^−1^ H_2_SO_4_	321	250@10	[335]
γ-MnO_2_	1 mol L^−1^ H_2_SO_4_	489	8 000@10	[336]
Mn_7.5_O_10_Br_3_	0.5 mol L^−1^ H_2_SO_4_	295	500@10	[337]
porous Fe_5_Si_3_	50 g L^−1^ H_2_SO_4_	735	/	[338]
V-CoP_2_	0.5 mol L^−1^ H_2_SO_4_	91	20@10	[339]
NiAl_*δ*_P	0.5 mol L^−1^ H_2_SO_4_	256	7@10	[340]
CoMoNiS-NF-3	0.5 mol L^−1^ H_2_SO_4_	255	80@20	[341]
Ni_2_Ta	0.5 mol L^−1^ H_2_SO_4_	570	66@10	[342]
TiB_2_	1.0 mol L^−1^ HClO_4_	560	10@10	[343]
COOH-MWNTs	0.5 mol L^−1^ H_2_SO_4_	265	10@10	[344]
FeN_*x*_/NF/EG	0.5 mol L^−1^ H_2_SO_4_	294	24@20	[345]
N-WC nanoarray	0.5 mol L^−1^ H_2_SO_4_	~ 230	60@10	[346]
MoSe_2_-2:Mo_2_C	0.5 mol L^−1^ H_2_SO_4_	197	/	[347]
TiTaF_*x*_C_2_ NP/rGO	1.0 mol L^−1^ HClO_4_	240	40@30	[348]
Co_SA_-MoCeO_*x*_@BCT	0.5 mol L^−1^ H_2_SO_4_	239	60@10	[349]
Co_3−*x*_Ba_*x*_O_4_	0.5 mol L^−1^ H_2_SO_4_	278	110@10	[350]
Co_3_O_4_@C/GPO	1 mol L^−1^ H_2_SO_4_	360	40@10	[351]
Co–Co DASs/ZCC	0.5 mol L^−1^ H_2_SO_4_	155	40@10	[352]
Co_3_O_4_/CeO_2_	0.5 mol L^−1^ H_2_SO_4_	423	50@10	[353]
Co_2_MnO_4_	pH 1.0 H_2_SO_4_	395	320@100	[354]
Co_3_O_4−*x*_F_*x*_	0.5 mol L^−1^ H_2_SO_4_	349	120@100	[355]

Data are extracted from different resources, thus with different significant digits

### Materials

#### Transition Metal-Based Catalysts

**Fig. 18:**
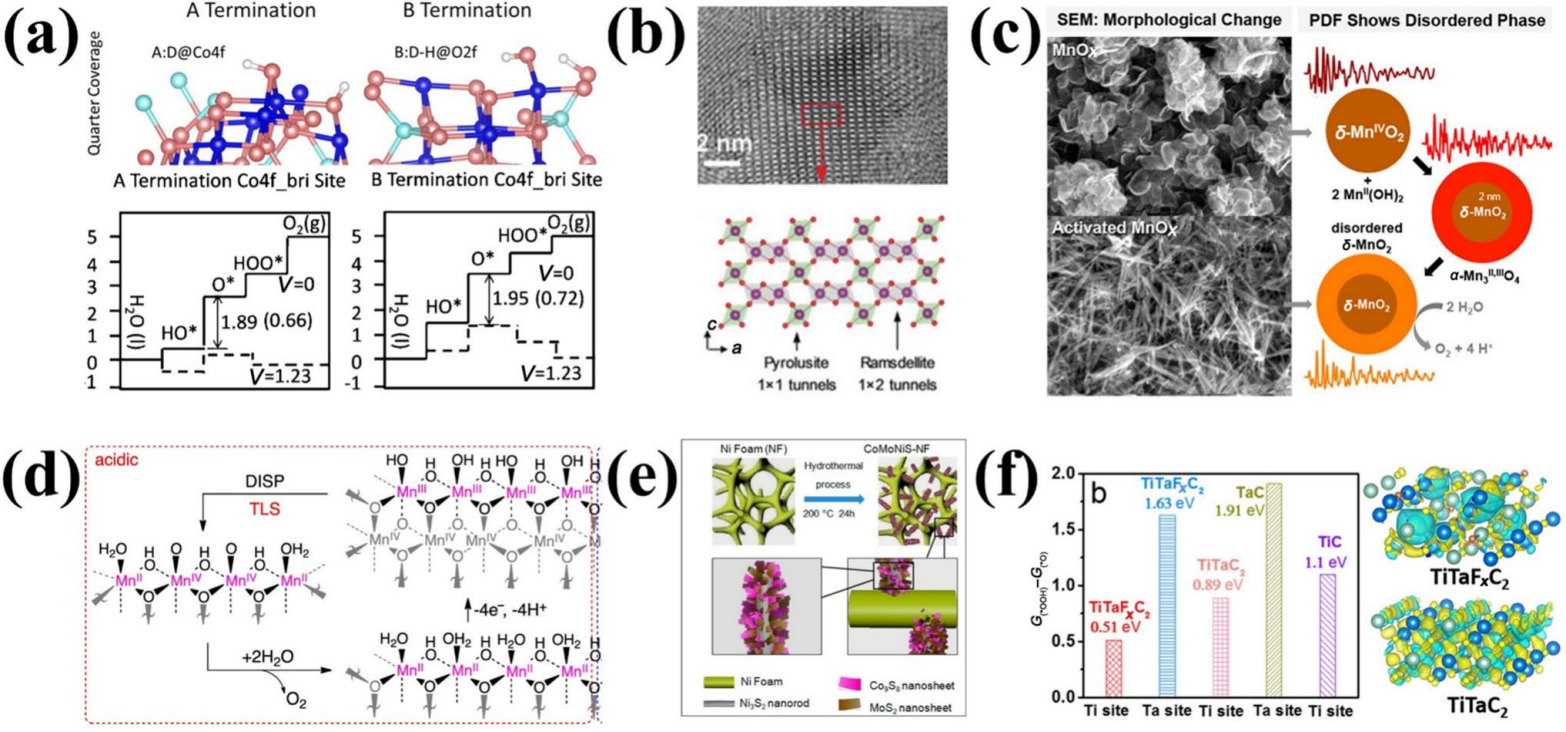
**a** Structure of the stablest configurations of adsorbed water on the terminations and free-energy diagram. Reprinted with permission from Ref. [358]. Copyright © 2012, American Chemical Society. **b** TEM and the Schematic diagram of γ-MnO_2_ showing an intergrowth structure of pyrolusite and ramsdellite matrices. Reprinted with permission from Ref. [336]. Copyright © 2019, Wiley-VCH Verlag GmbH & Co. KGaA, Weinheim. **c** Schematic for the structure of nanosized MnO_*x*_ domains during activation. Reprinted with permission from Ref. [359]. Copyright © 2015, American Chemical Society. **d** Proposed mechanism for the OER on MnO_*x*_ in acidic environments. Reprinted with permission from Ref. [360]. Copyright © 2014, American Chemical Society. **e** Schematic illustration of the synthesis and growth of hierarchical CoMoNiS-NF-xy composites. Reprinted with permission from Ref. [341]. Copyright © 2019, American Chemical Society. **f** Histogram of energy barriers and the charge density distributions of TiTaF_*x*_C_2_. Reprinted with permission from Ref. [348]. Copyright © 2022, American Chemical Society

Transition metal-based compounds, including oxides, chalcogenides, phosphides, and nitrides, have gained prominence as viable substitutes for noble-metal catalysts in acidic OER. Their appeal lies in their tuneable electronic structures, flexible oxidation states, and rich redox chemistry. Among these, cobalt-, manganese-, and iron-based oxides stand out as extensively studied candidates, demonstrating considerable catalytic promise in acidic media through their structurally optimized crystalline frameworks and oxidation state dynamics [356]. For instance, among cobalt oxides (including CoO, Co_2_O_3_, and Co_3_O_4_), the activity of spinel Co_3_O_4_ is enhanced when a Co^2+^ is in the tetrahedral site (Co^2+^_Td_) to be the active center for OER [357]. The DFT predicts that Co_3_O_4_ exhibits higher activity at Co_4f_ bridge sites (Fig. 18a) [358], but metal dissolution under high oxidative potentials remains a critical challenge. Doping strategies such as La and Mn co-doping [331], Ga [333], W [334], Er [335], and Ti [332] in cobalt spinel oxides have been shown to suppress dissolution by enhancing structural integrity and electronic conductivity.

Manganese oxides exhibit structural diversity across multiple polymorphs—including β-MnO_2_ (pyrolusite), R-MnO_2_ (ramsdellite), γ-MnO_2_ (intergrowth structures), and δ-MnO_2_ (birnessite-like layered phases)—alongside redox adaptability and potential dynamic self-repair capabilities. These properties result in stability-activity trade-offs governed by crystallographic phases, surface reconstruction processes, and operational parameters. Notably, the γ-MnO_2_ phase, characterized by a hybrid α/β-MnO_2_ framework (Fig. 18b) [336], demonstrates exceptional acidic durability for maintaining OER activity at 10 mA cm^−2^ for over 8 000 h. In contrast, δ-MnO_2_ undergoes phase transitions during electrochemical activation to form hausmannite-like intermediates (α-Mn_3_O_4_) (Fig. 18c), followed by anodic conditioning to yield disordered active surfaces with enhanced OER kinetics [359]. In addition, a distinctive feature of manganese oxides is their capacity for in situ regeneration during OER. Electrodeposited MnO_*x*_ films dissolve under acidic conditions but simultaneously redeposit via anodic oxidation of Mn^2+^ (Fig. 18d), maintaining a dynamic equilibrium that ensures functional stability [360, 361]. This self-repair process is pH-dependent, with optimal stability observed near pH 2–2.5, where MnO_*x*_ deposition kinetics overlap with OER activity. The addition of halogens (such as Br^−^, Cl^−^) to the manganese oxide lattice can significantly improve activity and durability [337].

Iron oxides emerge as cost-effective OER catalysts due to their intrinsic redox adaptability and natural abundance, though their susceptibility to acidic dissolution necessitates strategic material engineering. Fe oxides exist in crystallographic phase diversity, including hematite (α-Fe_2_O_3_), magnetite (Fe_3_O_4_), and maghemite (γ-Fe_2_O_3_), which dictates distinct catalytic performance profiles. Hybrid γ-Fe_2_O_3_/α-Fe_2_O_3_ systems demonstrate synergistic stabilization, where the γ-phase's Fe vacancy-rich structure enhances interfacial water activation and reaction kinetics, while the α-phase matrix confers corrosion resistance through robust Fe–O bonding networks [362]. Transition metal doping (Ti [363], Si [338], Co [362]) further optimizes these systems by inducing ligand field distortions that improve charge transfer kinetics, elevate active site density, and inhibit cationic leaching under prolonged polarization.

Incorporating non-metallic elements like phosphorus and sulfur can significantly boost the catalytic performance of transition metal systems. Metal phosphides (e.g., Ni_2_P [364], V-CoP_2_ [339], NiAl_*δ*_P [340]) demonstrate metallic conductivity and surface adaptability, where phosphorus atoms act as electron donors to downshift the d-band centre of metal sites. This electronic modulation weakens oxygen intermediate adsorption energies, enabling efficient acidic OER kinetics. Similarly, S-containing systems of the MoS_2_/Co_9_S_8_/Ni_3_S_2_/Ni-foam heterostructure exhibit interfacial synergy in acid (Fig. 18e) [341], facilitated by Co_9_S_8_-MoS_2_ interfacial electron transfer that optimizes adsorbate binding configurations. Beyond chalcogenides, refractory intermetallics like Ni_2_Ta [342] and titanium diboride (TiB_2_) [343] demonstrate corrosion-resistant OER operation with moderate overpotentials (560–570 mV), attributed to their strong covalent bonding networks inhibiting metal dissolution under harsh acidic conditions.

#### Carbon-Based Catalyst

Carbon-based materials offer significant advantages for acidic OER applications through their exceptional electrical conductivity, structural tunability, and mass transport benefits. However, their operational deployment requires overcoming inherent thermodynamic instability under combined acidic/oxidative conditions through advanced functionalization approaches.

The incorporation of heteroatoms (e.g., N, O, S, and P) into carbon matrices disrupts the homogeneous sp^2^ hybridization, creating charge density gradients that enhance catalytic activity. Systems such as carboxylated multi-walled carbon nanotubes (COOH–MWNTs) demonstrate improved OER kinetics by stabilizing reactive intermediates [344], which is attributed to dynamic lactone formation and hydrolysis cycles that regenerate active sites. Partial oxidation introduces protective oxygen functionalities (hydroxyl, epoxy, carbonyl), though excessive surface oxidation accelerates CO/CO_2_ evolution at industrial current densities [365, 366]. Theoretical studies identify the phenanthrenequinone-like configurations as optimal active centers through favorable OH adsorption energetics [367]. Moreover, atomically dispersed transition metals (Fe, Co) in nitrogen-doped carbon matrices synergize single-atom catalysis with matrix stabilization. FeN_4_ sites embedded in N-CNTs exhibit an overpotential of 294 mV with suppressed Fe leaching via strong N-coordination, while simultaneously optimizing oxygen adsorption energetics [345]. In addition, metal carbides (WC [346], Mo_2_C [347], and TiTaF_*x*_C_2_ [348]) leverage Pt-like electronic structures and covalent bonding for acid durability. Fluorine-doped TiTaF_*x*_C_2_ demonstrates exceptional performance (490 mV @ 100 mA cm^−2^, 40 h stability), where DFT reveals F-induced charge redistribution lowers the energy barrier for OOH formation (Fig. 18f).

### Key Design Strategies

**Fig. 19:**
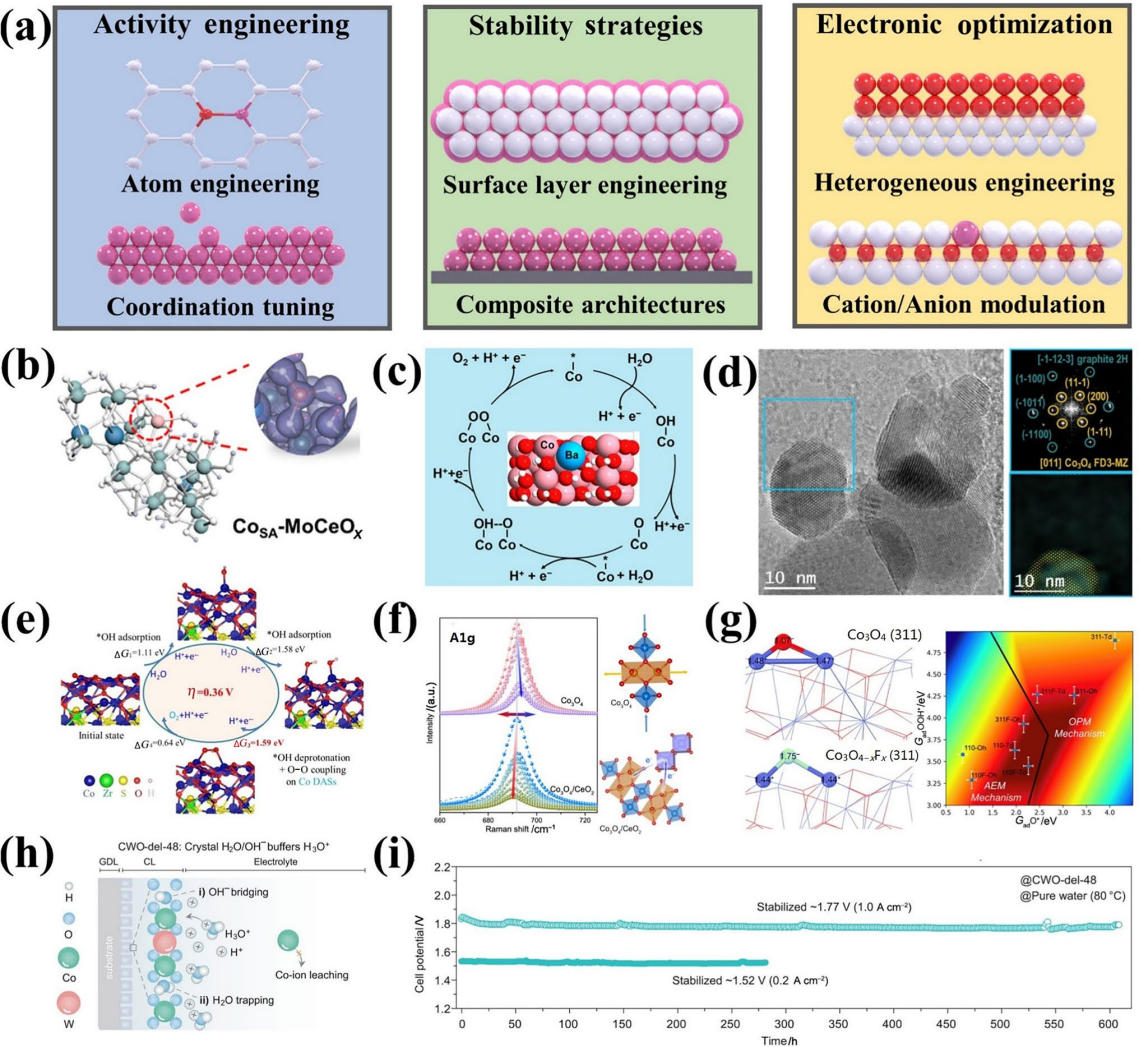
**a** Key design strategies for enhancing activity and stability. **b** Calculation models and the electronic distributions of Co_SA_-MoCeO_*x*_. Reprinted with permission from Ref. [349]. Copyright © 2024, The Royal Society of Chemistry. **c** OPM mechanism for catalysts in acidic electrolytes. Reprinted with permission from Ref. [350]. Copyright © 2023, American Chemical Society. **d** Co_3_O_4_ corresponding lattice fringes are in yellow and the graphite layer ones in turquoise. Reprinted with permission from Ref. [351]. Copyright © 2022, The Author(s). **e** Proposed 4e-mechanism of OER following the O − O coupling mechanism. Reprinted with permission from Ref. [352]. Copyright © 2022, Wiley-VCH GmbH. **f** In situ Raman A1g peaks of Co_3_O_4_/CeO_2_ and the local bonding environment changes. Reprinted with permission from Ref. [353]. Copyright © 2021, The Author(s). **g** Bader charge analysis and the 2D activity map for Co_3_O_4−*x*_F_*x*_. Reprinted with permission from Ref. [355]. Copyright © 2024, The Royal Society of Chemistry. **h** Depiction of delaminated cobalt catalysts (CWO-del-48), and **i** chronopotentiometry stability test. Reprinted with permission from Ref. [369]. Copyright © 2024, The Author(s)

The sluggish oxygen evolution kinetics and intrinsic material instability of nonprecious acid-stable OER catalysts present dual challenges of low energy conversion efficiency and accelerated cost escalation in PEMWEs. To address these limitations, some key design strategies emerge as critical pathways toward industrial implementation, including atomic site engineering, coordination tuning, surface layer engineering, composite architectures, heterogeneous engineering, and cation/anion modulation (Fig. 19a).

#### Active Site Engineering

Atomic-scale engineering of catalytic sites enables precise optimization of oxygen evolution reaction kinetics. Li et al. [349] demonstrated this through strategic integration of single cobalt atoms into amorphous Mo-Ce oxide matrices (CoSA-MoCeO_*x*_@BCT), activating a bimetallic LOM pathway. The catalyst achieves a low overpotential of 239 mV at 10 mA cm^−2^ with 60 h stability in PEMWEs. The DFT reveals that strong Co-O-Mo/Ce interfacial interactions reduce the adsorption energy disparity between adjacent metal sites (Fig. 19b), while simultaneously elevating Mo-site oxygen vacancy formation energy. This dual electronic modulation mechanism enhances both catalytic activity and acid stability through optimized lattice oxygen participation and suppressed metal dissolution.

Tuning the coordination environment offers another precise means to modulate electrocatalytic pathways at the atomic scale. A representative study by Sargent’s group demonstrates this principle through doping Ba cations into the Co_3_O_4_ framework for tuning the coordination of Co [350], which shortens the Co–Co interatomic distance and enhances surface hydroxyl (OH) coverage. Co_3−*x*_Ba_*x*_O_4_ achieves a low overpotential of 278 mV at 10 mA cm^−2^ in 0.5 mol L^−1^ H_2_SO_4_, along with 110 h stability. The DFT reveals that Ba doping stabilizes high-valence Co sites and reduces surface free energy, promoting OPM intermediates such as μ-OO bridges (Fig. 19c). In situ spectroscopic analyses confirm this pathway evolution, showing direct O–O coupling and suppressed Co dissolution.

#### Stability Enhancement Strategies

Mitigating catalyst degradation under acidic and oxidative environments demands innovative stabilization approaches. Surface engineering typically employs surface coatings and self-repair to increase stability in acid. A carbon-protected Co_3_O_4_ nanocomposite (Co_3_O_4_@C) embedded in a hydrophobic graphite-paraffin matrix (Fig. 19d) [351], combined with a hydrophobic binder to mitigate corrosion in 1 mol L^−1^ H_2_SO_4_, achieves an overpotential of 360 mV at 10 mA cm^−2^ and sustains 40 h operation without degradation. The carbon matrix restricts Co leaching and improves conductivity, hydrophobic interactions suppress acidic corrosion, and optimized Co^2+^/Co^3+^ ratios enhance OH adsorption. While carbon-based protection suffers from inherent oxidative instability, acid-resistant oxide coatings present a viable alternative. A representative advancement employs atomic-layer-deposited amorphous TiO_2_ (4.4 nm) on Co_3_O_4_ electrocatalysts [368], achieving 80 h operational stability in H_2_SO_4_. The enhanced stability arises from TiO_2_ coatings at optimal thickness, which expose active Co_3_O_4_ sites while mitigating acid-induced dissolution.

Composite architectures, achieved via heterostructure integration and atomic doping, have also been demonstrated to enhance acidic OER durability. Li et al. [352] developed Zr-incorporated Co_9_S_8_/Co_3_O_4_ heterocatalysts featuring engineered Co-Co dinuclear sites, achieving exceptional stability (500 h @ 100 mA cm^−2^) and activity (*η* = 155 mV @ 10 mA cm^−2^) in acid. These catalysts deliver a record mass current density of 120 000 mA g^−1^ in PEMWEs. Zr doping promotes the formation of elongated Co–O bonds and introduces S-Co-O non-homogeneous grain boundary interfaces, effectively addressing instability under acidic conditions. The DFT calculations reveal that Zr plays a dual role by stabilizing high-valence Co species through electron redistribution and enabling dual-site adsorption kinetics that circumvents conventional scaling relations (Fig. 19e).

#### Electronic Structure Optimization

Heterogeneous engineering is critical for tuning electronic configurations for balancing activity and durability. Jin et al. [353] integrated nanocrystalline CeO_2_ to modulate Co_3_O_4_'s redox properties and local bonding environment for addressing the Co_3_O_4_ surface reconstruction into stabilized Co^IV^ intermediates. Advanced characterizations (XAS, in situ Raman) reveal that CeO_2_ disrupts dimeric Co^IV^ formation (Fig. 19f), enabling easier oxidation of Co^III^ to active Co^IV^ species and suppressing structural reorganization, achieving a low overpotential of 347 mV at 10 mA cm^−2^ on carbon paper and sustaining 100 h operation in 0.5 mol L^−1^ H_2_SO_4_. CeO_2_ induces electronic redistribution that accelerates Co oxidation kinetics while preserving structural integrity, altering the rate-determining step (e.g., OOH dissociation) and reducing charge accumulation.

Cation modulation further enhances catalyst stability by stabilizing metal valence states [370], strengthening the metal–oxygen bond and stabilizing the surface structure using intermediate species [331, 350, 354], thus improving its stability in acidic solutions. For example, the enhancement of cobalt spinel oxide stability (> 1 500 h at 200 mA cm^−2^ in pH 1) while maintaining high activity is achieved by incorporating Mn into Co_3_O_4_ to form Co_2_MnO_4_ [354]. The improved performance stems from optimized binding energies of OER intermediates and suppressed dissolution thermodynamics due to the stronger Mn–O bonding based on electron transfer from Mn to O. To address sluggish kinetics for Co_3_O_4_ surface reorganization into inactive Co^IV^ = O species during OER, Wu et al. [355] proposed a fluorination strategy to reconstruct F-Co-O active sites, modulating the Co pre-oxidation process. The optimized catalyst achieves a low overpotential of 349 mV at 10 mA cm^−2^ and sustains 120 h operation at 100 mA cm^−2^ in acid. Performance enhancement arises from F regulating the local coordination environment, suppressing Co^IV^ = O formation and promoting stable F-Co^III^-OH active centers with favorable electron redistribution (Fig. 19g), altered rate-determining steps and switched OER pathways (AEM/OPM), thereby accelerating reaction kinetics.

### Performance of Non-Precious Catalysts in PEMWEs

The advancement of non-precious catalysts for PEMWEs has garnered significant attention, yet their practical integration into MEAs remains underexplored. While limited in scope, MEA-based evaluations are critical for assessing the real-world applicability of these materials, as summarized in Table 7. Current research predominantly focuses on manganese- and cobalt-based oxides, which exhibit varying degrees of electrochemical performance under operational conditions.

**Table 7:** Current PEMWE performances of advanced IBEs

Anode catalysts	Activity/(V @ A cm^−2^)	Stability/(h @ A cm^−2^)	Temperature °C	Active area/cm^2^	Reference
La, Mn-cobalt spinel	3@4	90@0.3	80	5	[331]
Co_1.8_Ga_1.2_O_4_	~ 1.8@0.5	450@0.2	80	4	[333]
W-Co_3_O_4_	2.04@2	240@1	50	4	[334]
γ-MnO_2_	~ 1.8@0.25	350@10	25	4	[336]
Mn_7.5_O_10_Br_3_	/	300@0.1	50	4	[337]
Co_SA_-MoCeO_*x*_@BCT	/	60@0.1	25	5	[349]
Co-Co DASs/ZCC	2.14@3	50@0.05	80	/	[352]

Data are extracted from different resources, thus with different significant digits

Structural engineering of manganese oxides enables significant improvements in stability for oxygen evolution catalysis. While conventional γ-MnO_2_ demonstrates exceptional longevity (> 8 000 h) in liquid electrolytes [336], its PEMWEs applicability is limited by rapid degradation at high current densities (< 100 mA cm^−2^). Thermal optimization (150–450 °C) addresses this through crystallographic control, with 450 °C-annealed γ-MnO_2_ achieving 94% planar oxygen coordination and sustained operation for 1 000 h @ 200 mA cm^−2^ via strengthened Mn–O bonds [371]. Anion-substituted manganese oxides, such as Mn_7.5_O_10_Br_3_ [337], further showcase stable operation for 300 h at 100 mA cm^−2^, highlighting the efficacy of compositional tuning.

Cobalt-based catalysts have also achieved notable operational milestones in PEMWEs through strategic doping and structural design. For example, La/Mn-codoped Co_3_O_4_ spinel demonstrates 100 h stability at 1.65 V with ultralow degradation (20 μA cm^−2^ h^−1^) [331], where La stabilizes the cubic framework while Mn optimizes conductivity. High-entropy chalcogenides like CoFeNiMoWTe sustain 100 h at 1 000 mA cm^−2^ through entropy-stabilized atomic configurations and Te-mediated oxidation state modulation [372]. Delaminated CoWO_4_ exhibits exceptional acid resistance via WO_4_^2−^/H_2_O/OH^−^ ion exchange (Fig. 19h), maintaining 600 h operation at 1 000 mA cm^−2^ (Fig. 19i) [369]. While commercial PEM systems predominantly employ powdered catalysts (e.g., La/Mn-Co_3_O_4_, CoWO_4_), emerging architectures like electrodeposited γ-MnO_2_ on Pt/Ti substrates (94% planar O) demonstrate enhanced durability through improved interfacial contact [371]. Despite these advances, achieving catalytic efficiencies comparable to noble metal benchmarks remains a significant challenge for non-precious catalysts.

## Conclusions and Future Research Directions

**Fig. 20:**
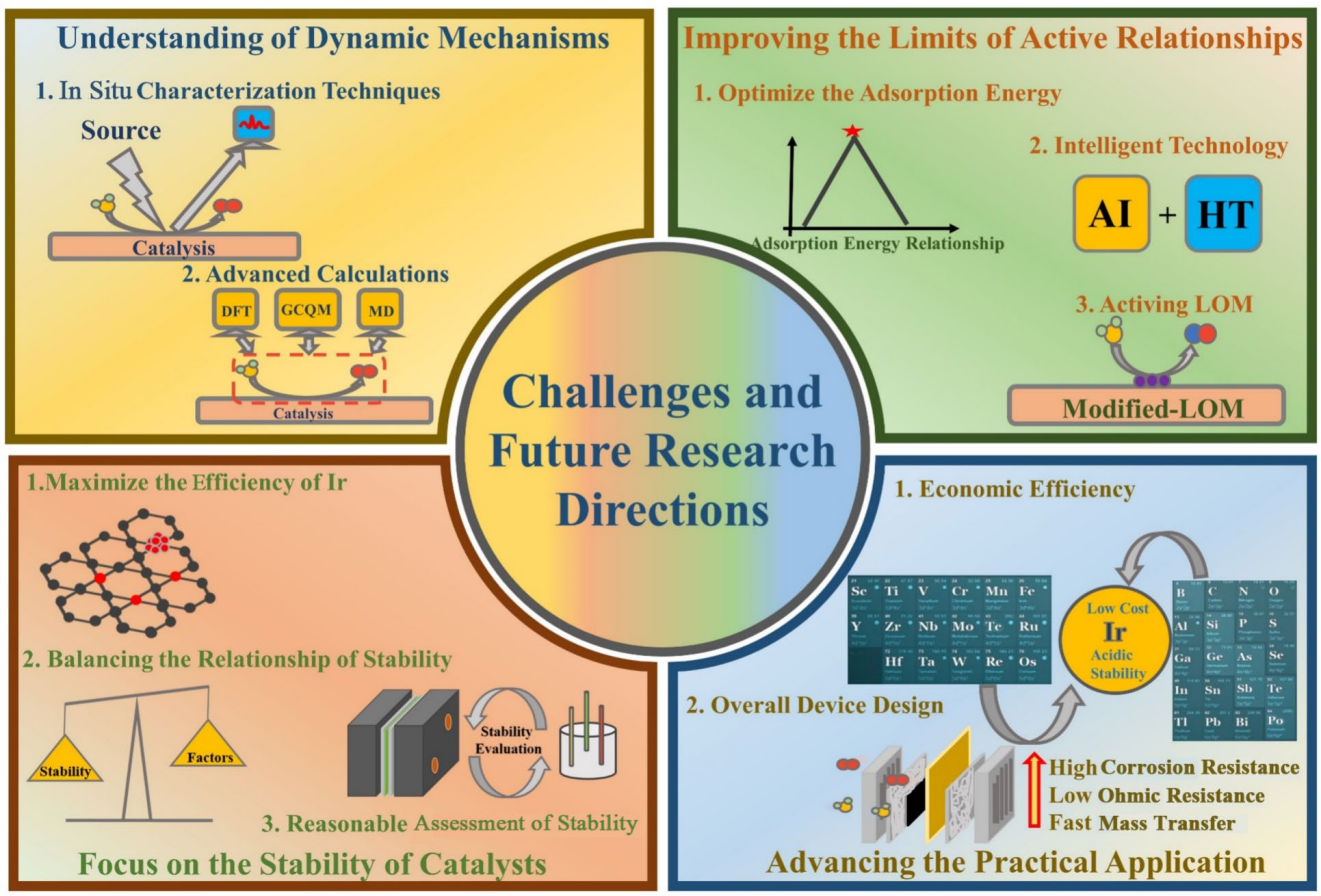
Schematic illustration of challenges and future research directions of IBEs

Improving the activity and stability of OER electrocatalysts can yield significant economic benefits and meet the rigorous demands of industrial applications. This report reviews the OER mechanism of IBEs in an acidic environment to elucidate the origins of catalytic activity and dissolution of Ir species. Additionally, strategies to promote the development of IBEs and non-precious metal catalysts, focusing on improving their activity and stability, are summarized. Furthermore, the design of PEMWEs and practical applications are discussed. Despite considerable progress in enhancing the activity and stability, challenges remain for deployment in industrial-scale PEMWEs applications. Therefore, this report emphasizes the need to address these challenges and outlines future research directions (Fig. 20).

### Understanding Dynamic Catalytic Mechanisms

Recent experimental findings have questioned conventional mechanistic models of electrochemical processes, which often assume well-defined electrode-electrolyte interfaces. These classical frameworks fail to comprehensively explain the origins of observed high activity and stability. Notably, the active sites involved in OER may undergo reversible structural transformations because of the three-phase reaction (involving solid, liquid, and gas), four-electron transfer, and multiple intermediates in the acidic OER process. Moreover, the catalysts may experience drastic reconfiguration under the harsh corrosive media of OER and high operating potentials, and the dynamic surface chemistry hinders the identification of the true active phase and the insight into the reaction mechanism. Integrating advanced operando characterization techniques—such as operando XAS, Raman spectroscopy, and differential electrochemical mass spectrometry (DEMS)—can provide real-time insights into active site evolution, lattice oxygen participation, and degradation mechanisms, thereby enabling more rational catalyst design.

In addition, the experimental results are closely combined with the computational predictions derived from DFT calculations to better reveal the reaction dynamics of OER by estimating the thermodynamics of key intermediates on the catalyst surface. While various strategies have been established for effectively developing efficient electrocatalysts, further research and validation are needed for new strategies, reflecting the dynamic surface properties of catalytic materials. Meanwhile, it is important to note that different reaction mechanisms predicted by DFT calculations may result in the same overpotential for the acidic OER. Factors such as parameter sensitivity, surface adsorption characteristics, and hydrogen-bonding interactions among intermediates and solvent molecules significantly affect calculation accuracy. Consequently, more advanced computational methods (e.g., all-solvent quantum mechanics (QM), metadynamics (MD), and grand canonical quantum mechanics (GCQM)) combined with experiments are more reliable and realistic for inferring reaction mechanisms.

### Improving Limits of Active Relationships

The conventional AEM limits OER activity due to intrinsic scaling relationships between the adsorption free energies of OOH and OH intermediates. To circumvent these constraints and develop highly active IBEs, several strategies warrant consideration.

1. Optimizing the adsorption energy relationships. Further research into Ir catalytic activity reveals that the existence of dual/multi-sites could overcome the traditional rate-limiting step between OOH and OH, separating the adsorption energy relationship to form a new OER pathway. Additionally, the effects of hydrogen bonding and electrophilic oxygen behavior on the activity relationship should be considered, especially in terms of optimizing the Δ*G* of the OER, which approaches the equilibrium potential of 1.23 eV.
2. Clarification of the activity relationship between LOM and oxygen adsorption energy. Currently, LOM can effectively break the activity relationship limitation by activating O_L_ in IrO_2_, but the specific interaction remains unclear. Thus, the intrinsic mechanism of O_L_ activity needs to be investigated, and the basic activity-related parameters should be clarified to optimize the specific activity-related steps. Furthermore, IrO_*x*_ with O_L_-containing species can consistently provide reactive oxygen species for rapid oxygen exchange, improving the OER mechanism and allowing more active O_L_ to participate in the reaction while maintaining the robust structure.
3. Using artificial intelligence (AI) to explore new OER mechanisms. There is an ongoing need to accelerate the exploration of novel activity relationships for electrocatalysts. High-throughput (HT) techniques integrated with AI can effectively screen and construct experimental methods for material databases. These technologies facilitate the parallel synthesis and characterization of varying parameters, assist in rapidly identifying catalysts with high intrinsic activity, and elucidate correlations between synthesis parameters and activities. Such insights are invaluable for the rational design of promising catalysts. By addressing these aspects, researchers can potentially break the current limitations and enhance the catalytic performance of IBEs significantly.

### Focus on Stability of Catalysts

Long-term stability is paramount for the practical viability and economic advantage of IBEs, especially to meet PEMWE targets of 80 000 h of operation. Efforts to improve stability should prioritize:

1. Maximization of Ir utilization. Combining multi-scale Ir-based catalysts (e.g., single-atom catalysts and nanocluster catalysts), with higher mass activity, not only provides a more diverse atomic coordination environment but also optimizes the number of metal atoms at the metal-support interface. This strategy contributes significantly to the durability of the electrocatalyst.
2. Balancing stability and activity. While LOM facilitates relatively high OER activity, a balance between stability and activity is essential. The structural changes induced by the LOM pathway, such as physical exfoliation, reconstitution, and structural collapse, can lead to diminished durability. Moreover, stability is interconnected with various factors (conductivity, thermodynamics, dissolution rates, crystal structure, etc.), and further investigations are needed to refine the design of IBEs with improved stability.
3. Accurate assessment of stability. Although ASF or S-numbers are widely used as standard indicators for evaluating the stability of various electrocatalysts, the actual stability of PEMWEs suffers from structural changes, catalyst degradation, and particle aggregation. These factors would result in a disparity between the stability observed in half-cell tests and PEMWEs. Consequently, there is an urgent need to develop more appropriate stability criteria and methods to accurately quantify the stability of different OER catalysts. Addressing these challenges will be critical to advancing IBEs capable of sustained, industrial-scale operation.

### Advancing Practical Application of PEMWEs

The IBEs currently serve as benchmark anode catalysts in PEMWEs due to their superior activity and stability. However, the scarcity and high cost of iridium—produced mainly as a by-product of other noble metals—necessitate intensified efforts to improve Ir utilization efficiency and to develop earth-abundant, low-cost transition metal-based catalysts with adequate acid stability. For industrial PEMWE deployment, attention must also be given to system-level components and engineering, including:

1. Introduction of free radical scavengers. The IBEs degradation, often caused by the formation of intermediate peroxides of specific metal ions, reduces the durability of the catalyst and the device. The integration of free radical scavengers can effectively mitigate this issue.
2. Customization of porous electrode structures. An optimally designed porous structure could promote mass and electron transfer between reactants and products, ensuring many active sites are accessible during the OER, thus improving the reaction rate.
3. Development of PEMs. Economical PEMs that offer higher proton transport and durability, along with reduced gas permeability, are essential for advancing PEMWE technology.
4. Optimization of collectors and separation plates. It is critical to utilize low-cost collectors and separators with high corrosion resistance, low ohmic resistance, and efficient mass transfer capabilities to improve overall device performance.
5. Advancement and refinement of electrochemical test technologies. To bridge the significant differences between three-electrode setups and PEMWEs, strategies should combine the simple structure of three-electrode cells with operating conditions similar to those in PEMWEs. In addition, PEMWEs at ultra-high current density, temperature, and pressure necessitate further investigation to ensure robust and efficient performance under these extreme conditions.
